# Chloromethyl Glycosides as Versatile Synthons to Prepare Glycosyloxymethyl‐Prodrugs

**DOI:** 10.1002/chem.202103910

**Published:** 2022-01-24

**Authors:** Hidde Elferink, Willem H. C. Titulaer, Maik G. N. Derks, Gerrit H. Veeneman, Floris P. J. T. Rutjes, Thomas J. Boltje

**Affiliations:** ^1^ Synthetic Organic Chemistry Institute for Molecules and Materials Radboud University Heyendaalseweg 135 6525 AJ Nijmegen The Netherlands; ^2^ PharmaCytics B.V., Pivot Park Kloosterstraat 9 5349 AB Oss The Netherlands

**Keywords:** bioavailability, carbohydrates, glucose, glycosidase e, prodrugs

## Abstract

This work investigates the addition of monosaccharides to marketed drugs to improve their pharmacokinetic properties for oral absorption. To this end, a set of chloromethyl glycoside synthons were developed to prepare a variety of glycosyloxymethyl‐prodrugs derived from 5‐fluorouracil, thioguanine, propofol and losartan. Drug release was studied in vitro using β‐glucosidase confirming rapid conversion of the monosaccharide prodrugs to release the parent drug, formaldehyde and the monosaccharide. To showcase this prodrug approach, a glucosyloxymethyl conjugate of the tetrazole‐containing drug losartan was used for in vivo experiments and showed complete release of the drug in a dog‐model.

## Graphical abstract

**Monosaccharides** were introduced on drug molecules to improve their solubility, metabolic stability and oral bioavailability. A new chloromethyl glycoside synthon was developed to enable the synthesis of numerous monosaccharide prodrugs.

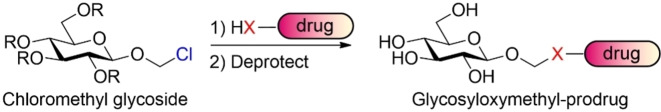

## Introduction

The principal challenge in oral drug administration is achieving a predictable and high level of bioavailability.^[1]^ When a (pre)clinical candidate displays suboptimal pharmacokinetics, carrier‐linked prodrug strategies can be used to increase oral bioavailability.^[2]^ A so‐called ‘promoiety’ is added to improve the pharmacokinetic profile and is ultimately cleaved from the drug via chemical or enzymatic conversion. For example, polar groups such as carboxylic acids (e. g. oseltamivir^[3]^) and phosphates (e. g. adefovir^[4]^) are masked to increase their lipophilicity and to promote passive diffusion over the intestinal membrane. The promoiety is cleaved off by esterases during or after passing the intestinal membrane, thereby releasing the parent drug. Alternatively, the solubility of drug molecules can be increased by the addition of a polar promoiety such as a phosphate. The phosphate group can be cleaved by intestinal alkaline phosphatases excreted from the apical side of the intestinal membrane followed by passive diffusion of the drug (e. g. tedizolid phosphate^[5]^ and fosamprenavir^[6]^). Alternatively, nutrient transporter proteins in the intestinal brush border membrane can be targeted to enhance drug uptake. Marketed examples such as valacyclovir^[7]^ and valganciclovir^[8]^ represent valine ester oral prodrugs and are designed to take advantage of transport via intestinal peptide transporter 1 (PEPT1). The valine ester moiety is cleaved off after transport by endogenous esterases to release the active drugs acyclovir and ganciclovir respectively. Another example is the prodrug gabapentin enacarbil (Horizant™),^[9]^ which appears to be a substrate for both the monocarboxylate transporter MCT‐1 and the multi vitamin transporter SVMT.

**Figure 1:**
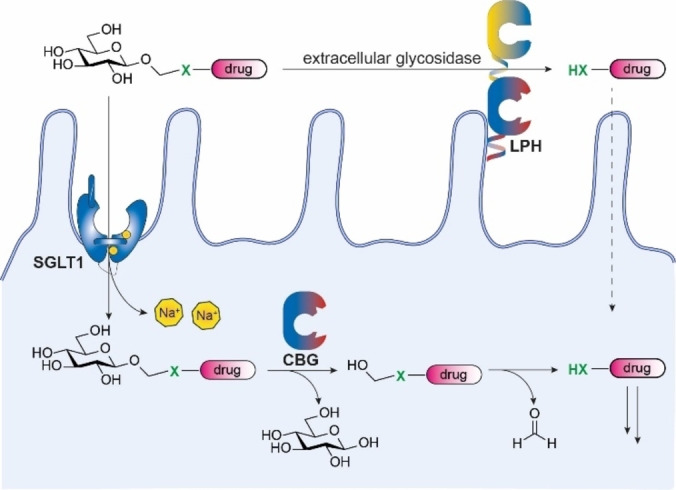
Uptake and hydrolysis of glucosyloxymethyl drug conjugates in enterocytes. SGLT1=sodium dependent glucose transporter 1; LPH=Lactase Phlorizin Hydrolase; CBG=cytosolic beta glucosidase; dotted line=passive diffusion. X=hetero‐atom inherent to the drug molecule.

Monosaccharides (e. g. d‐glucose) represent another attractive group of nutrients to improve the oral bioavailability of drugs. Monosaccharides are polar, non‐toxic and can be transported into the bloodstream by monosaccharide transporters SGLT1 and GLUT2 in the small intestine.^[10]^ In addition, monosaccharide‐drug conjugates based on glucuronic acid have developed to target tumors by their increased glucuronidase expression.^[11]^ Glycosylated flavonoids are known to be more soluble and are taken up more efficiently than their non‐glycosylated counterparts.^[12]^ The parent flavonoid is released either by extra cellular membrane bound β‐glycosidases such as lactase‐phlorizin hydrolase (LPH) or by intracellular human cytosolic β‐glucosidase (hCBG). Inspired by these studies, we investigated the enzymatic release of tripartite drug conjugates consisting of a monosaccharide connected to a drug molecule via a self immolative linker (SIL, Figure 1).

Monosaccharide‐drug conjugates are expected to display improved water solubility and may improve oral bioavailability by two distinct mechanisms. First, active uptake via monosaccharide transporters and subsequent glycosidase mediated release may occur. Second, release of the drug by extracellular intestinal glycosidases present in the brush border or the microbiome may occur followed by passive transport of the drug. hCBG^[13]^ and LPH^[14]^ are the most important β‐glycosidases in the GI‐tract and represent the intracellular and extracellular β‐hydrolase activity, respectively.^[15]^ Since the expression of LPH can alter over lifetime,^[16]^ hCBG is the most attractive and reliable target for drug release. To enable tuning of the drug transport and release kinetics, we selected a panel of monosaccharides that are known substrates for the target transporter (SGLT1) and target glycosidase (hCBG). SGLT1 is able to transport d‐glucose, deoxygenated derivatives such as d‐quinovose and other natural sugars such as d‐galactose.^[17]^ In addition to d‐glucose, hCBG can hydrolyze glycosides of d‐galactose, d‐fucose, l‐arabinose and to a lesser extend d‐xylose.^[13]^ Hence, to tune the transport and release kinetics, we selected a series of six monosaccharides based on d‐glucose and d‐galactose.

The monosaccharides are connected to the drug via a SIL enabling the chemical conjugation of a broad scope of drug chemotypes.^[18]^ Additionally, the interaction between the glycoside and its transporter and/or hydrolase is influenced by the structure of the aglycon attached which in some cases, this can result in poor or no active uptake and/or enzymatic hydrolysis. The *para*‐aminobenzylalcohol (PABA) spacer is frequently used as SIL in prodrug strategies, yet some glucosyl‐PABA prodrug conjugates show poor release by hCBG despite being cleaved by other hydrolases such as bovine β‐glycosidase.^[19]^ In addition, the azaquinone by‐product formed in the release reaction has been reported to react with the enzyme and decrease its activity.^[20]^ Hence, we sought to develop an alternative SIL that shows fast release and yields a non‐toxic by‐product. This led us to the use of the glycosyloxymethyl group as a promoiety. The bisacetal is reported to be stable under acidic conditions^[21]^ and hydrolysis would result in the release of formaldehyde, an endogenous metabolite.^[22]^ The synthesis of glycosyloxymethyl conjugates can be achieved via the reaction of TMS‐glycosides with symmetric formaldehyde acetals or via the electrophilic activation of phenylthiomethyl glycosides.^[23]^ In addition, the synthesis of a bisacetal conjugate from the sugar hemi‐acetal has been reported.^[24]^ Herein, we introduce a new, convenient method to prepare glycosyloxymethyl‐prodrugs using chloromethyl‐glycoside synthons. Glucosyloxymethyl conjugates of various marketed drugs were prepared, their release kinetics by β‐glucosidase was established and a losartan conjugate was tested in vivo.

## Results and Discussion

Six different monosaccharide synthons for the preparation of glycoside bisacetal conjugates were synthesized starting from glycosyl imidates **1 a**–**f**. Glycosylation of phenylthiomethanol with **1 a**–**f** and AgOTf afforded mixtures of the desired thiomethyl *O*,*S*‐acetal (**2 a**–**f**, n=1) and thioglycoside byproducts (**2 a**–**f**, n=0, Table 1). Interestingly, the formation of thioglycoside increased with the glycoside donor reactivity. In principle, activation of thiomethyl acetal **2 a**–**f** (n=1) with a thiophilic promotor and a nucleophile could also lead to methylene bisacetal conjugates. We reasoned that converting the thiomethyl acetal into a leaving group that could be displaced under basic S_N_2 conditions would provide high yields of the methylene bisacetal conjugates with various nucleophiles. Hence, the mixtures of thioglycoside and thiomethyl acetal were treated with sulfuryl chloride. In this process the thioglycosides were converted to the corresponding glycosyl chlorides which hydrolyzed upon work‐up and hence aided purification of the more stable chloromethyl glycosides **3 a**–**f**. In order to evaluate the ability of glucosidases to cleave methylene bisacetal conjugates, we synthesized chromogenic substrates **5 a**–**f** (Table 1). 4‐Nitrophenol was first deprotonated by NaH and reacted with chlorides **3 a**–**f** to afford bisacetal glycosides **4 a**–**f**. Deprotection of the acetylated bisacetal glycosides under Zemplén conditions gave the 4‐nitrophenyloxymethyl glycosides **5 a**–**f** in good yields.

**Table 1:** Synthesis of chloromethyl glycopyranosides. Reagents and conditions: *i)* AgOTf (cat.), phenylthiomethanol, DCM, 0 °C; *ii)* SO_2_Cl_2,_ DCM, rt; *iii*) NaH, NaI (cat.), 4‐nitrophenol, DMF, rt; %; iv) K_2_CO_3_, MeOH, rt; yield reported over two steps; **5 a**, 69 %; **5 b**, 52 %; **5 c**, 65 %; **5 d**, 92 %; **5 e**; 71 %; **5 f**, 63 %.

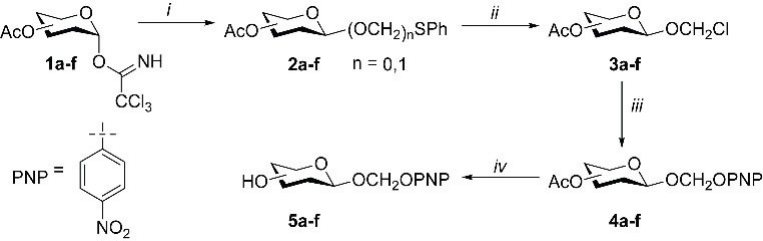			
Entry	Sugar	**2 a**–**f** (n=0/n=1)^[a]^	Yield **3 a**–**f** (% over two steps)^[b]^
**a**	β‐d‐glucose	2/3	48
**b**	β‐d‐quinovose	3/2	36
**c**	β‐d‐xylose	1/2.2	32
**d**	β‐d‐galactose	2.2/1	24
**e**	β‐d‐fucose	2.5/1	25
**f**	α‐l‐arabinose	1.6/1	35

[a] Ratio determined by integration of key signals in the ^1^H NMR spectrum; [b] Isolated yield.

**Figure 2:**
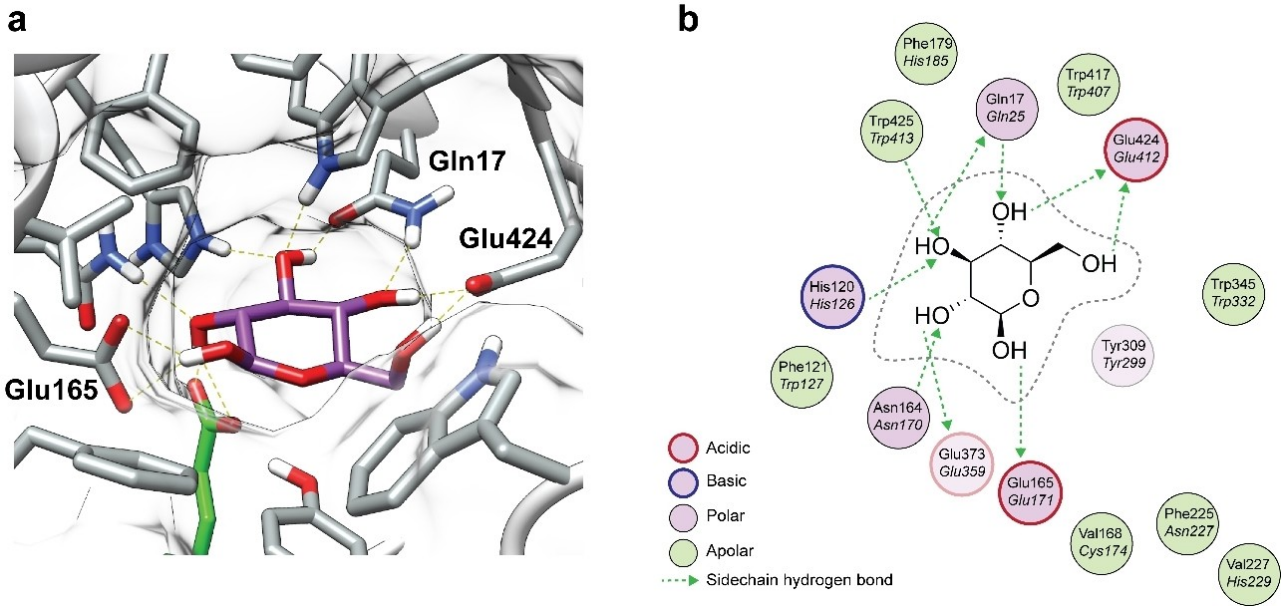
Enzyme substrate interactions. a) 3D image of the interactions of β‐d‐glucose in the glycone pocket of hCBG (2E9L). The hydrogen bond network is shown in green. b) 2D overview of important residues in the glycone and aglycone pocket of hCBG and Abg (italic). Residues that are situated below the substrate are partially transparent.

**Enzyme selection**: Next, we studied the enzymatic conversion of bisacetal glycoside model prodrugs **5 a**–**f**. Ideally, hCBG would be preferred but acquiring hCBG is cumbersome due to its high cost and low stability after recombinant expression. Hence, we selected *Agrobacterium Sp*. (Abg, GH1 family) as an in vitro hCBG‐model enzyme for its high activity and stability.^[25]^ The validity of Abg as a model for hCDG was determined by homology modelling and subsequent superimposition of Abg on an available crystal structure of hCBG in complex with its hydrolysis product β‐d‐glucose^[26]^ (Figure 2A). Similarly, we prepared a homology model for the cytosolic β‐glucosidase from *Canis Lupus Famliaris* (Clg) as most in vivo pharmacokinetic experiments for intestinal uptake are performed in a dog‐animal model.

As expected with a sequence identity of 88 %, the homology model of Clg compares well with hCBG at an overall rmsd value of 0.57 Å after superpositioning. Abg is less homologous to hCBG with a sequence identity of 34 %, though the overall TIM‐barrel fold is conserved with a 1.43 Å rmsd value. In the glycone pocket, all carbohydrate hydrogen bonding residues are conserved between hCBG and Abg. Similarly, most of the apolar surrounding residues are conserved, or substituted for another aromatic apolar residue (Figure 2B). The aglycone pocket however, differs significantly.

Overall, a more hydrophilic aglycone environment for Abg is observed compared to hCBG. In particular, the Phe^225^/Asn^227^ deviation may cause differences in aglycone specificity, since mutants of hCBG developed by Juge and coworkers identified Phe^225^ to be relevant for aglycone recognition.^[13]^ In terms of assessing hydrolysis, the Phe^179^/His^185^ deviation likely biases carboxylate‐bearing aglycones. His^185^ was shown to interact with a carboxylate‐moiety of salicylate β‐d‐glucoside, an endogenous substrate of Abg, in the template used for homology modelling.^[27]^ Overall, the structure of the active site seems conserved between both enzymes when comparing the molecular surfaces making Abg a representative β‐glucosidase for hCBG. Nevertheless, caution should be taken with the extrapolation of the influence of different aglycones in Abg and hCBG.

**Kinetics**: Having identified Abg as a suitable model enzyme, we set out to study the enzymatic hydrolysis of 4‐nitrophenyloxymethyl glycosides **5 a**–**f** using an assay based on absorbance spectroscopy monitoring the release of 4‐nitrophenol (absorbance at 405 nm). Enzymatic reactions were performed in triplo at pH 6.8 (50 mM final concentration) containing 0.1 % bovine serum albumin at 37 °C.^[25]^ Aliquots of 100 μL were taken at constant time periods and added to 100 μL aq. NaOH (0.1 M) in a 96‐well microtitre plate to quench the enzymatic reaction and ensure maximal absorption of sodium 4‐nitrophenolate as outlined in previous studies.^[28]^ The initial rate (v_o_) was determined normalized on the ∼0.1 U/mL enzyme concentration and plotted against substrate concentration [S]. Non‐linear regression analysis based on 7–10 concentrations depending on the glycoside, was used to determine the K_m_ and *k*_cat_ values. *k*_cat_ values were normalized based on the enzyme concentration. In addition, control experiments were performed without enzyme which showed no hydrolysis of the conjugates before or after quenching with NaOH.

The Michaelis‐Menten parameters for both the 4‐nitrophenyl glycosides (**6 a**–**f**) and the 4‐nitrophenyloxymethyl glycosides (**5 a**–**f**) are presented in Table 2. All studied substrates except for bisacetal conjugates of pentoses β‐d‐xyloside **5 c** and pentoses α‐l‐arabinose **5 f**, showed enzymatic release of 4‐nitrophenol. The broad substrate scope was also observed in previous studies by Withers and coworkers. At high concentration, the V_0_ of most substrates such as **6 a**, **6 d** and **6 e** followed a horizontal asymptote as expected in accordance with previously studied substrates. In contrast, quinovoside **6 b** and xyloside **6 c** showed an over expected rate‐enhancement. Careful analysis of the Lineweaver‐Burk plot (see Supporting Figure S3) did not show a single linear line, instead two linear sections were observed which suggests a concentration dependent mechanism. Earlier studies have shown that 4‐nitrophenyl glycosides can act as nucleophile in the deglycosylation reaction at high substrate concentrations to obtain a 4‐nitrophenyl disaccharide. This process is also known as transglycosylation and was also observed by Withers and coworkers working using Abg and 4‐nitrophenyl xylose **6 c** (see Supporting Figure S4 and S5 for NMR and MS‐analysis).^[25]^

**Table 2:**
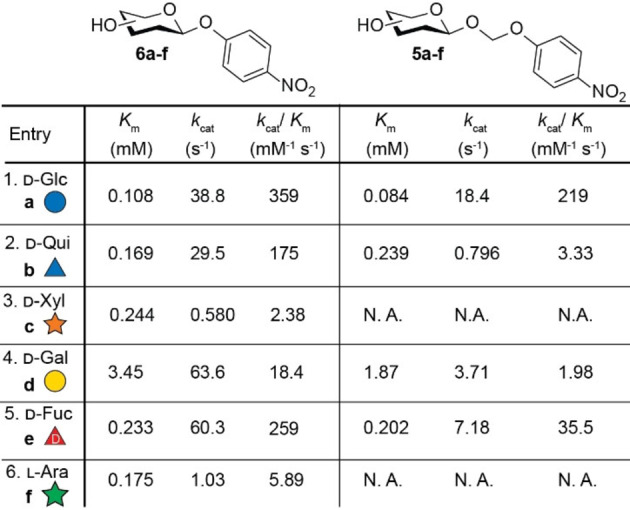
Michaelis‐Menten parameters of substrates **5 a**–**f** and **6 a**–**f**. Values presented are determined with GRAPHPAD 5.0 from the measured Michaelis‐Menten or Lineweaver‐Burke plots (see Supporting Information). N.A: no activity.

In general, the *k*_cat_/*K*_m_ values of 4‐nitrophenyl glycosides **6 a**–**f** follow the same trend as reported previously in literature with the exception of l‐ara. Interestingly, Abg shows a similar substrate scope towards glycoside bisacetals analogues **5 a**–**f** although a significant decrease in *k*_cat_/*K*_m_ is observed for all substrates except d‐glc. This is not surprising since the anomeric 4‐nitrophenyl group is a better leaving group (p*k*_a_=7.16) compared to the anomeric 4‐nitrophenyloxymethyl group (estimated p*k*_a_=11^[29]^). Encouraged by the results summarized in Table 2 we decided to continue with d‐glc as substrate candidate for our prodrug strategy, although other monosaccharides such as d‐fuc might be interesting when a different kinetic profile is desired.

**Figure 3:**
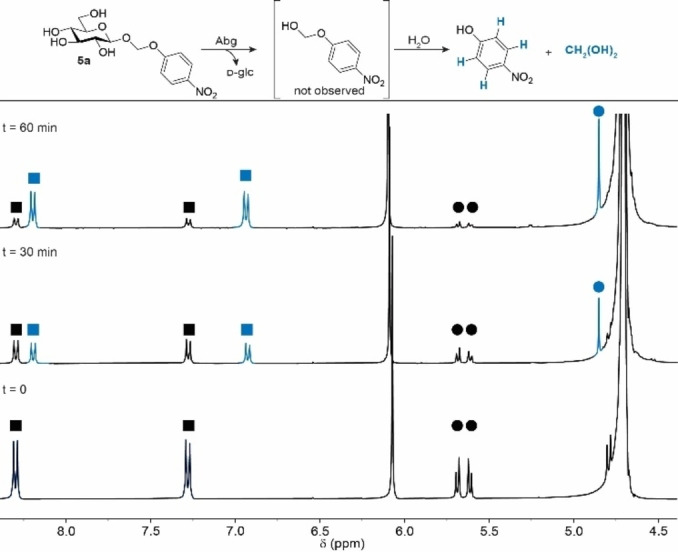
Enzymatic hydrolysis studied by quantitative ^1^H NMR. The hydrolysis of glycosyl conjugates is was studied by tracking key‐signals of the conjugate (in black) and the product (in blue) in the NMR spectrum. Key signals were represented by the aromatic protons (square) and the spacer methylene protons (circle). Reagents and conditions: **5 a** (13 mM) was measured in D_2_O with maleate buffer pD 6.5. Agrobactrium Sp. was added from stock to a final concertation of 1.6 U/mL.

Rapid and complete release of the drug is important in *tripartite* prodrugs because intermediates complicate the overall pharmacokinetic profile. For these reasons, it is crucial to confirm quick dissolution of the linker‐drug conjugate once the monosaccharide is cleaved off. Hence, quantitative 1D NMR experiments were performed to study the (intermediate) products formed after enzymatic incubation of the most promising promoiety candidate: 4‐nitrophenyloxymethyl glucoside **5 a** (see Figure 3). Glucoside **6 a** was used as positive control to determine the optimal enzyme concentration of enzyme concentration (see Supporting Information Figure S6). Key‐signals of the aromatic protons in the ^1^H NMR‐spectrum were used to track the disappearance of the conjugate and appearance of the aglycone over time (see Figure 3). Under optimized conditions, release of 4‐nitrophenol from **6 a** was observed with the appearance of NMR signals at 8.19 ppm and 6.93 ppm corresponding to 4‐nitrophenol (for time‐course experiment see Supporting Information). In addition, signals of the anomeric protons of α‐d‐glucose (5.14 ppm) and β‐d‐glucose respectively (4.55 ppm) were observed. Next, enzymatic incubation of **5 a** also showed release of 4‐nitrophenol (see Figure 3). Close examination of the ^1^H NMR spectrum did not show additional aromatic signals, instead a singlet appeared at 4.86 ppm reflecting the formaldehyde release product methylene glycol.^[30]^ These results suggest instantaneous dissolution of the hemi‐acetal after sugar hydrolysis.

**Prodrug conjugates**: The overall oral bioavailability depends on both drug transport and the drug release, and although monosaccharides other than glucose may be interesting in this respect, we decided to move forward with the synthesis of glucose‐based drug conjugates. We started by improving the yield of the chloromethyl synthon as the aforementioned route yields the thiogylcoside as the main product. Earlier reports on the synthesis of mannose bisacetals employed benzoyl protecting groups instead of acetyl esters.^[31]^ Moreover, we noticed that more reactive monosaccharides provided more of the thioglycoside (see above) and hence we reasoned that lowering the reactivity by benzoylation may improve the yield. Thus, glucose was perbenzoylated and subsequently regioselectively deprotected with hydrazinium acetate to afford the lactol. Next, conversion to the trichloroacetimidate **7** was performed which was used to glycosylate with phenylthiomethanol^[32]^ affording a mixture of phenylthiomethyl glucoside and thioglycoside (ratio of 1.7/1 respectively) which was converted to the chloromethyl glucoside **8** (Table 3a). Indeed, using the benzoyl esters as protecting groups improved the yield of the desired chloromethyl glucoside (Bz 58 % vs. Ac 48 %).

**Table 3:** Synthesis and hydrolysis of glycoside conjugates. a) Synthesis‐route towards glycoside‐drug conjugates. Reagents and conditions: *i)* AgOTf (cat.), phenylthiomethanol, DCM, 0 °C; *ii)* SO_2_Cl_2,_ DCM, rt; 58 % over two steps; *iii*) NaH, NaI (cat.), substrates **a**–**f** or **h**, DMF, rt; iv) K_2_CO_3_, MeOH, rt. b) Substrates used for glycoside conjugation (yield reported over two steps from **8**), conjugation site is highlighted in blue. c) Enzymatic conversion of glycoside‐drug conjugates analyzed by HPLC.

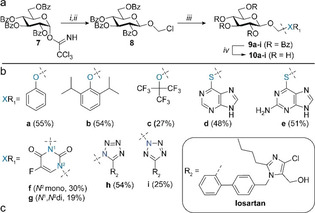					
Entry	Conj.^[a]^	Product release [%]^[b]^			
		0.5 h	1.0 h	2.0 h	24 h
1	**Glc−Ph**	1.9±0.04	3.0±0.30	5.2±0.16	27.7±5.5
2	**10 a**	38.6±2.3	66.0±2.0	89.8±1.7	–
3	**10 b** ^[c]^	1.95±0.3	1.73±0.3	1.66±0.4	–
4	**10 f**	45.8±5.2	65.2±1.5	77.8±9.7	–
5	**10 h** ^[c]^	29.3±0.24	50.4±0.4	77.2±0.7	–
6	**10 i** ^[c]^	16.7±4.8	39.3±2.2	51.3±4	74.7±10

[a] Substrates were incubated at 200 μM in 50 mM phosphate buffer (pH 6.8) in presence of Abg (0.1 U/mL) unless stated otherwise; [b] Release was determined by peak integration from the HPLC‐spectra. [c] Reaction were performed in presence of 5 % ethanol.

With chloromethyl glucoside **8** in hand, we explored its applicability to the conjugation of drugs containing alcohol‐, thiol‐ and amine groups (Table 3b). Reaction of a range of phenols with sodium hydride and **8** afforded the corresponding conjugates moderate to good yield. Hydroxyls such as phenol, perfluoro‐*t*‐butanol and a sterically hindered phenol such as marketed anesthetic propofol could be coupled (**10 a**–**c**). Next, we investigated the coupling of anti‐metabolites 6‐mercaptopurine (6‐MP) and 6‐tioguanine (6‐TG). 6‐MP and 6‐TG are oral drugs used as medication for cancer and autoimmune diseases.^[33]^ Their bioavailability is low and difficult to predict due to extensive first‐pass metabolism to thiouric acid by xanthine oxidase in the intestine and their poor solubility.^[34]^ This makes 6‐MP and 6‐TG attractive candidates for delivery via a prodrug strategy. 6‐MP and 6‐TG drugs were reacted with **8** to afford prodrugs (**10 d**,**e**) in moderate yields. Next, we explored the synthesis of 1,1‐*O,N*‐acetals by coupling **8** to 5‐fluorouracil (5‐FU). 5‐FU is a frequently used anticancer agent however its effectiveness is compromised by its rapid metabolism, non‐specific distribution to cancer cells and low conversion into active metabolites.^[35]^ Marketed prodrugs such as tegafur,^[36]^ doxifluridine^[37]^ and capecitabine^[38]^ have been developed to improve the pharmacokinetics of 5FU. Reaction of **8** with 5FU afforded mono‐ and di substituted conjugates **10 f**,**g** in modest yield. Finally, we synthesized a set of glycosyloxymethyl prodrugs of losartan, a tetrazole containing drug used to treat high blood pressure. Tetrazoles are bioisosters of carboxylic acid groups and therefore of interest for drug discovery.^[39]^ To date, 23 drugs containing tetrazoles are approved by the FDA, most notably a class of hypertensive and anti‐allergic activities acting on the angiotensin receptors. It has been reported that many angiotensin receptor blockers (ARBs) suffer from low and variable bioavailability making them an interesting target for prodrug development. Losartan is an example of angiotensin receptor blocker widely used to treat high blood pressure, with a bioavailability in humans of ∼33 %.^[40]^ Synthesis of glycosyloxymethyl losartan conjugates was achieved under slightly modified conditions to afford a mixture of regioisomers **10 h**,**i** (2.1/1) that could be isolated separately.

With prodrugs **10 a**–**i** in hand we investigated enzymatic drug release using HPLC (Table 3c). Substrates were selected to study the influence of pK_a_ (**10 a**), local steric hindrance (**10 b**), electron‐withdrawing groups (**10 f**) and tetrazole regiochemistry (**10 h** and **10 i**). In addition, we selected phenyl β‐d‐glucopyranoside (**Glc−Ph**) to study the influence of the oxymethyl spacer on the release rate of phenol. Conjugates **10 a**–**b**,**f**,**h**–**i** (200 μM) were incubated with Abg at 37 °C and reactions were quenched at specific time points to monitor drug release (see supporting Figure S7 as example). Since phenol is a worse leaving‐group than 4‐nitrophenol (p*K*_a_ of 9.99 vs. 7.18 respectively), a decrease in the rate of enzymatic conversion is expected compared to **Glc−PNP**. Indeed, HPLC‐analysis showed only 28 % conversion after 24 h (Table 3). In contrast, 90 % release was observed after two hours for phenyloxymethyl analogue **10 a**. This is a twenty‐fold increase in conversion compared to direct conjugate **Glc−Ph**, showcasing the positive effect of the oxymethyl spacer on phenol release. In contrast, the glycosyloxymethyl prodrug conjugate of the anesthetic propofol **10 b** was not hydrolyzed.^[41]^ This is likely due to the steric bulk of the isopropyl groups on the *ortho*‐positions of the phenyl ring, obstructing the substrate from entering the active site of the enzyme. 5FU‐conjugate **10 f** showed fast release of 5‐FU with 46 % released within 30 minutes whereas no degradation was observed in the control experiment. Critically, 5‐FU has been a well‐studied example for prodrug strategy^[42]^ as its oral bioavailability is unpredictable. The glucosyloxymethyl group coupled to 5‐FU may lead to increased metabolic stability and combined with quick release as shown above (Table 3c, entry 6) may afford a more reliable bioavailability profile. The regioisomers of Losartan prodrugs **10 h**–**i** allowed us to probe the effect of local steric hindrance on enzyme hydrolysis. 1‐*H* Losartan conjugate **10 h** was hydrolyzed efficiently whereas the 2‐*H* regioisomer **10 i** was hydrolyzed slower although it is expected to be less sterically hindered.

**In vivo experiments**. Encouraged by these results we selected major product 1‐*H* losartan conjugate **10 h** as a suitable conjugate for an in vivo pharmacokinetics study. To investigate the chemical stability towards conditions in the gastrointestinal tract, we performed pH stability test. **10 h** was stable for 16 h under acidic conditions (pH 1–2) and did not show any release of the parent drug. In addition, the conjugate was stable in formulation (see supporting Figure S8). The pharmacokinetic profile of **10 h** was studied in a dog‐animal model (adult beagle). Losartan or its prodrug **10 h** were administered at 15 μmol/kg using oral gavage and the concentration of losartan in the blood was determined at fixed time points using an LC‐MS‐MS method (see Supporting Information).

**Figure 4:**
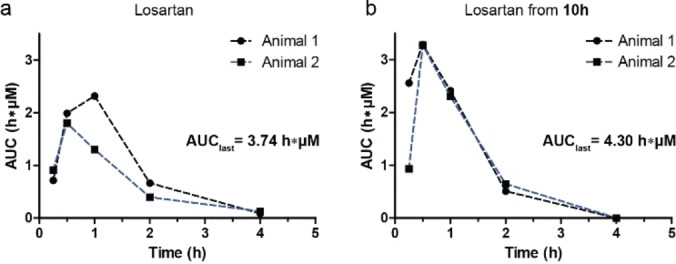
PK‐study of losartan conjugate **19**. The parent compound (panel **a**, losartan) and the prodrug conjugate (panel **b**, **19**) were administered (15 μmol/kg, 1 mL/kg) to female Beagle dogs and circulating concentration of the drug and conjugate were determined at fixed time points. Since no conjugate was detected in the blood circulation, only the concentration of losartan is reported.

The time course of losartan plasma concentrations after oral administration of the prodrug (**10 h**) or the parent are presented in Figure 4. The area under the curve (AUC_last_) is comparable between the parent (3.74 h.μM) and the prodrug (4.30 h.μM) and is in line with previous studies performed on beagles.^[43]^ Because, the limited bioavailability of losartan is related to extensive first‐pass metabolism,^[40]^ these results indicate that the prodrug conjugate is converted before reaching systemic circulation. This is also supported by the complete absence of the intact prodrug in the bloodstream. Interestingly, the maximum concentration (C_max_) of losartan is higher in case of prodrug **10 h**, which suggests rapid uptake and release. Although the in vitro release and stability experiments point towards enzymatic conversion, it has to be noted that under these conditions it is impossible to confirm the exact mechanism of uptake and release. Though it is unlikely that LPH plays a role due to low expression in adult beagles, both passive uptake and active transport (SGLT1) followed by hydrolysis (CBG) are possible. In addition, we cannot rule out the contribution of the microbiome on drug release. It is however encouraging, that no intact conjugate was detected, indicating that losartan was efficiently released from **10 h**. This is an important requirement for prodrug strategy because long circulating intact conjugates complicate the PK‐profile. These observations highlight the potential of the glucoside bisacetal prodrug strategy, though more studies are required, preferably with parent compounds that show lower uptake in the animal model.

## Conclusion

In summary, we introduced the chloromethyl glucoside as a new synthon to prepare bisacetal glucoside prodrugs to improve the poor oral absorption of existing drugs. The glucose moiety is conjugated to the drug of interest via a very small SIL (oxymethyl group), that allows for rapid cleavage by β‐glucosidases releasing formaldehyde, d‐glucose and the drug of interest. The bisacetal glucoside drug conjugates can be synthesized in an S_N_2 fashion from acetylated chloromethyl glucoside **5**, circumventing byproduct formation as observed in previous studies using electrophilic promotor systems. Conjugates of drugs with *O*,*N*‐ and *S*‐chemotypes could be synthesized and were evaluated on enzymatic release using a model β‐glucosidase. To showcase our prodrug approach, we tested a conjugate of marketed tetrazole drug losartan in vivo. Though comparable bioavailability was observed of the parent, absolute release of the parent drug and a higher C_max_ were observed. The results presented in this study highlight the potential of bisacetal glucoside conjugates to improve oral bioavailability and may bring us closer to a versatile prodrug approach to improve the pharmacokinetics of existing and new drugs in general.

## Experimental Section

Please see the Supporting Information for experimental procedures and analytical data. In vivo experiments were performed at the company Charles Rivers (Den Bosch, The Netherlands) in accordance with Dutch law of animal experiments and under approval of the central committee of animal experiments (CCD).

1

## Supporting information

As a service to our authors and readers, this journal provides supporting information supplied by the authors. Such materials are peer reviewed and may be re‐organized for online delivery, but are not copy‐edited or typeset. Technical support issues arising from supporting information (other than missing files) should be addressed to the authors.

Supporting Information

## Data Availability

Research data are not shared.
