# Supplementary material for: Chloromethyl Glycosides as Versatile Synthons to Prepare Glycosyloxymethyl‐Prodrugs
Source: Chemistry. 2022 Jan 24;28(9):e202103910. doi: 10.1002/chem.202103910 (PMC9304170; doi:10.1002/chem.202103910)
Supplement: Supplementary file 1 — Supporting Information [file CHEM-28-0-s001.pdf]

Chemistry–A European Journal

Supporting Information

# Chloromethyl Glycosides as Versatile Synthons to Prepare Glycosyloxymethyl-Prodrugs

Hidde Elferink, Willem H. C. Titulaer, Maik G. N. Derks, Gerrit H. Veeneman, Floris P. J. T. Rutjes, and Thomas J. Boltje*

# Table of contents

Supplementary figures: Enzymatic conversion of 4-nitrophenyl glycosides ....... 2
Supplementary figures: Transglycosylation reactions ....... 4
Supplementary figures. NMR and HPLC examples ....... 5
Supplementary Methods ....... 7
General methods and materials ....... 9
Experimental procedures for the preparation of glycoside conjugates ....... 9
References ....... 19
$^1$H- and $^{13}$C-NMR spectra ....... 21
HPLC traces ....... 49

## Supplementary figures: Enzymatic conversion of 4-nitrophenyl glycosides

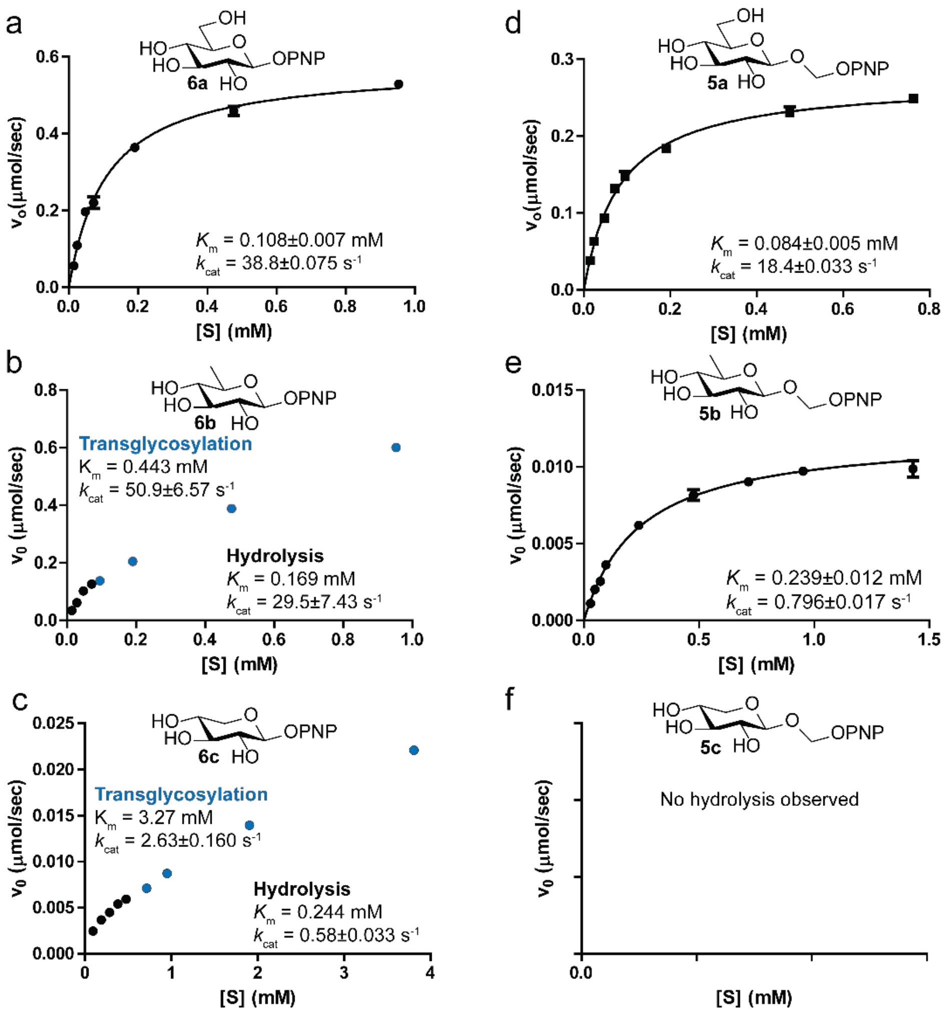


**Fig. S1.** Non-linear regression plots of the hydrolysis of gluco-type sugars by β-glucosidase (*agrobacterium sp.,* Abg). Michaelis-Menten parameters were determined for direct linked 4-nitrophenyl glycosides **6a-c** (panel a-c) and compared to spaced glycosides **5a-c** (panel d-f). In case of **6b** and **26** the $k_{cat}$ and $K_m$ were determined from the corresponding Lineweaver-Burk plot (see **Fig. S3**). Error bars indicate the standard deviations for triplicate measurements.

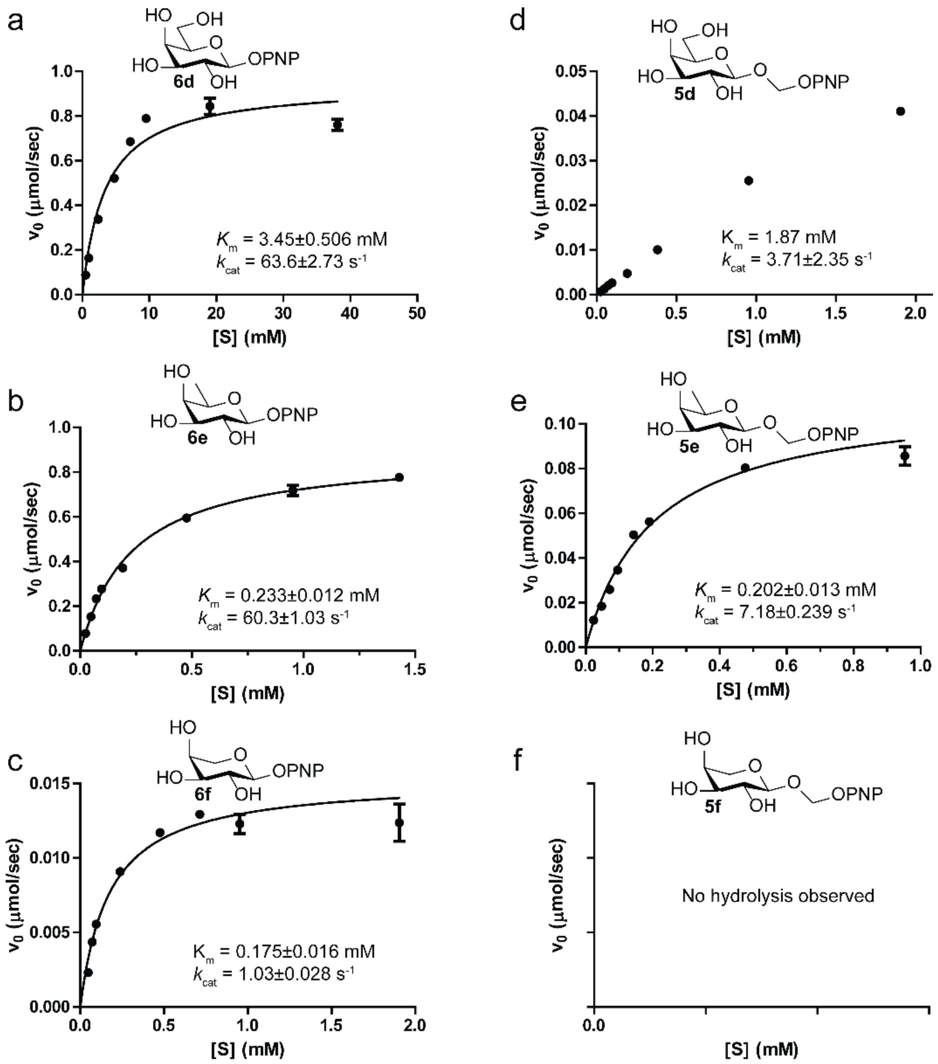


**Fig. S2** Non-linear regression plots of the hydrolysis of galacto-type sugars by Abg. Michaelis-Menten parameters were determined for direct linked 4-nitrophenyl glycosides **6d-f** (panel a-c) and compared to spaced glycosides **5d-f** (panel d-f). In case of **5d** the $k_{cat}$ and $K_m$ were determined from the corresponding Lineweaver-Burk plot. Error bars indicate the standard deviations for triplicate measurements

.

# Supplementary figures: Transglycosylation reactions

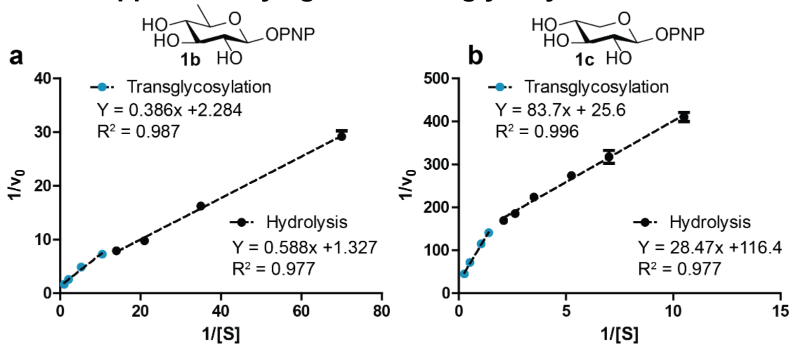


**Fig. S3.** Lineweaver-Burk plot of **1b** (a) and **1c** (b). Linear fits were determined and extrapolated for both the transglycosylation (blue dots) and the hydrolysis (black dots) regions. Error bars indicate standard deviations for triplicate measurements. $1/V_0$ is reported in $\mu M^{-1}sec^{-1}$, 1/[S] is reported in $mM^{-1}$.

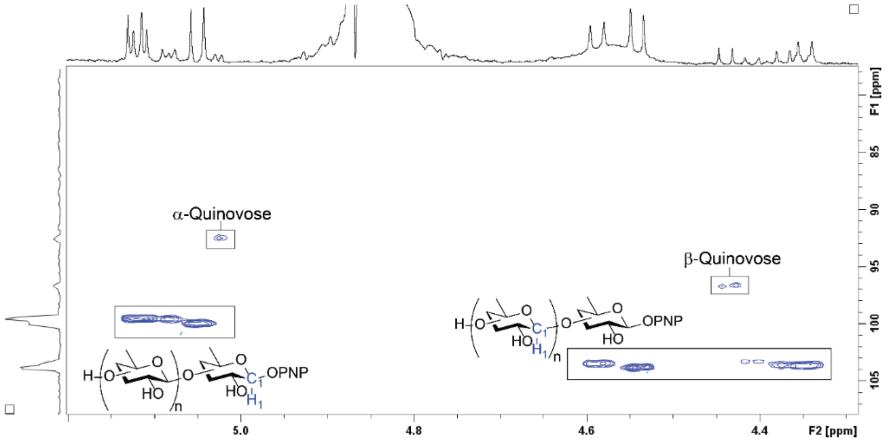


**Fig. S4** $^1H,^{13}C$-HSQC of the 2.0 mM reaction mixture of **6b** incubated with Abg for 2 hours. Anomeric signals are indicated by a rectangular box and when applicable highlighted in blue in the chemical structure presented. n = 0,1. The exact regiochemistry was not determined.

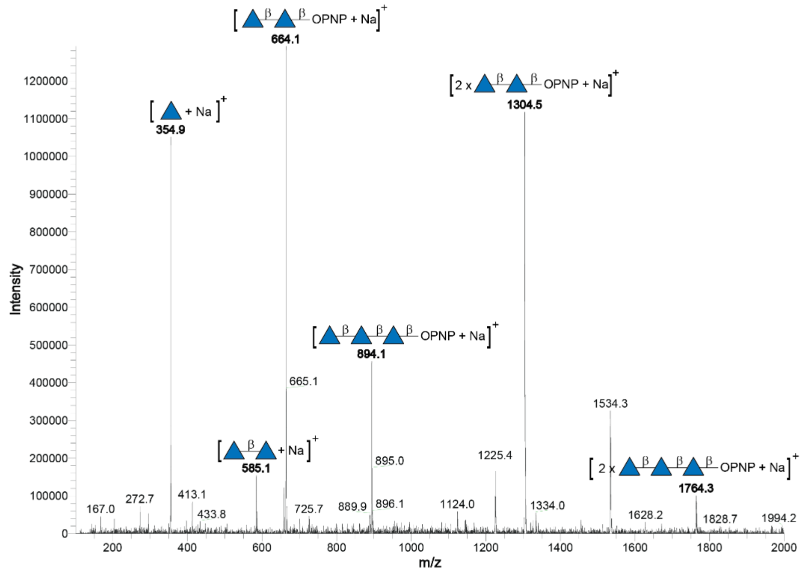


**Fig. S5** ESI-spectrum after acetylation of the 2.0 mM reaction mixture of **6b** incubated with Abg for 2 hours. Masses in bold have been assigned according to their acetylated quinovose derivatives. PNP = 4-nitrophenol.

## Supplementary figures. NMR and HPLC examples

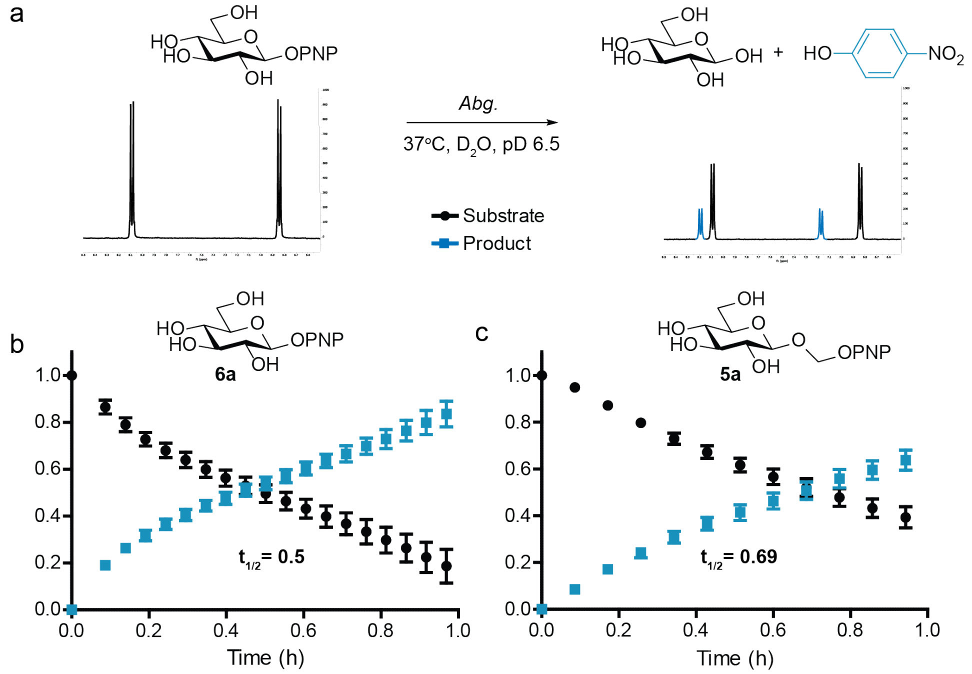


**Fig. S6** Enzymatic hydrolysis studied by quantitative $^{1}$H-NMR**:** a) The hydrolysis of glycosyl conjugates is determined by integrating key-signals of the conjugate (in black) and the product (in blue) in the NMR spectrum. b) The hydrolysis of **6a** was used as positive control to study the spaced derivative **5a** (c). Reagents and conditions: substrates (13 mM) were measured in $D_2O$ with maleate buffer pH 6.5. Abg. was added from stock to a final concertation of 1.6 U/mL. Error bars represent triplicate measurements. Abg, *Agrobacterium sp.*

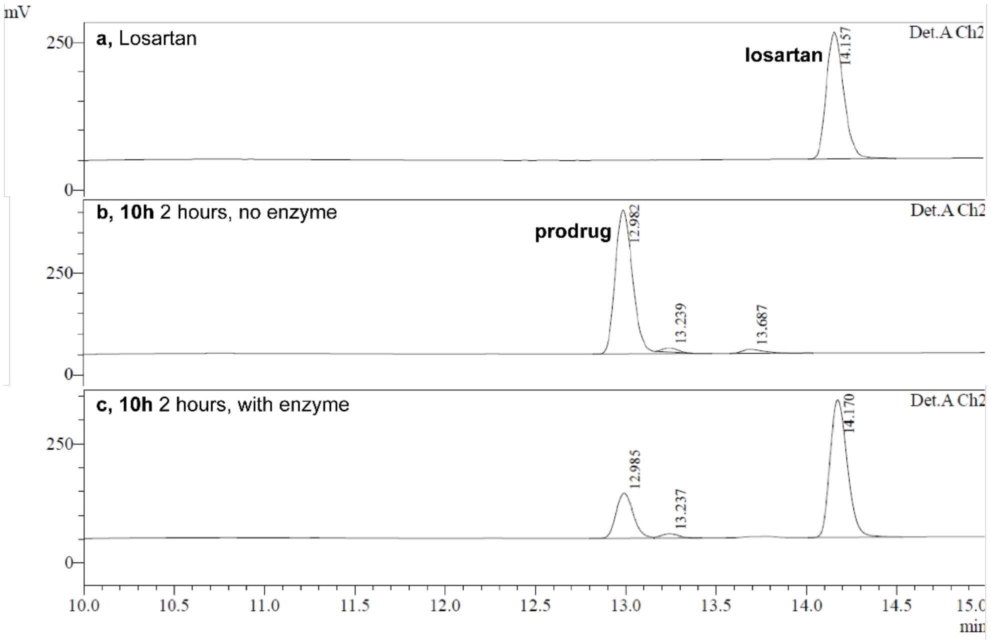


**Fig. S7** Example of prodrug conversion followed by HPLC (215 nm). a) The reference compound losartan. b) Losartan prodrug (**10h**) incubation under reaction conditions without enzyme. c) Reaction mixture with Abg. after two hours incubation.

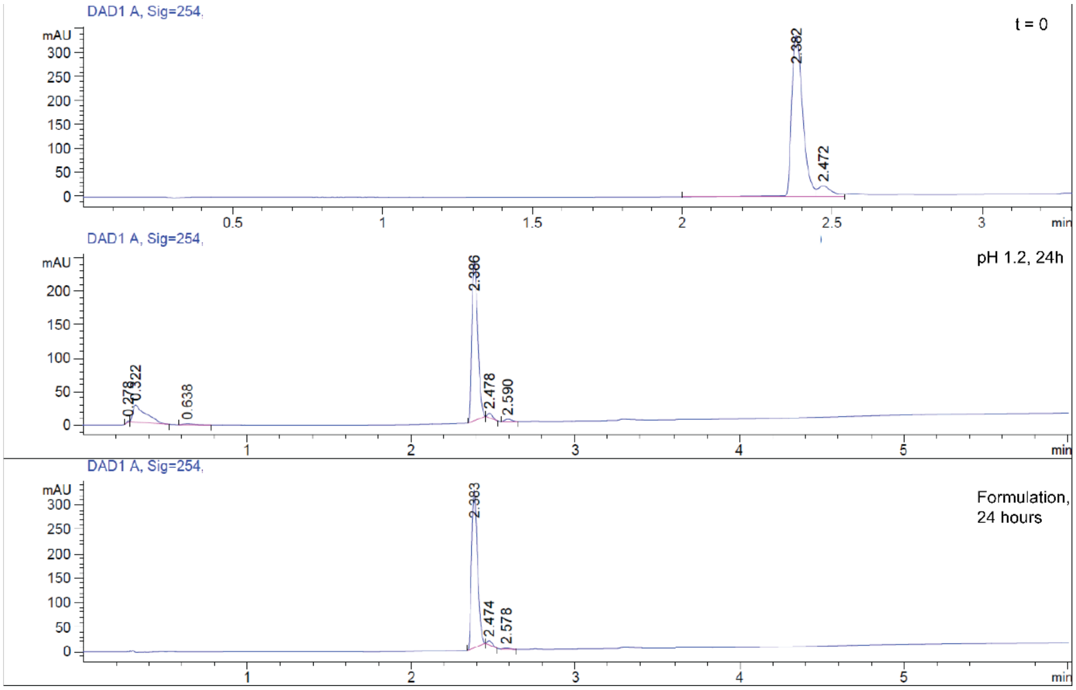


**Fig. S8.** Stability test of **10h**. A batch of **10h** (rt = 2.38 min) was incubated for 24 hours under acidic conditions (pH 1.2) or in the formulation used for *in vivo* experments (a mixture of propylene glycol, ethanol and 0.9% aq. NaCl). Analysis was performed by LC-MS.

## Supplementary Methods

**Homology modelling** The 3D structures of the β-glucosidases of *Agrobacterium Sp.* (Abg, UniProt: 12614) and *Canis Lupus Familiaris* (Clg, Uniprot: f6xby5) were generated with YASARA's homology modelling module[1-6] using standard parameters. The presented models for Abg and Clg were generated using 6RJO[7] and 2E9L[8] respectively as templates, and selected rotamers further refined using the Dunback rotamer library[9] to represent homologous structures for these regions.
The glycone pocket of *Figure 3* was protonated and optimized in YASARA using the AMBER14 forcefield parameters for the protein and AMB1C/GAFF parameters for small molecules[10-13]. Figures were created using Chimera[14] and MOE[15].

**Kinetic evaluation of 4-nitrophenyloxymethyl glycosides.** Enzyme reactions were performed in triplicate. Substrate samples (final volume of 1 mL) were prepared by diluting carefully prepared substrate stock solutions with sodium phosphate buffer pH 6.8 (50 mM final concentration) containing 0.1% bovine serum albumin and milliQ to the desired concentration. The samples were equilibrated at 37°C for at least 5 minutes before the enzyme in 50 mM buffer (2 U per ml, 50 uL) was added. Aliquots of 100 µL were taken at constant time periods and added to 100 µL aq. NaOH (0.1 M) in a 96 well microtiter plate to quench the enzymatic reaction. The absorbance was measured at 405 nm using a Tecan Spark M10 plate reader. Negative assay controls contained no substrate or no enzyme. Rates of the enzymatic reactions were determined at 7 different substrate concentrations from 0.02 – 1.0 mM. $K_m$ and $k_{cat}$ values were determined by nonlinear regression analysis using Graphpad 5.0 (GraphPad Software, San Diego, California USA, www.graphpad.com) for fitting the data. Calibration samples at varying concentration (0.05 – 0.5 mM) were prepared similar to the substrate samples in the enzyme reactions by diluting a carefully prepared 4-nitrophenol stock solution with sodium phosphate buffer pH 6.8 (50 mM final concentration) containing 0.1% bovine serum albumin and milliQ to the desired concentration.

**Studying enzymatic hydrolysis at 200 µM by HPLC analysis.** Substrates samples (1 mL, final concentration 200 µM) were prepared by diluting carefully prepared substrate stock solutions in MilliQ or MilliQ/ethanol mixture with pH 6.8 phosphate buffer (final concentration 50 mM). The samples were temperature equilibrated (37°C) for 15 minutes before the enzymatic reaction was started by addition of 50 µL from an Abg stock solution (2.0 U/ mL) to a final concentration of ~0.1 U / mL. Aliquots were taken at constant time periods and quenched by addition to an equivolume blocking solution (83 mM 1-(3-aminopropyl)imidazole in ACN). The resulting analysis sample was transferred to a glass vial and analyzed by RP-HPLC (gradient 5-95 ACN/$H_2O$, 0.1%TFA). Enzymatic reactions were performed in triplo at 37 °C. In addition, control experiments were performed without enzyme. Unless stated otherwise, no significant degradation of the conjugates was observed in the buffer used nor after quenching in the time period used. Integrals were determined manual using a Labsolutions software package (Shimadzu, 's-Hertogenbosch, The Netherlands).

**Studying enzymatic hydrolysis at 13 mM by quantitative $^1$H-NMR.** Substrates were dissolved in $D_2O$ (maleate buffer, pH = 6.5) to a final concentration of 13 mM. For the $^1$H-NMR internal standards, sulfolane ($T_1$ determined as 9.13 sec.) was used. Dynamic measurements were started by heating the sample in the NMR to 37 °C and recording a $t_0$-spectrum. Subsequently Abg (V = 2.05 uL, 0.82 U, 1.64 U/mL) was added to the sample outside of the NMR. The sample was mixed by shaking and quickly reintroduced to the NMR. The time of enzyme addition was recorded as $t_0$. Spectra were recorded over an appropriate time-scale with the relaxation delay at 3*$T_1$ of the internal standard.

**Determination of oral bioavailability of losartan conjugate 10h.** *In vivo* experiments were performed at the company Charles Rivers (Den Bosch, The Netherlands) in accordance with Dutch law of animal experiments and under approval of the central committee of animal experiments (CCD). Relative and absolute bioavailability may be determined in different animal models and according to different protocols. The following protocol is typical for determining bioavailability in female Beagle dogs. The animals were deprived from food over a time period of 8 h prior to administration and 2 h after administration of the test molecules. Water was supplied without limitation. On the study day, the animals received test molecule **10h** or losartan, at a single dose of 15 µmol/kg, by oral gavage, formulated in mixtures of propylene glycol, ethanol and 0.9% NaCl + 5% mannitol in water. Blood samples were collected from the jugular vein on the

following time points: 0.25, 0.5, 1, 2, 4, 8 and 24 hours after dosing. Circulating concentrations of test compounds were determined over a time period of 24 h using LC/MS/MS methods with demonstrated specificity and error over a concentration range of 1.0 ng/ml (LLQ) to 2500 ng/ml (one-day validation). Pharmacokinetic parameters were calculated from concentration versus time data using non-compartmental pharmacokinetic methods using Phoenix pharmacokinetic software.

## Organic chemistry

**General methods and materials**. Unless stated otherwise, all chemicals were used without further purifications. Solvents were purchased from J. T. Baker, Biosolve, Fischer Scientific and Merck Millipore and used as received. If no further details are given, the reaction was performed under ambient atmosphere and temperature. Molecular sieves (4Å) were flame activated under vacuum prior to use. TLC-analysis was conducted on Silicagel F254 (Merck KGaA) with detection by UV-absorption (λ = 254 nm) where applicable, and by dipping in 10% sulphuric acid methanol followed by charring at ≈300°C. Silica flash column chromatography was performed using Biotage Isolera Spektra One, using Silicycle cartridges (Biotage, 30-100 µm, 60 Å), 4-50 gram or manually using silica gel 60 (Merck, 0.040-0.063 mm). $^{1}H$ and $^{13}C$ NMR spectra were recorded on a Bruker 400 MHz (Avance **III**) or 500 MHz spectrometer (Avance **III** equipped with a Prodigy BB cryoprobe). Unless stated otherwise spectra were recorded in deuterated solvents at a temperature of 26°C. Chemical shifts are reported in parts per million (ppm) relative to tetramethylsilane (TMS), or residual solvents as the internal standard. NMR data is presented as follows: chemical shift, multiplicity (s = singlet, d = doublet, t = triplet, dd = doublet of doublets, m = multiplet and/or multiple resonances), coupling constant in hertz (Hz), integration. All NMR signals were assigned on the basis of $^{1}H$ NMR, $^{13}C$ NMR, COSY and HSQC experiments. Low resolution mass spectra were recorded on a LCQ Advantage Max Finnigan mass spectrometer (Fisher scientific, Bleiswijk, The Netherlands) and high resolution mass spectra (HRMS) on an AccuTOF CS JMS-T100CS (JEOL Ltd., Akishima, Japan). Compounds dissolved in water were purified by lyophilisation (Ilshim Lab co, Ltd. Scala Scientific). Analytical HPLC spectra of compounds were recorded on a Shimadzu LC-20A Prominence system (Shimadzu, ‘s-Hertogenbosch, The Netherlands) equipped with a Gemini NX-C18 column, 150 x 3 mm, particle size 3 µm, pore size 110 Å (Phenomenex, Utrecht, The Netherlands). Gradient used unless stated otherwise is acetonitrile/water 5 – 100%, in 30 minutes and a flow of 0.4 ml/min. In case of conjugate **10f** a Nucleodur HILIC column, 150 x 3 mm, particle size 3 µm (Machery-Nagel, Düren, Germany) with an isocratic eluent (acetonitrile/water, 1/9) was used. Injected conjugates were monitored at 254 nm and 215 nm and the desired peaks were integrated manually using a LabSolutions software package (Shimadzu, ‘s-Hertogenbosch, The Netherlands). β-glucosidase from Abg. (*AgroBac, EC 3.2.1.21*) was obtained from Megazyme[22,23]. Compounds **6a**,**c**-**f**, losartan, 6-mercaptopurine, 6-tioguanine and 5-fluorouracil were purchased from Biosynth Carbosynth.

### Experimental procedures for the preparation of glycoside conjugates

**General procedure 1: Phenylthiomethanol coupling:** Based on a procedure by Crich and coworkers.[16] A solution of the glycosyl imidate (1.0 eq.), phenylthiomethanol (2 eq.) and freshly activated 4 Å molecular sieves in dry DCM (0.100 M) was stirred for 20 min at rt and then cooled to 0 °C. A catalytic amount of AgOTf (26 mg, 0.1 mmol, 0.075 eq.) was added and the reaction mixture stirred at 0 °C for 60 min. Conversion was determined by TLC following the appearance of a UV-active spot coloring brown after sulfuric acid staining. The reaction was then quenched by the addition of sat. aqueous $NaHCO_3$. The mixture was filtered through Celite and the organic layer was washed with aq. NaOH (5%, w/v), brine and concentrated *in vacuo*. The residue was purified by silica gel column chromatography and obtained as a mixture of thioglycoside and phenylthiomethyl glycoside.

**General procedure 2: Chlorination of the phenylthiomethyl acetal:** To a cooled (0˚C) solution of phenylthiomethyl glycoside (1.0 equivalent) in dry DCM (150 mL), sulfuryl chloride (1.1 equivalent) was added. The mixture was stirred under inert atmosphere for 10 min at 0˚C, washed with sat. aq. $NaHCO_3$ (150 mL) and once with brine (150 mL), dried ($MgSO_4$), filtrated and concentrated *in vacuo*. The residue was purified by silica gel column chromatography.

**General procedure 3: Substrate coupling:** 4-nitrophenol (2.0 eq.) was dissolved in DMF (0.20 M) and NaH (2.1 eq., 60 w% in mineral oil) was added. The mixture left under stirring until $H_2$-gass stopped evolving after which the chloromethyl glycoside (1.0 eq.) was added in one portion. The reaction was left under stirring for 1.5 hours after which it was poored into $Et_2O$ and washed with aq. NaOH (1.0 M), $H_2O$ and brine. The organic layer was collected, dried ($MgSO_4$), filtered and concentrated *in vacuo*. The residue was purified by silica column chromatography.

**General procedure 4: Deacylation:** To a suspension of acetylated sugar in MeOH (0.05 M), $K_2CO_3$ (0.1 eq) was added. The reaction was stirred to completion and was neutralized by addition of Dowex $H^+$. The mixture was filtered over a cotton plug, concentrated *in vacuo* and freezedried from $H_2O$ to obtain the final product.

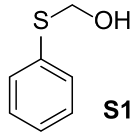

**S1**

**Phenylthiomethanol** (**S1**)[17] : To a mixture of paraformaldehyde (3.0 g, 99.8 mmol) and thiophenol (10.3 mL, 11.0 g, 99.8 mmol) a catalytic amount of NaOMe (0.03 mL, 30 w% in methanol) was heated at 110 °C for 30 min. The solution was subsequently cooled, yielding phenylthiomethanol as a pale-yellow oil (93%). **$^1$H NMR** (400 MHz, $CDCl_3$) δ 7.52-7.48 (m, 2H, 2 x C*H* Ar), 7.35 – 7.30 (m, 2H, 2 x C*H* Ar), 7.29 – 7.25 (m, 1H, Ar, C*H* Ar), 5.02 (d, *J* = 7.1 Hz, 2H, 2 x C*H*H), 2.14 (t, *J* = 7.2 Hz, 1H, $CH_2OH$); **$^{13}$C NMR** (101 MHz, $CDCl_3$) δ 134.62, 130.62, 129.15, 127.21, 68.36 (*C*$H_2$ spacer).

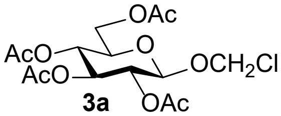

**3a**

**Chloromethyl 2,3,4,6-tetra-*O*-acetyl-β-D-glucopyranoside (3a):** First, the phenylthiomethyl glucooside was prepared v*ia* general procedure 1 starting from 2,3,4,6-tetra-*O*-acetyl-α-D-glucopyranosyl trichloroacetimidate (**1a**)[18]. Next, to a cooled (0˚ C) solution of phenylthiomethyl 2,3,4,6-tetra-*O*-acetyl-β-D-glucopyranoside (**2a**) (4.1 g, 8.7 mmol) in dry DCM (150 mL), sulfuryl chloride (1.38 mL, 17 mmol) was added. The mixture was stirred under inert atmosphere for 10 min at 0˚C, washed with sat. aq. $NaHCO_3$ (150 mL) and once with brine (150 mL), dried ($MgSO_4$), filtrated and concentrated *in vacuo*. Silicagel flash column chromatography (EtOAc/n-heptane, 2/3, v/v) of the residue afforded **3a** (2.12 g, 61%) as a white solid. **TLC:** (EtOAc/n-heptane, 1/1, v/v): $R_f$ = 0.5; **$^1$H NMR** (500 MHz, $CDCl_3$) δ 5.56 (d, *J* = 6.3 Hz, 1H, -OC*H*HCl), 5.49 (d, *J* = 6.4 Hz, 1H, -OCH*H*Cl), 5.26 (t, *J* = 9.5 Hz, 1H, H-3), 5.11 (t, *J* = 9.7 Hz, 1H, H-4), 5.05 (t, *J* = 9.6, 8.0 Hz, 1H, H-2), 4.88 (d, *J* = 8.0 Hz, 1H, H-1), 4.28 (dd, *J* = 12.4, 4.7 Hz, 1H, H-6a), 4.16 (dd, *J* = 12.4, 2.4 Hz, 1H, H-6b), 3.78 (ddd, *J* = 10.0, 4.7, 2.4 Hz, 1H, H-5), 2.09 (s, 3H, C*H$_3$* Ac), 2.07 (s, 3H, C*H$_3$* Ac), 2.03 (s, 3H, C*H$_3$* Ac), 2.01 (s, 3H, C*H$_3$* Ac); **$^{13}$C NMR** (126 MHz, $CDCl_3$) δ 170.72, 170.27, 169.55, 169.52, 96.83 (C-1), 77.28 (-O*C*$H_2$Cl), 72.63 (C-3), 72.52 (C-5), 70.58 (C-2), 68.18 (C-4), 61.72 (C-6), 20.85, 20.78, 20.72, 20.72; **HRMS** (m/z): $[M+Na]^+$ calcd for $C_{15}H_{21}ClO_{10}$, 419.0721; found, 419.0718.

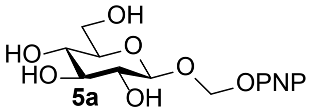

**5a**

**4-Nitro-phenyloxymethyl β-D-glucopyranoside (5a): 5a** was synthesized in a two-step procedure. First, 4-nitrophenyloxymethyl 2,3,4,6-tetra-*O*-acetyl-β-D-glucopyranoside (**4a**) was prepared, v*ia* general procedure 3 starting from **3a** (50 mg, 0.13 mmol). Silicagel flash column chromatography of the residue afforded acetylated precursor **4a** (46 mg, 73%) as a white amorphous solid (**TLC**: (EtOAc/*n*-heptane, 2/3, v/v): $R_f$ = 0.21) and was directly used for the synthesis of **5a**. **5a** was prepared *via* general procedure 4 starting from **4a** (15 mg, 0.030 mmol). The product **7a** (9.5 mg, 95%) was obtained as a white amorphous solid. **TLC** (MeOH/DCM, 1/9, v/v): $R_f$ = 0.25; **HPLC**: Rt. 14.923; **$^1$H NMR** (400 MHz, MeOD) δ 8.41 – 7.99 (m, 2H, 2 x C*H* Ar), 7.35 – 7.01 (m, 2H, 2 x C*H* Ar), 5.65 (d, *J* = 7.1 Hz, 1H, C*H*H spacer), 5.47 (d, *J* = 7.1 Hz, 1H, C*H*H spacer), 4.60 (d, *J* = 7.8 Hz, 1H, H-1), 3.88 (dd, *J* = 11.9, 1.7 Hz, 1H, H-6A), 3.67 (dd, *J* = 11.9, 5.3 Hz, 1H, H-6B), 3.39 – 3.24 (m, 4H, H-3, H-4, H-5), 3.23 (t, *J* = 8.3 Hz, 1H, H-2); **$^{13}$C NMR** (101 MHz, MeOD) δ 163.89, 143.73, 126.56, 117.69, 100.86 (C-1), 91.12 (*C*$H_2$ spacer), 78.36 (C-3), 77.91 (C-4), 74.78(C-2), 71.51 (C-5), 62.71 (C-6); **HRMS** (m/z): $[M+Na]^+$ calcd for $C_{13}H_{17}O_9$, 354.0801; found, 354.0812.

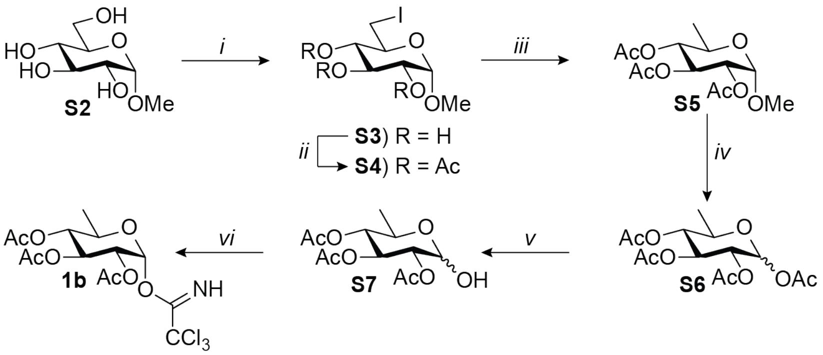


**Supplementary Scheme 3.1:** Synthesis route towards D-quinovoside **1b**. Reagents and conditions: i) $I_2$, $PPh_3$, imidazole, toluene. ii) $Ac_2O$, pyridine; iii) $NaBH_4$, DMSO; iv) $H_2SO_4$ (cat.), $Ac_2O$, AcOH; **S6**, 28% over 4 steps. v) Dimethylaminopropylamine (DMAPA), THF; **S7**, 82%; vi) trichloroacetonitrile, DBU (cat.), DCM; **1b**, 71%.

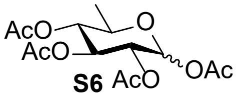

**1,2,3,4-Tetra-*O*-acetyl-α/β-D-quinovopyranoside (S6)[19]: S6** was prepared based on a combination of literature procedures. Methyl α-D-glucopyranoside **S2** (10 g, 51.5 mmol) was suspended in toluene (500 mL).[20] Next, imidazole (10.5g, 154 mmol), triphenylphosphine (20.3 g, 77.2 mmol) and iodine (18.3 g, 72.1 mmol) were added consecutive. The mixture was heated to reflux and stirred for 1 hour to completion. The mixture was allowed to cool down and $H_2O$ (100 mL) was added and stirred for 15 minutes. The organic phase was subsequently extracted with $H_2O$ (150 mL, three times). The aqueous layers were collected and concentrated *in vacuo* to yield the crude methyl 6-iodo-α-D-glucopyranoside (**S3**) which was directly acetylated by addition of pyridine (100 mL) and $Ac_2O$ (50 mL). The mixture was stirred overnight to completion and concentrated *in vacuo* by co-evaporation with toluene. The crude was crystalized from ethanol to obtain methyl 2,3,4-tri-*O*-acetyl-6-iodo-α-D-glucopyranoside (**S3**, 18.0 g, 81% over two steps). Next, **S4** (10.0 g, 23 mmol) was dissolved in DMSO (120 mL) and $NaBH_4$ (4.4 g, 120 mmol) was added under stirring.[21] The resulting mixture was heated to 80°C for 8 hours after which it was poured into ice water (1.0 L). The mixture was extracted with $Et_2O$ (3 x 200 mL) and the combined organic layers were washed with $H_2O$ (200 mL). The organic layer was dried ($MgSO_4$), filtered and concentrated *in vacuo* to obtain methyl 2,3,4-tri-*O*-acetyl-α-D-quinovopyranoside (**S5**, 2.5 g, 35%). Finally, **S5** (2.0 g, 6.6 mmol) was dissolved in a mixture of $Ac_2O$ (10 mL) and AcOH (10 mL) after which $H_2SO_4$ (0.100 mL) was added. The mixture stirred to completion at 0°C for two hours.[19] The mixture was added to a solution of sat. aq. $NaHCO_3$ and extracted with ethyl acetate (3 x 20 mL). The organic layer was dried ($MgSO_4$) and concentrated *in vacuo* to obtain **S6** (2.15 g, 98%) as a mixture of α/β-diastereoisomers. The pure α-product could be obtained by crystallization from ethanol. **TLC**: (EtOAc/*n*-heptane, 3/7, v/v): $R_f$ = 0.26; **$^1$H NMR** (α-product, 400 MHz, $CDCl_3$) δ 6.24 (d, *J* = 3.0 Hz, 1H, H-1), 5.40 (t, *J* = 9.8 Hz, 1H, H-3), 5.04 (dd, *J* = 10.1, 3.2 Hz, 1H, H-2), 4.83 (t, *J* = 9.7 Hz, 1H, H-4), 3.99 (add, *J* = 9.6, 6.0 Hz, 1H, H-5), 2.14 (s, 3H, C*H₃* Ac), 2.03 (s, 3H, C*H₃* Ac), 2.00 (s, 3H, C*H₃* Ac), 1.98 (s, 3H, C*H₃* Ac), 1.18 (d, *J* = 6.0 Hz, 3H, 3 x H-6); **$^{13}$C NMR** (101 MHz, $CDCl_3$) δ 170.35, 169.80, 169.75, 169.11, 89.18 (C-1), 73.32 (C-4), 69.92 (C-3), 69.69 (C-2), 67.96 (C-5), 20.98, 20.77, 20.74, 20.56, 17.40 (C-6).

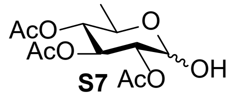

**2,3,4-Tri-*O*-acetyl-α/β-D-quinovopyranoside (S7)[22]:** Based on a procedure by Andersen and coworkers.[18] To a solution of **S6** (1.0 g, 3.0 mmol) in THF (15 mL), DMAPA (1.9 mL, 15 mmol) was added. The reaction was stirred at rt for 1 hour after which it was diluted with DCM (100 mL), washed with 1.0 M aq. HCl (2 x 50 mL) and brine (50 mL). The organic layer was dried ($MgSO_4$), filtered and concentrated *in vacuo*. The residue was purified by silica flash column chromatography (40% EtOAc in *n*-heptane) to give the product **S7** (0.72 g, 82%) as a mixture of α/β-diastereoisomers (1.9/1) and as a clear oil. **TLC**: (EtOAc/*n*-heptane, 2/3, v/v): $R_f$ = 0.20; **$^1$H NMR** (400 MHz, $CDCl_3$) δ 5.54 – 5.42 (m, 1H, H-3 **α**), 5.38 (d, *J* = 3.5 Hz, 1H, H-1 **α**), 5.19 (t, *J* = 9.6 Hz, 0.6H, H-3 **β**), 4.92 – 4.75 (m, 3.1H, H-2 **α**, H-4 **α**, H-4 **β**, H-2 **β**), 4.69 (d, *J* = 7.7 Hz, 0.6H, H-1 **β**), 4.15 (dq, *J* = 10.2, 6.5 Hz, 1H, H-5 **α**), 3.80 (s, 0.6H, 1-O*H* **β**), 3.61 (dq, *J* = 9.8, 6.2 Hz, 0.6H, H-5 **β**), 3.34 (s, 1H, 1-O*H* **α**), 2.07 (s, 1.8H, C*H₃* Ac **β**), 2.07 (s, 3H, C*H₃* Ac **α**), 2.03 (s, 3H, C*H₃* Ac **α**), 2.03 (s, 1.8H, C*H₃* Ac **β**), 2.00 (s, 1.8H, C*H₃* Ac **β**), 2.00 (s, 3H, C*H₃* Ac **α**), 1.24 (d, *J* = 6.2 Hz, 1.8H, 3 x H-6 **β**), 1.17 (d,

*J* = 6.3 Hz, 3H, 3 x H-6 **α**); **$^{13}$C NMR** (101 MHz, $CDCl_3$) δ 171.18, 170.42, 170.39, 170.35, 170.10, 169.94, 95.45 (C-1, **β**), 90.14 (C-1, **α**), 73.90 (C-4, **β**), 73.88 (C-4, **α**), 73.63 (C-2, **β**), 72.37 (C-3, **β**), 71.69 (C-2, **α**), 70.40 (C-5, **α**), 69.99 (C-3, **α**), 65.21 (C-5, **α**), 20.88, 20.87, 20.85, 20.80, 20.76, 17.50 (C-6, **β**), 17.40 (C-6, **α**).

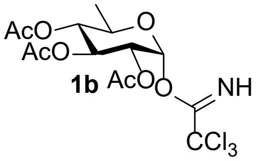

**1b**

**2,3,4-tri-*O*-acetyl-α-D-quinovopyranosyl trichloroacetimidate (1b)[23]:** To a solution of **S7** (0.72 g, 2.48 mmol) and trichloroacetonitrile (2.49 mL, 24.8 mmol) in DCM (5 mL), DBU (37 µL, 0.25 mmol) was added at 0ºC under inert atmosphere. The mixture stirred at rt for 1 hour after which the solution was filtered through a short patch of celite. The celite was flushed with DCM (10 mL) and the combined organic layers were concentrated *in vacuo*. Silica flash column chromatography of the residue (25% EtOAc in *n*-heptane) afforded **1b** (760 mg, 71%) as a white solid; **TLC**: (EtOAc/*n*-heptane, 3/7, v/v): $R_f$ = 0.58. **$^1$H NMR** (400 MHz, $CDCl_3$) δ 8.63 (s, 1H, N*H* imidate), 6.49 (d, *J* = 3.7 Hz, 1H, H-1), 5.53 (t, *J* = 9.9 Hz, 1H, H-3), 5.09 (dd, *J* = 10.2, 3.7 Hz, 1H, H-2), 4.89 (t, *J* = 9.8 Hz, 1H, H-4), 4.21 – 4.04 (m, 1H, H-5), 2.06 (s, 3H, C*H₃* Ac), 2.03 (s, 3H, C*H₃* Ac), 2.01 (s, 3H, C*H₃* Ac), 1.23 (d, *J* = 6.2 Hz, 3H, 3 x H-6); **$^{13}$C NMR** (101 MHz, $CDCl_3$) δ 170.21, 170.08, 169.93, 161.16, 93.18 (C-1), 73.27 (C-4), 70.31 C-2), 69.97 (C-3), 68.26 (C-5), 20.86, 20.83, 20.62, 17.44 (C-6).

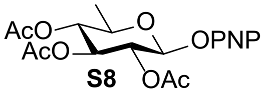

**S8**

**4-Nitrophenyl 2,3,4-tri-*O*-acetyl-β-D-quinovopyranoside (S8):** To a mixture of **1b**[23] (100 mg, 0.23 mmol), 4-nitrophenol (48 mg, 0.35 mmol), molecular sieves (4Å) in dry DCM (2.3 mL) was added $BF_3.Et_2O$ (5.8 uL, 0.046 mmol). The mixture was stirred at 0ºC for 30 minutes and quenched by addition of triethylamine (50 uL). The mixture was diluted with DCM (20 mL) and washed twice with aq. NaOH (1.0 M) and once with brine. The organic layer was dried ($MgSO_4$), filtered and concentrated *in vacuo*. Silica flash column chromatography (30% EtOAc in *n*-heptane) of the residue afforded **S1** (74 mg, 78%) as white amorphous solid. **TLC**: (EtOAc/*n*-heptane, 2/3, v/v): $R_f$ = 0.36; **$^1$H NMR** (400 MHz, $CDCl_3$) δ 8.27 – 8.15 (m, 2H, 2 x C*H* Ar), 7.11 – 7.00 (m, 2H, 2 x C*H* Ar), 5.34 – 5.23 (m, 2H, H-2, H-3), 5.23 – 5.15 (m, 1H, H-1), 5.00 – 4.87 (m, 1H, H-4), 3.79 (dq, *J* = 9.7, 6.2 Hz, 1H, H-5), 2.07 (s, 3H, C*H₃* Ac), 2.05 (s, 3H, C*H₃* Ac), 2.04 (s, 3H, C*H₃* Ac), 1.31 (d, *J* = 6.2 Hz, 3H, 3 x H-6); **$^{13}$C NMR** (126 MHz, $CDCl_3$) δ 170.40, 169.72, 169.39, 161.46, 143.31, 125.98, 116.68, 98.12 (C-1), 72.96 (C-4), 72.62 (C-2), 71.44 (C-3), 70.84 (C-5), 20.81, 20.76 (2C), 17.58 (C-6). **HRMS** (m/z): $[M+Na]^+$ calcd for $C_{18}H_{21}NO_{10}$, 434.1063; found, 434.1067.

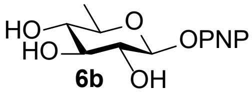

**6b**

**4-Nitrophenyl β-D-quinovopyranoside (6b):** *Via* general procedure 4 starting from **S8** (36 mg, 0.072 mmol). The product **6b** (32 mg, 81%) was obtained as a white amorphous solid. **TLC**: (MeOH/DCM, 1/9, v/v): $R_f$ = 0.37; **HPLC**: Rt. 17.369; **$^1$H NMR** (500 MHz, MeOD) δ 8.26 – 8.17 (m, 2H, 2 x C*H* Ar), 7.30 – 7.11 (m, 2H, 2 x C*H* Ar), 5.05 (d, *J* = 7.5 Hz, 1H, H-1), 3.56 (dq, *J* = 9.4, 6.2 Hz, 1H, H-5), 3.52 – 3.40 (m, 2H, H-2, H-3), 3.10 (t, *J* = 9.1 Hz, 1H, H-4), 1.32 (d, *J* = 6.2 Hz, 3H, 3 x H-6); **$^{13}$C NMR** (126 MHz, MeOD) δ 163.85, 143.87, 126.62, 117.58, 101.35 (C-1), 77.53 (C-3), 76.65 (C-4), 74.95 (C-2), 73.67 (C-5), 18.05 (C-6).

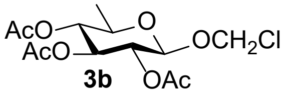

**3b**

**Chloromethyl 2,3,4-tri-*O*-acetyl-β-D-quinovopyranoside (3b):** First the phenylthiomethyl quinovoside was prepared v*ia* general procedure 1 starting from 2,3,4-tri-*O*-acetyl-α-D-quinovopyranosyl trichloroacetimidate[23] (500 mg, 1.19 mmol). The residue was purified by silica column chromatography (30% EtOAc in n-heptane) as an inseparable mixture of the spaced- and directly coupled thiophenyl glycoside (3/2, 440 mg). **TLC**: (EtOAc/*n*-heptane, 3/7, v/v): $R_f$ = 0.41. The mixture was directly used for chlorination using general procedure 2. Silica column chromatography of the residue obtained **3b** (144 mg, 36 % over two steps) as white amorphous solid. **TLC**: (EtOAc/*n*-heptane, 3/7, v/v): $R_f$ = 0.40; **$^1$H NMR** (400 MHz, $CDCl_3$) δ 5.57 (d, *J* = 6.2 Hz, 1H, -CH*H*Cl), 5.49 (dd, *J* = 6.3, 0.5 Hz, 1H, -CH*H*Cl), 5.21 (t, *J* = 9.5 Hz, 1H, H-3), 5.01 (dd, *J* = 9.7, 8.1 Hz, 1H, H-2), 4.88 – 4.78 (m, 2H, H-1, H-4), 3.65 (dq, *J* = 9.7, 6.2 Hz, 1H, H-5), 2.06 (s, 3H, C*H₃* Ac), 2.05 (s, 3H, C*H₃* Ac), 2.01 (s, 3H, C*H₃* Ac), 1.26 (d, *J* = 6.2 Hz, 3H, 3 x H-6); **$^{13}$C NMR** (101 MHz, $CDCl_3$) δ 170.38, 169.82, 169.68, 96.72 (C-1), 77.36 (-$CH_2$Cl), 73.24 (C-4), 72.67 (C-3), 71.00 (C-2), 70.74 (C-5), 20.82, 20.81, 20.76, 17.41 (C-6); **HRMS** (m/z): $[M+Na]^+$ calcd for $C_{13}H_{19}ClO_8$, 361.0666; found, 361.0677.

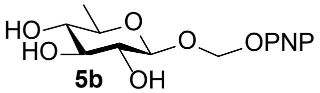

5b

**4-Nitrophenyloxymethyl β-D-quinovopyranoside (5b): 5b** was synthesized in a two-step procedure. First, 4-nitrophenyloxymethyl 2,3,4-tri-*O*-acetyl-β-D-quinovopyranoside (**4b**) was prepared *via* general procedure 3 starting from **3b** (50 mg, 0.15 mmol). Silicagel flash column chromatography of the residue afforded **4b** (45 mg, 69%) as a white amorphous solid (**TLC**: (EtOAc/*n*-heptane, 2/3, v/v): $R_f$ = 0.31) and was directly used for the synthesis of **5b**. **5b** was prepared *via* general procedure 4 from **4b** (30 mg, 0.068 mmol). Silicagel flash column chromatography of the residue (7% MeOH in DCM) afforded the product (16 mg, 75%) as a white amorphous solid. **TLC**: (MeOH/DCM, 1/9, v/v): $R_f$ = 0.25; **HPLC**: Rt. 17.365; **$^1$H NMR** (500 MHz, MeOD) δ 8.23 – 8.17 (m, 2H, 2 x C*H* Ar), 7.29 – 7.22 (m, 2H, 2 x C*H* Ar), 5.57 (d, *J* = 7.2 Hz, 1H, C*H*H spacer), 5.46 (d, *J* = 7.2 Hz, 1H, C*H*H spacer), 4.56 (d, *J* = 7.8 Hz, 1H, H-1), 3.38 – 3.25 (m, 2H, H-3, H-5), 3.21 (dd, *J* = 9.3, 7.8 Hz, 1H, H-2), 3.00 (t, *J* = 9.1 Hz, 1H, H-4), 1.26 (d, *J* = 6.2 Hz, 3H, 3 x H-6); **$^{13}$C NMR** (126 MHz, MeOD) δ 163.88, 143.71, 126.54, 117.68, 101.17 (C-1), 91.34 (C$H_2$ spacer) , 77.62 (C-3), 76.85 (C-4), 75.04 (C-2), 73.66 (C-5), 18.00 (C-6); **HRMS** (m/z): $[M+Na]^+$ calcd for $C_{13}H_{17}NO_8$, 338.0852; found, 338.0879.

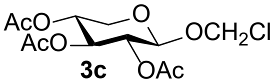

3c

**Chloromethyl 2,3,4-tri-*O*-acetyl-β-D-xylopyranoside (3c):** First the phenylthiomethyl xyloside was prepared v*ia* general procedure 1 starting from 2,3,4-tri-*O*-acetyl-α-D-xylopyranosyl trichloroacetimidate[24] (500 mg, 1.19 mmol). The residue was purified by silica column chromatography (25% EtOAc in n-heptane) as an inseparable mixture of the spaced- and directly coupled thiophenyl glycoside (2.2/1, 330 mg). The mixture was directly used for chlorination using general procedure 2. Silica column chromatography of the residue (30% EtOAc in n-heptane) obtained **3c** (122 mg, 32% over two steps) as a clear oil. **TLC**: (EtOAc/*n*-heptane, 3/7, v/v): $R_f$ = 0.24; **$^1$H NMR** (400 MHz, $CDCl_3$) δ 5.55 (d, *J* = 6.2 Hz, 1H, -OC*H*HCl), 5.47 (d, *J* = 6.2 Hz, 1H, -OC*H*HCl), 5.21 (t, *J* = 8.3 Hz, 1H, H-3), 5.03 – 4.91 (m, 2H, H-2, H-4), 4.88 (d, *J* = 6.7 Hz, 1H, H-1), 4.15 (dd, *J* = 11.9, 5.0 Hz, 1H, H-5A), 3.46 (dd, *J* = 11.9, 8.4 Hz, 1H, H-5B), 2.08 (s, 3H, C*H*$_3$ Ac), 2.06 (s, 3H, C*H*$_3$ Ac), 2.05 (s, 3H, C*H*$_3$ Ac); **$^{13}$C NMR** (101 MHz, $CDCl_3$) δ 170.05, 170.00, 169.61, 97.01 (C-1), 77.10 (-O$CH_2$Cl), 71.00 (C-3), 69.98 (C-2), 68.75 (C-4), 62.53 (C-5), 20.88, 20.83, 20.81; **HRMS** (m/z): $[M+Na]^+$ calcd for $C_{12}H_{17}ClO_8$, 347.0510; found, 347.0528.

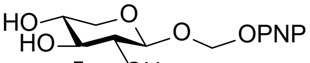

5c

**4-Nitrophenyloxymethyl β-D-xylopyranoside (5c): 5c** was synthesized in a two-step procedure. First, 4-nitrophenyloxymethyl 2,3,4-tri-*O*-acetyl-β-D-xylopyranoside (**4c**) was prepared *via* general procedure 3 starting from **3c** (40 mg, 0.12 mmol). Silicagel flash column chromatography of the residue afforded **4c** (36 mg, 68%) as a white amorphous solid (**TLC**: (EtOAc/*n*-heptane, 3/7, v/v): $R_f$ = 0.18) and was directly used for the synthesis of **5c**. **5c** was prepared v*ia* general procedure 4 using **4c** (14 mg, 0.033 mmol). The product (9.5 mg, 96%) was obtained as a white amorphous solid. **TLC**: (MeOH/DCM, 1/9, v/v): $R_f$ = 0.34; **HPLC**: Rt. 16.348; **$^1$H NMR** (500 MHz, MeOD) δ 8.26 – 8.12 (m, 2H, 2 x C*H* Ar), 7.56 – 7.06 (m, 2H, 2 x C*H* Ar), 5.56 (d, *J* = 7.2 Hz, 1H, C*H*H spacer), 5.45 (d, *J* = 7.2 Hz, 1H, C*H*H spacer), 4.55 (d, *J* = 7.5 Hz, 1H, H-1), 3.88 (dd, *J* = 11.5, 5.3 Hz, 1H, H-5A), 3.49 (ddd, *J* = 10.1, 8.6, 5.3 Hz, 1H, H-4), 3.34 – 3.28 (m, 1H, H-3), 3.27 – 3.17 (m, 2H, H-2, H-5B); **$^{13}$C NMR** (126 MHz, MeOD) δ 163.83, 143.72, 126.56, 126.50, 117.70, 117.65, 117.59, 102.13 (C-1), 91.47 (C$H_2$ spacer), 77.58 (C-3), 74.57 (C-2), 71.03 (C-4), 67.15 (C-5); **HRMS** (m/z): $[M+Na]^+$ calcd for $C_{12}H_{15}NO_8$, 304.0695; found, 304.0717.

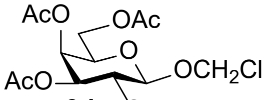

3d

**Chloromethyl 2,3,4,6-tetra-*O*-acetyl-β-D-galactopyranoside (3d):** First the phenylthiomethyl galactoside was prepared v*ia* general procedure 1 starting from 2,3,4-tri-*O*-acetyl-α-D-galactopyranosyl trichloroacetimidate[25] (500 mg, 1.01 mmol). The residue was purified by silica column chromatography (30% EtOAc in *n*-heptane) as an inseparable mixture of the spaced- and directly coupled thiophenyl glycoside (1/2.1, 403 mg). **TLC**: (EtOAc/*n*-heptane, 2/3, v/v): $R_f$ = 0.35; The mixture was directly used for chlorination using general procedure 2. Silica column chromatography of the residue obtained **3d** (86 mg, 24% over two steps) as a clear oil. **TLC**: (EtOAc/*n*-heptane, 3/7, v/v): $R_f$ = 0.19; **$^1$H NMR** (400 MHz, Chloroform-*d*) δ 5.56 (d, *J* = 6.3 Hz, 1H, -C*H*HCl), 5.49 (d, *J* = 6.3 Hz, 1H, C*H*HCl), 5.41 (dd, *J* = 3.4, 1.0 Hz, 1H, H-4), 5.23 (dd, *J* = 10.5, 8.0 Hz, 1H, H-2), 5.06 (dd, *J* = 10.5, 3.4 Hz, 1H, H-3), 4.83 (d, *J* = 8.0 Hz, 1H, H-1), 4.29 – 4.06 (m, 2H, H-6A, H-6B), 3.98 (td, *J* = 6.6, 1.1 Hz, 1H, H-5), 2.15 (s, 3H, C*H*$_3$ Ac), 2.07 (s, 3H, C*H*$_3$ Ac), 2.05 (s, 3H, C*H*$_3$ Ac), 1.98 (s, 3H, C*H*$_3$ Ac); **$^{13}$C NMR** (101 MHz, Chloroform-*d*) δ 170.47, 170.25, 170.14, 169.67, 97.37 (C-1), 77.32 (-C$H_2$Cl), 71.45 (C-5), 70.74 (C-3), 68.05 (C-2), 66.93 (C-4), 61.27 (C-6), 20.86, 20.78, 20.74, 20.66.

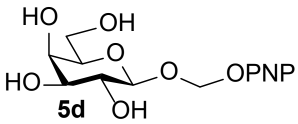

**5d**

**4-Nitrophenyloxymethyl β-D-galactopyranoside (5d):** *Via* general procedure 3 starting from **3d** (28 mg, 0.071 mmol). The crude reaction product was directly used for the synthesis of **5d** *via* general procedure 4. Silicagel flash column chromatography (0→10% MeOH in DCM) of the residue the product **5d** (22 mg, 92%) was obtained as a white amorphous solid. **TLC**: (MeOH/DCM, 1/9, v/v): $R_f$ = 0.29; **HPLC**: Rt. 14.424; **$^1$H NMR (**500 MHz, MeOD) δ 8.32 – 8.06 (m, 2H, 2 x C*H* Ar), 7.46 – 7.08 (m, 2H, 2 x C*H* Ar), 5.66 (d, *J* = 7.2 Hz, 1H, C*H*H spacer), 5.53 – 5.37 (m, 1H, C*H*H spacer), 4.56 (d, *J* = 7.8 Hz, 1H, H-1), 3.84 (dd, *J* = 3.5, 1.1 Hz, 1H, H-4), 3.74 (qd, *J* = 11.4, 6.1 Hz, 2H, H-6A, H-6B, 3.60 – 3.53 (m, 2H, H-2, H-5), 3.46 (dd, *J* = 9.7, 3.4 Hz, 1H, H-3); **$^{13}$C NMR** (126 MHz, MeOD) δ 163.94, 143.69, 126.56, 117.69, 101.58 (C-1), 91.17 ($CH_2$ spacer), 77.12 (C-5), 74.79 (C-3), 72.13 (C-2), 70.21 (C-4), 62.49 (C-6); **HRMS** (m/z): $[M+Na]^+$ calcd for $C_{13}H_{17}NO_9$, 354.0801; found, 354.0811.

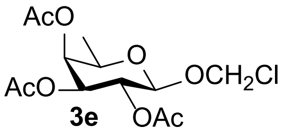

**3e**

**Chloromethyl 2,3,4-tri-*O*-acetyl-β-D-fucopyranoside (3e):** First, the phenylthiomethyl fucoside was prepared v*ia* general procedure 1 starting from 2,3,4-tri-*O*-acetyl-α-D-fucopyranosyl trichloroacetimidate[26] (500 mg, 1.15 mmol). The residue was purified by silica column chromatography (30% EtOAc in n-heptane) as an inseparable mixture of the spaced- and directly coupled thiophenyl glycoside (1/2.5, 432 mg). **TLC**: (EtOAc/*n*-heptane, 3/7, v/v): $R_f$ = 0.29; The mixture was directly used for chlorination using general procedure 2. Silica column chromatography of the residue obtained **3e** (120 mg, 31% over two steps) as a clear oil. **TLC**: (EtOAc/*n*-heptane, 3/7, v/v): $R_f$ = 0.27; **$^1$H NMR** (400 MHz, Chloroform-*d*) δ 5.60 (d, *J* = 6.2 Hz, 1H, -C*H*HCl), 5.51 (d, *J* = 6.0 Hz, 1H, -C*H*HCl), 5.27 (dd, *J* = 3.4, 0.9 Hz, 1H, H-4), 5.22 (dd, *J* = 10.5, 8.0 Hz, 1H, H-2), 5.07 (dd, *J* = 10.4, 3.4 Hz, 1H, H-3), 4.82 (d, *J* = 8.0 Hz, 1H, H-1), 3.90 (qd, *J* = 6.4, 1.0 Hz, 1H, H-5), 2.18 (s, 3H, C*H*3 Ac), 2.08 (s, 3H, C*H*3 Ac), 2.00 (s, 3H, C*H*3 Ac), 1.25 (d, *J* = 6.4 Hz, 3H, 3 x H-6). **$^{13}$C NMR** (101 MHz, Chloroform-*d*) δ 170.73, 170.29, 169.85, 97.22 (C-1), 77.42 (-$CH_2$Cl), 71.19 (C-3), 70.07 (C-4), 69.97 (C-5), 68.18 (C-2), 20.91, 20.77, 20.72, 16.08 (C-6); **HRMS** (m/z): $[M+Na]^+$ calcd for $C_{13}H_{19}ClO_8$, 361.0666; found, 361.0677.

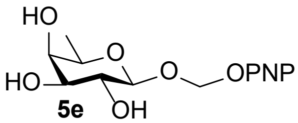

**5e**

**4-Nitrophenyloxymethyl β-D-fucopyranoside (5e)**: **5e** was synthesized in a two-step procedure. First, 4-nitrophenyloxymethyl 2,3,4-tri-*O*-acetyl-β-D-fucopyranoside (**4e**) was prepared *via* general procedure 3 starting from **3e** (50 mg, 0.15 mmol). Silicagel flash column chromatography of the residue afforded **4e** (51 mg, 78%) as a white amorphous solid (**TLC**: (EtOAc/*n*-heptane, 3/7, v/v): $R_f$ = 0.25;) and was used directly for the synthesis of **5e**. **5e** was prepared v*ia* general procedure 4 using **4e** (23 mg, 0.052 mmol). The product (15 mg, 91%) was obtained as a white amorphous solid. **TLC**: (MeOH/DCM, 1/9, v/v): $R_f$ = 0.32; **HPLC**: Rt. 17.009; **$^1$H NMR** (400 MHz, MeOD) δ 8.20 (d, *J* = 9.3 Hz, 2H, 2 x C*H* Ar), 7.52 – 6.98 (m, 2H, 2 x C*H* Ar), 5.59 (d, *J* = 7.1 Hz, 1H, C*H*H spacer), 5.46 (d, *J* = 7.1 Hz, 1H, C*H*H spacer), 4.52 (d, *J* = 7.6 Hz, 1H, H-1), 3.72 – 3.64 (m, 1H, H-5), 3.61 (dd, *J* = 3.3, 1.1 Hz, 1H, H-4), 3.52 (dd, *J* = 9.8, 7.6 Hz, 1H, H-2), 3.45 (dd, *J* = 9.7, 3.3 Hz, 1H, H-3), 1.26 (d, *J* = 6.4 Hz, 3H, 3 x H-6); $^{13}$C NMR (101 MHz, MeOD) δ 163.94, 126.53, 117.69, 101.68 (C-1), 91.30 ($CH_2$ spacer), 74.96 (C-3), 72.90 (C-4), 72.38 (C-5), 71.92 (C-2), 16.66 (C-6); **HRMS** (m/z): $[M+Na]^+$ calcd for $C_{13}H_{17}NO_8$, 338.0852; found, 338.0865.

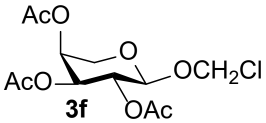

**3f**

**Chloromethyl 2,3,4-tri-*O*-acetyl-α-L-arabinopyranoside (3f):** First the phenylthiomethyl arabinoside was prepared v*ia* general procedure 1 starting from 2,3,4-tri-*O*-acetyl-β-L-arabinopyranosyl trichloroacetimidate (1.0 g, 2.38 mmol). The residue was purified by silica column chromatography (30% EtOAc in n-heptane) as an inseparable mixture of the spaced- and directly coupled thiophenyl glycoside (1/1.6, 730 mg). **TLC**: (EtOAc/*n*-heptane, 3/7, v/v): $R_f$ = 0.21; The mixture was directly used for chlorination using general procedure 2. Silica column chromatography (30% EtOAc in n-heptane) of the residue obtained **3f** (270 mg, 35% over two steps) as a clear oil. **TLC**: (EtOAc/*n*-heptane, 2/3, v/v): $R_f$ = 0.39; **$^1$H NMR** (400 MHz, $CDCl_3$) δ 5.58 (d, *J* = 6.2 Hz, 1H, -OC*H*HCl), 5.49 (d, *J* = 6.2 Hz, 1H, -OC*H*HCl), 5.29 (td, *J* = 3.5, 1.8 Hz, 1H, H-4), 5.22 (dd, *J* = 9.2, 6.9 Hz, 1H, H-2), 5.09 (ddd, *J* = 9.3, 3.5, 1.6 Hz, 1H, H-3), 4.80 (d, *J* = 6.9 Hz, 1H, H-1), 4.05 (dd, *J* = 13.0, 3.4 Hz, 1H, H-5A), 3.70 (dd, *J* = 12.9, 1.9 Hz, 1H, H-5B), 2.13 (s, 3H, C*H*3 Ac), 2.09 (s, 3H, C*H*3 Ac), 2.03 (s, 3H, C*H*3 Ac); **$^{13}$C NMR** (126 MHz, $CDCl_3$) δ 170.34, 170.16, 169.68, 97.19 (C-1), 77.16 (-O$CH_2$Cl), 69.93 (C-3), 68.46 (C-2), 67.39 (C-4), 63.70 (C-5), 21.01, 20.90, 20.78; **HRMS** (m/z): $[M+Na]^+$ calcd for $C_{12}H_{17}ClO_8$, 347.0510; found, 347.0529.

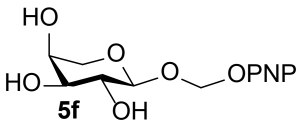

5f

**4-Nitrophenyloxymethyl α-L-arabinopyranoside (5f): 5f** was synthesized in a two-step procedure. First, 4-nitrophenyloxymethyl 2,3,4-tri-*O*-acetyl-α-L-arabinopyranoside (**4f**) was prepared *via* general procedure 3 starting from **3f** (50 mg, 0.15 mmol). Silicagel flash column chromatography (40% EtOAc in n-heptane) of the residue afforded **4f** (48 mg, 73%) as a white amorphous solid. (**TLC**: (EtOAc/*n*-heptane, 2/3, v/v): $R_f$ = 0.29;) and was used directly for the synthesis of **5f** *via* general procedure 4 using **4f** (17 mg, 0.040 mmol). The product (10.4 mg, 87%) was obtained as a white amorphous solid. **TLC**: (MeOH/DCM, 1/9, v/v): $R_f$ = 0.36; **HPLC**: Rt. 15.853; **$^1$H NMR** (500 MHz, MeOD) δ 8.34 – 8.02 (m, 2H, 2 x C*H* Ar), 7.34 – 7.22 (m, 2H, 2 x C*H* Ar), 5.60 (d, *J* = 7.2 Hz, 1H, C*H*H spacer), 5.48 (d, *J* = 7.3 Hz, 1H, C*H*H spacer), 4.57 (d, *J* = 6.7 Hz, 1H, H-1), 3.91 (dd, *J* = 12.4, 3.3 Hz, 1H, H-5A), 3.85 (td, *J* = 3.3, 1.7 Hz, 1H, H-4), 3.66 – 3.53 (m, 3H, H-2, H-3, H-5B); **$^{13}$C NMR** (126 MHz, MeOD) δ 163.87, 143.68, 126.56, 117.63, 101.79 (C-1), 91.24 (*C*$H_2$ spacer), 73.96 (C-3), 72.11 (C-2), 69.33 (C-4), 66.98 (C-5); **HRMS** (m/z): $[M+Na]^+$ calcd for $C_{12}H_{15}NO_8$, 324.0695; found, 324.0716..

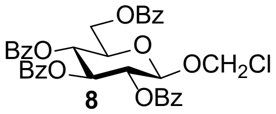

8

**Chloromethyl 2,3,4,6-tetra-*O*-benzoyl-β-D-glucopyranoside** (**8**): Based on a procedure by Crich and coworkers.[16] A solution of 2,3,4,6-tetra-*O*-benzoyl-α/β-D-glucopyranosyl trichloroacetimidate (1.0 g, 1.5 mmol), phenylthiomethanol (378 mg, 321 µL, 2,70 mmol, 2 eq) and freshly activated 4 Å molecular sieves in dry DCM (13 mL) was stirred for 20 min at rt and then cooled to 0 °C. A catalytic amount of AgOTf (26 mg, 0.1 mmol, 0.075 eq) was added and the reaction mixture stirred at 0 °C for 60 min. The reaction was then quenched by the addition of sat. aq. $NaHCO_3$. The mixture was filtered through Celite and the organic layer, washed with brine and concentrated *in vacuo*. The resulting oil was purified by silica gel column chromatography (EtOAc/*n*-heptane, 0 to 25%, v/v), affording phenylthiomethyl 2,3,4,6-tetra-O-benzoyl-β-D-glucopyranoside[27] (788 mg, 81%) as a yellow foam with the direct thioether as an impurity (**TLC** (EtOAc/*n*-heptane, 2/3, v/v): $R_f$ = 0.45). The mixture was used directly in the synthesis of **8**. To a stirring solution of phenylthiomethyl 2,3,4,6-tetra-O-benzoyl-β-D-glucopyranoside (1.4 g, 1.9 mmol) in dry DCM (19 mL) was added dropwise at 0°C a solution of sulfuryl dichloride (0.34 g, 0.20 mL, 2.5 mmol) in dry DCM (9 mL). The mixture was stirred under nitrogen for 30 min at 0 °C and for 15 min at rt before the mixture was concentrated *in vacuo*. The resulting crude oil was purified by silica gel column chromatography (EtOAc/*n*-heptane, 0 to 25%, v/v) to afford chloromethyl 2,3,4,6-tetra-O-benzoyl-β-D-glucopyranoside (73%) as a white amorphous solid. **TLC** (EtOAc/*n*-heptane, 2/3, v/v): $R_f$ = 0.5; **$^1$H NMR** (400 MHz, $CDCl_3$) δ 8.04 (dq, *J* = 6.9, 1.7 Hz, 4H), 7.97 (dt, *J* = 8.4, 1.4 Hz, 4H), 7.90 (ddd, *J* = 8.6, 2.3, 1.3 Hz, 3H), 7.83 (dt, *J* = 8.4, 1.1 Hz, 3H), 7.59 – 7.26 (m, 20H), 5.95 (t, *J* = 9.6 Hz, 1H, H-3), 5.72 (t, *J* = 9.7 Hz, 2H, H-4), 5.64 (d, *J* = 6.3 Hz, 1H, -OC*H*HCl), 5.59 (dd, *J* = 9.7, 8.0 Hz, 1H, H-2), 5.50 (d, *J* = 6.3 Hz, 1H, -OC*H*HCl), 5.27 (d, *J* = 8.0 Hz, 1H, H-1), 4.67 (dd, *J* = 12.2, 3.0 Hz, 1H, H-6A), 4.51 (dd, *J* = 12.2, 5.2 Hz, 2H, H-6B), 4.25 (ddd, *J* = 9.9, 5.1, 3.0 Hz, 1H, H-5); **$^{13}$C NMR** (101 MHz, $CDCl_3$) δ 166.10, 165.72, 165.18, 165.15, 133.54, 133.36, 133.32, 133.24, 129.91, 129.85, 129.78, 129.76, 129.48, 129.02, 128.69, 128.65, 128.46, 128.44, 128.38, 128.33, 96.87 (C-1), 77.09 (-O*C*$H_2$Cl), 72.85 (C-5), 72.73 (C-3), 71.10 (C-2), 69.34 (C-4), 62.74 (C-6); **HRMS** (m/z): $[M+Na]^+$ calcd for $C_{35}H_{29}ClO_{10}$, 667.1347; found, 667.1350.

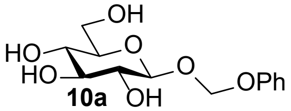

10a

**Phenyloxymethyl β-D-glucopyranoside (10a): 10a** was synthesized in a two-step procedure. First, phenyloxymethyl 2,3,4,6-tetra-*O*-benzoyl-β-D-glucopyranoside (**9a**) was prepared v*ia* general synthetic procedure 2 starting from **8** (100 mg, 0.155 mmol). Silica column chromatography (30% EtOAc in *n*-heptane) of the residue gave the product (70 mg, 64%) as a white solid (**TLC** (EtOAc/*n*-heptane, 2/3, v/v): $R_f$ = 0.43) and was directly used for the synthesis of **10a** *via* general synthetic procedure 2 starting from **9a** (70 mg). Silica column chromatography (10% DCM in MeOH) of the residue gave **10a** (24 mg, 85%) as a white solid. **TLC** (MeOH/DCM, 1/9, v/v): $R_f$ = 0.18; **HPLC**: Rt. 11.027; **$^1$H NMR** (500 MHz, MeOD) δ 7.33 – 7.24 (m, 2H, 2 x C*H* Ar), 7.15 – 7.07 (m, 2H, 2 x C*H* Ar), 6.99 (tt, *J* = 7.4, 1.1 Hz, 1H, 1 x C*H* Ar), 5.54 (d, *J* = 6.9 Hz, 1H, C*H*H spacer), 5.32 (d, *J* = 6.9 Hz, 1H, C*H*H spacer), 4.63 (d, *J* = 7.9 Hz, 1H, H-1), 3.90 – 3.82 (m, 1H, H-6A), 3.77 – 3.59 (m, 1H, H-6B), 3.39 – 3.28 (m, 3H, H-3, H-4, H5), 3.23 (dd, *J* = 9.1, 7.9 Hz, 1H, H-2); **$^{13}$C NMR** (126 MHz, MeOD) δ 159.05, 130.41, 123.14, 117.73, 100.61 (C-1), 91.64 (*C*$H_2$ spacer), 78.24 (C-4), 77.94 (C-3), 74.90 (C-2), 71.56 (C-5), 62.73 (C-6); **HRMS** (m/z): $[M+Na]^+$ calcd for $C_{13}H_{18}O_7$, 309.0950; found, 309.0953.

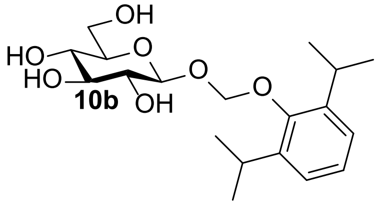


**2,6-Diisopropylphenyloxymethyl β-D-glucopyranoside (10b): 10b** was synthesized in a two-step procedure. First, 2,6-diisopropylphenyloxymethyl 2,3,4,6-tetra-*O*-benzoyl-β-D-glucopyranoside (**9b**) was prepared *via* general synthetic procedure 1 starting from **8** (425 mg, 0.659 mmol) and propofol. Silica gel column chromatography (EtOAc/*n*-heptane, 0 to 25%, v/v) afforded **9c** (400 mg, 77%) as a yellow oil (**TLC** (EtOAc/*n*-heptane, 2/3, v/v): $R_f$ = 0.5) and was used directly for the synthesis of **10b** v*ia* general synthetic procedure 3 starting from 400 mg **9b**. Silica column chromatography (MeOH/DCM, 0 to 20%, v/v) of the residue, afforded the product **10b** (120 mg, 70%) as a white solid. **TLC** (MeOH/DCM, 1/4, v/v): $R_f$ = 0.5; **HPLC**: Rt. 15.050 min; **$^1$H NMR** (500 MHz, $CD_3OD$) δ 7.15 – 7.08 (m, 3H, 3 x C*H* Ar), 5.37 (d, *J* = 5.2 Hz, 1H, C*H*H spacer), 5.03 (d, *J* = 5.2 Hz, 1H, C*H*H spacer), 4.75 (d, *J* = 7.8 Hz, 1H, H-1), 3.90 (dd, *J* = 12.0, 2.3 Hz, 1H, H-6A), 3.73 (dd, *J* = 12.1, 5.3 Hz, 1H, H-6B), 3.50 – 3.43 (m, 3H, H-3 + C*H* propofol), 3.38 (t, *J* = 10.8 Hz, 1H, H-4), 3.35 – 3.30 (m, 2H, H-2, H-5), 1.22 (d, *J* = 7.0 Hz, 12H, 4 x $CH_3$ propofol); **$^{13}$C NMR** (126 MHz, MeOD) δ 152.15, 143.08, 126.25, 124.93, 101.95 (C-1), 96.98 ($CH_2$ spacer), 78.17 (H-5), 77.91 (C-3), 74.87 (C-2), 71.31 (C-4), 62.56 (C-6), 27.62, 24.33; **HRMS** (m/z): $[M+Na]^+$ calcd for $C_{47}H_{46}O_{11}$, 393.1889; found, 393.1890.

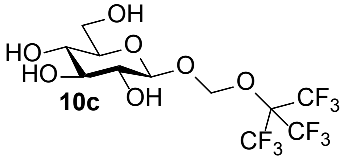


**Perfluoro-*tert*-butyloxymethyl β-D-glucopyranoside (10c): 10c** was synthesized in a two-step procedure. First, perfluoro-*tert*-butyloxymethyl 2,3,4,6-tetra-*O*-benzoyl-β-D-glucopyranoside (**9c**) was prepared *via* general synthetic procedure 1 starting from **5** (100 mg, 0.155 mmol). Silicagel flash column chromatography of the residue (EtOAc (0→30%) in *n*-heptane) obtained the product **9c** (62 mg, 47%) as white amorphous solid. (**TLC** (EtOAc/*n*-heptane, 3/7, v/v)**:** $R_f$ = 0.43) and was used directly in the preparation of **10c** *via* general synthetic procedure 2 starting from **9c** (60 mg, 0.071 mmol). Silica column (MeOH/DCM, 0 to 10%, v/v), affording **10c** (17 mg, 56%) as a white solid. **TLC** (MeOH/DCM, 1/9, v/v): $R_f$ = 0.19; **$^1$H NMR** (500 MHz, $D_2O$) δ 5.60 (d, *J* = 6.2 Hz, 1H, C*H*H spacer), 5.45 (d, *J* = 6.2 Hz, 1H, C*H*H spacer), 4.79 (s, 428H, solvent peak, H-1 as determined from $^1$H$^{13}$C-HSQC), 3.92 (dd, *J* = 12.4, 2.2 Hz, 1H, H-6A), 3.76 (dd, *J* = 12.4, 5.2 Hz, 1H, H-6B), 3.61 – 3.42 (m, 3H, H-3, H-4, H-5), 3.37 (ddd, *J* = 9.1, 7.9, 0.8 Hz, 1H, H-2); **$^{13}$C NMR** (126 MHz, $D_2O$) δ121.04, 118.71, 100.18 (C-1), 91.57 (*C*$H_2$ spacer), 76.18, 75.48, 72.67, 69.33, 60.43 (C-6); **$^{19}$F NMR** (471 MHz, $D_2O$) δ -70.72; **HRMS** (m/z): $[M+Na]^+$ calcd for $C_{11}H_{13}F_9O_7$, 451.0415; found, 415.0424.

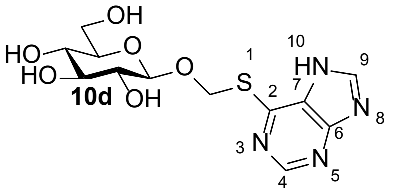


**6-Thiopurinyloxymethyl β-D-glucopyranoside (10d): 10d** was synthesized in a two-step procedure. First, 6-thiopurinyloxymethyl 2,3,4,6-tetra-*O*-benzoyl-β-D-glucopyranoside (**9d**) was prepared *via* general procedure 1 starting from **8** (200 mg, 0.31 mmol) using 6-mercaptopurine as coupling substrate. Silicagel column chromatography (MeOH/DCM, 0 to 10%, v/v) of the residue afforded **9d** (114 mg, 48%) as a pale oil (**TLC** (MeOH/DCM, 1/9, v/v): $R_f$ = 0.49) and was used directly in the synthesis of **10d** v*ia* general synthetic procedure 2 starting from **9d** (110 mg, 0.145 mmol). Silica column chromatography ($H_2O$ in ACN, 1/9, v/v) of the residue gave **10d** (21 mg, 42%) as a white amorphous solid. **TLC** ($H_2O$/ACN, 1/9, v/v): $R_f$ = 0.26; **$^1$H NMR** (400 MHz, $D_2O$) δ 8.75 (s, 1H, H-4'), 8.45 (s, 1H, H-9'), 5.89 (d, *J* = 11.9 Hz, 1H, C*H*H spacer), 5.78 (d, *J* = 11.9 Hz, 1H, C*H*H spacer), 3.90 – 3.56 (m, 2H, H-6A, H-6B), 3.56 – 3.10 (m, 4H, H-2, H-3, H-4, H-5); **$^{13}$C NMR** (101 MHz, $D_2O$) δ 100.53 (C-1), 76.12 (C-3), 75.57 (C-5), 72.68 (C-2), 69.37 (C-4), 67.87 (*C*$H_2$ linker), 60.44 (C-5); **HRMS** (m/z): $[M+Na]^+$ calcd for $C_{12}H_{16}N_4O_6S$, 367.0688; found, 367.0689.

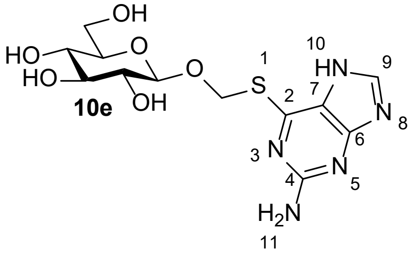


**6-Thioguanyloxymethyl β-D-glucopyranoside (10e): 10e** was synthesized in a two-step procedure. First, 6-thioguanyloxymethyl 2,3,4,6-tetra-*O*-benzoyl-β-D-glucopyranoside (**9e**) was prepared *via* general procedure 1 starting from **8** (400 mg, 0.62 mmol) using tioguanine as coupling substrate. Silicagel column chromatography (MeOH/DCM, 0 to 10%, v/v) of the residue afforded **9e** (246 mg, 51%) as a pale oil (**TLC** (MeOH/DCM, 1/9, v/v): $R_f$ = 0.4) and was used directly in the synthesis of **10e** *via* general procedure 2 starting from **9e** (70 mg, 0.090 mmol). Silicagel column chromatography ($H_2O$ in

ACN, 1/4, v/v) of the residue gave **10e** (30 mg, 93%) as a white amorphous solid. **TLC** ($H_2O$/ACN, 4/1, v/v): $R_f$ = 0.2; **$^1$H NMR** (400 MHz, $D_2O$) δ 8.01 (s, 1H, H-9'), 5.75 (ad, *J* = 2.6 Hz, 2H, 2 x C*H*H spacer), 4.75 (d, *J* = 8.0 Hz, 1H, H-1), 3.73 – 3.61 (m, 2H, H-6A, H-6B), 3.46 (t, *J* = 8.9 Hz, 1H, H-3), 3.41 – 3.25 (m, 3H, H-2, H-4, H-5); **$^{13}$C NMR** (101 MHz, $D_2O$) δ 159.30 (C-2'), 100.66 (C-1), 76.14 (C-5), 75.63 (C-3), 72.71 (C-2), 69.49 (C-4), 67.40 (*C*$H_2$ spacer), 60.55 (C-6); **HRMS** (m/z): $[M+Na]^+$ calcd for $C_{12}H_{17}N_5O_6S$, 382.0797; found, 382.0817

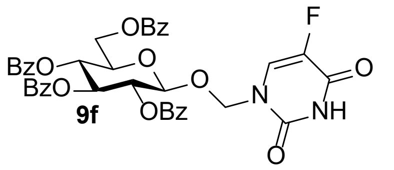

**1-(2,3,4,6-tetra-*O*-benzoyl-β-D-glucopyranosyloxymethyl)-5-fluorouracil (9f):** *Via* general synthetic procedure 1 starting from **8** (200 mg, 0.62 mmol) and 5-fluorouracil. 2.5 equivalents of NaH was used. Silicagel column chromatography (2→10% MeOH in DCM) of the residue afforded **9f** (71 mg, 31%) and **9g** (50 mg, 19%) both as white amorphous solids. Characterization of **9f**: **TLC** (EtOAc/*n*-heptane, 3/2, v/v): $R_f$ = 0.50; **$^1$H NMR** (500 MHz, Acetone) δ 10.36 (d, *J* = 5.0 Hz, 1H, -NH), 8.05 – 7.91 (m, 2H, 2 x C*H* Ar), 7.79 (dq, *J* = 6.7, 1.5 Hz, 4H, 4 x C*H* Ar), 7.74 – 7.63 (m, 3H, 2 x C*H* Ar, C*H* 5FU), 7.55 – 7.48 (m, 1H, C*H* Ar), 7.48 – 7.42 (m, 2H, 2 x C*H* Ar), 7.42 – 7.34 (m, 3H, 3 x C*H* Ar), 7.30 (tdd, *J* = 7.4, 5.6, 1.7 Hz, 4H, 4 x C*H* Ar), 7.26 – 7.21 (m, 2H, 2 x C*H* Ar), 5.90 (ddd, *J* = 9.5, 8.2, 1.4 Hz, 1H, H-3), 5.66 (t, *J* = 9.7 Hz, 1H, H-4), 5.50 (dd, *J* = 11.0, 0.7 Hz, 1H, C*H*H spacer), 5.43 – 5.34 (m, 2H, H-1, H-2), 5.17 (d, *J* = 11.0 Hz, 1H, C*H*H spacer), 4.57 (dd, *J* = 12.1, 2.7 Hz, 1H, H-6a), 4.48 – 4.32 (m, 2H, H6b, H-5); **$^{13}$C NMR** (126 MHz, Acetone) δ 165.51, 165.22, 164.90, 164.72, 156.84, 156.71, 156.63, 149.84, 149.77, 141.41, 139.55, 133.50, 133.38, 133.15, 129.92, 129.56, 129.37, 129.24, 129.20, 129.15, 128.56, 128.51, 128.50, 128.46, 128.01, 127.74, 99.62 (C-1), 75.41 (*C*$H_2$ spacer), 73.25 (C-3), 72.27 (C-5), 71.77 (C-2), 69.37 (C-4), 66.71, 62.50 (C-6). **HRMS** (m/z): $[M+Na]^+$ calcd for $C_{39}H_{31}FN_2O_{12}$, 761.1759; found, 761.1749.

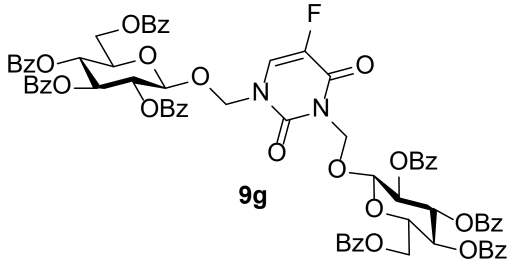

**1,3-bis-(2,3,4,6-tetra-*O*-benzoyl-β-D-glucopyranosyloxymethyl)-5-fluorouracil (9g): TLC** (EtOAc/*n*-heptane, 3/2, v/v): $R_f$ = 0.67; **$^1$H NMR** (500 MHz, Chloroform-*d*) δ 8.03 (tt, *J* = 6.6, 1.4 Hz, 5H), 7.89 (dddd, *J* = 9.9, 8.7, 7.0, 1.4 Hz, 10H), 7.81 (ddd, *J* = 8.4, 2.9, 1.3 Hz, 5H, C*H* Ar), 7.62 – 7.46 (m, 8H), 7.46 – 7.38 (m, 8H), 7.38 – 7.31 (m, 10H), 7.30 – 7.24 (m, 6H,), 6.97 (d, *J* = 4.9 Hz, 1H, C*H* 5FU), 5.88 (td, *J* = 9.7, 0.9 Hz, 2H, 2 x H-3), 5.70 (td, *J* = 9.8, 8.2 Hz, 2H, 2 x H-4), 5.59 (d, *J* = 10.6 Hz, 1H, C*H*H linker), 5.53 (d, *J* = 10.8 Hz, 1H, C*H*H linker), 5.46 (ddd, *J* = 12.4, 9.8, 8.0 Hz, 2H, 2 x H-2), 5.25 (d, *J* = 8.1 Hz, 1H, H-1), 5.18 (q, *J* = 10.4, 7.8 Hz, 1H, C*H*H linker), 5.08 (d, *J* = 7.9 Hz, 1H, H-1), 4.99 (d, *J* = 11.1 Hz, 1H, C*H*H linker), 4.70 (dd, *J* = 12.3, 3.0 Hz, 1H, H-6A)), 4.65 (dd, *J* = 12.3, 3.0 Hz, 1H, H-6A), 4.33 (dt, *J* = 12.3, 3.4 Hz, 2H, 2 x H-6B), 4.11 (dq, *J* = 11.1, 3.4, 2.8 Hz, 2H, 2 x H-5); **$^{13}$C NMR** (126 MHz, Chloroform-*d*) δ 166.17, 166.06, 165.86, 165.79, 165.18, 165.16, 165.08, 164.77, 157.05, 156.84, 149.85, 140.85, 138.95, 133.79, 133.67, 133.56, 133.46, 133.40, 133.35, 133.32, 130.05, 129.95, 129.92, 129.90, 129.88, 129.81, 129.77, 129.64, 129.50, 129.42, 128.92, 128.90, 128.84, 128.78, 128.76, 128.64, 128.61, 128.59, 128.57, 128.54, 128.50, 128.45, 128.41, 126.08, 125.82, 102.06, 101.21, 76.79, 72.96, 72.88, 72.62, 72.57, 71.87, 71.69, 70.90, 69.21, 69.06, 62.30, 62.03; **HRMS** (m/z): $[M+Na]^+$ calcd for $C_{74}H_{59}FN_2O_{22}$, 1369.3441; found, 1369.3408

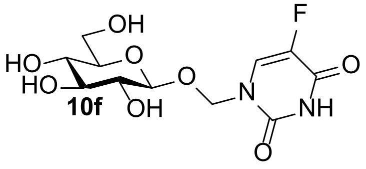

**1-(β-D-glucopyranosyloxymethyl)-5-fluorouracil** (**10f**): *Via* general synthetic procedure 2 staring from **9f** (21 mg, 0.028 mmol). The residue was purified by silicagel column chromatography (5→10% $H_2O$ in ACN) and afforded **16g** (9 mg, 98%) as clear solid. **TLC** ($H_2O$/ACN, 1/9, v/v): $R_f$ = 0.40; **$^1$H NMR** (500 MHz, $D_2O$) δ 7.88 (d, *J* = 5.8 Hz, 1H, C*H* uracil), 5.44 (d, *J* = 11.3 Hz, 1H, C*H*H spacer), 5.28 (d, *J* = 11.2 Hz, 1H, C*H*H spacer), 4.59 (d, *J* = 7.9 Hz, 1H, H-1), 3.77 (dd, *J* = 12.4, 2.2 Hz, 1H, H-6A), 3.65 (dd, *J* = 12.4, 5.1 Hz, 1H, H-6B), 3.50 – 3.29 (m, 3H, H-3, H-4, H-5), 3.24 (dd, *J* = 9.3, 7.9 Hz, 1H, H-2); **$^{13}$C NMR** (126 MHz, $D_2O$) δ 159.91, 150.92, 140.31 (d, *J* = 273.7 Hz), 129.93 (d, *J* = 33.5 Hz), 102.09 (C-1), 77.08, 76.07, 75.48, 72.73, 69.23, 60.35; **$^{19}$F NMR** (471 MHz, $D_2O$) δ -167.16. **HRMS** (m/z): $[M+Na]^+$ calcd for $C_{11}H_{15}FN_2O_8$, 345.0710; found, 345.0729.

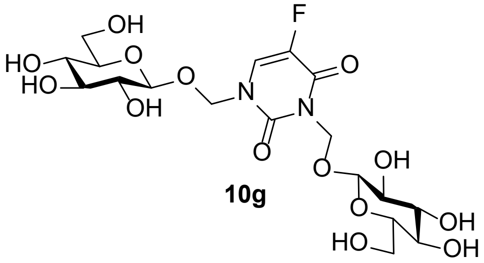

10g

**1,3-bis-(β-D-glucopyranosyloxymethyl)-5-fluorouracil (10g):** *Via* general synthetic procedure 2 staring from **9g** (33 mg). The residue was purified by silicagel column chromatography (5→10% $H_2O$ in ACN) and afforded **10g** (10 mg, 79%) as clear solid. **TLC** ($H_2O$/ACN, 1/4, v/v): $R_f$ = 0.39; **$^1$H NMR** (500 MHz, $D_2O$) δ 8.03 (d, *J* = 5.5 Hz, 1H, C*H*, 5FU), 5.70 (d, *J* = 10.9 Hz, 1H, C*H*H spacer), 5.66 (d, *J* = 11.1 Hz, 1H, C*H*H spacer), 5.60 (d, *J* = 11.3 Hz, 1H, C*H*H spacer), 5.38 (d, *J* = 11.3 Hz, 1H, C*H*H spacer), 4.72 (d, *J* = 7.9 Hz, 1H, H-1), 4.69 (d, *J* = 7.9 Hz, 1H, H-1), 3.97 – 3.76 (m, 2H, 2 x H-6A), 3.75 – 3.61 (m, 2H, 2 x H-6B), 3.55 – 3.24 (m, 8H, 2 x H-2, 2 x H-3, 2 x H-4, 2 x H-5); **$^{13}$C NMR** (126 MHz, $D_2O$) δ 159.05 (d, *J* = 25.6 Hz, -N(*C*=O)CF-), 150.75 (-N(*C*=O)N-), 129.30 (d, *J* = 34.0 Hz), 102.89 (C-1), 102.23 (C-1), 78.13, 76.13, 76.08, 75.60, 75.48, 72.77, 72.74, 70.92, 69.28, 69.26, 60.39, 60.35; **HRMS** (m/z): $[M+Na]^+$ calcd for $C_{18}H_{27}FN_2O_{14}$, 537.1344; found, 537.1347.

**(2,3,4,6-tetra-*O*-benzoyl-β-D-glucopyranosyloxymethyl)-losartan conjugates:** *Via* general synthetic procedure 1 starting from **8** (1.5 gram, 2.33 mmol) using DIPEA (2.5 equivalent) instead of NaH. The residue was purified by silica column chromatography (EtOAc/*n*-heptane, 1/1, v/v) to obtain **9i** (600 mg, 25%) and **9h** (1.3 g, 54 %) as white amorphous solids.

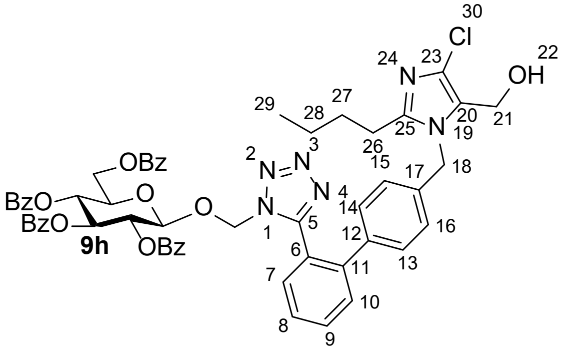

**1,5 regioisomer (9h): TLC** (EtOAc/*n*-heptane, 1/1, v/v): $R_f$ = 0.21; **$^1$H NMR** (400 MHz, $CDCl_3$) δ 8.05 (dd, *J* = 8.4, 1.3 Hz, 2H, 2 x C*H* Ar), 7.87 (dd, *J* = 8.4, 1.2 Hz, 2H, 2 x C*H* Ar), 7.82 – 7.76 (m, 4H, 4 x C*H* Ar), 7.61 – 7.21 (m, 15H, 15 x C*H* Ar), 7.16 – 7.07 (m, 2H, 2 x C*H* Ar), 7.03 (d, *J* = 8.3 Hz, 2H, 2 x C*H* Ar), 6.87 (d, *J* = 8.3 Hz, 2H, 2 x C*H* Ar), 5.77 (t, *J* = 9.6 Hz, 1H, H-3), 5.64 (t, *J* = 9.7 Hz, 1H, H-4), 5.48 (d, *J* = 11.6 Hz, 1H, C*H*H spacer), 5.35 (dd, *J* = 9.7, 7.9 Hz, 1H, H-2), 5.21 – 5.03 (m, 3H, C*H*H spacer, 2 x H-18'), 4.92 (d, *J* = 7.9 Hz, 1H, H-1), 4.57 (dd, *J* = 12.3, 3.0 Hz, 1H, H-6a), 4.46 (s, 2H, 2 x H-21'), 4.36 (dd, *J* = 12.3, 4.3 Hz, 1H, H-6b), 4.07 (dt, *J* = 9.8, 4.1 Hz, 1H, H-5), 2.59 – 2.43 (m, 2H, 2 x H-26'), 1.71 – 1.51 (m, 2H, 2 x H-27'), 1.36 – 1.19 (m, 2H, 2 x H-28'), 0.96 – 0.75 (m, 3H, 3 x H-29'); **$^{13}$C NMR** (101 MHz, $CDCl_3$) δ 166.21, 165.70, 165.20, 164.96, 155.22 (C-5'), 148.69 (C-25'), 140.93 (C-11'), 138.50 (C-12'), 136.36 (C-17'), 133.84, 133.72, 133.48, 133.46, 131.94 (C-9'), 131.33 (C-8'), 130.51 (C-10'), 129.98, 129.92, 129.87, 129.55, 129.36 (C-13', C-14'), 128.70, 128.62, 128.60, 128.46 (C-23'), 128.17 (C-8'), 127.94, 126.60 (C-15', C-16'), 124.76 (C-20'), 121.62 (C-6'), 98.25 (C-1), 72.89 (C-5), 72.61 (C-3), 71.95 ($CH_2$ spacer), 71.21 (C-2), 69.02 (C-4), 62.44 (C-6), 53.35 (C-21'), 47.21 (C-18'), 29.86 (C-27'), 26.90 (C-26'), 22.53 (C-28'), 13.88 (C-29'); **HRMS** (m/z): $[M+H]^+$ calcd for $C_{57}H_{51}ClN_6O_{11}$, 1031.3383; found, 1031.3394.

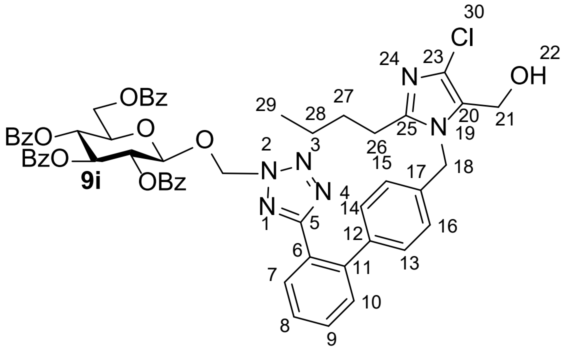

**2,5 regioisomer (9i): TLC** (EtOAc/*n*-heptane, 1/1, v/v): $R_f$ = 0.32; **$^1$H NMR** (400 MHz, $CDCl_3$) δ 8.03 (dd, *J* = 8.4, 1.3 Hz, 2H, 2 x C*H* Ar), 7.89 (dd, *J* = 8.4, 1.3 Hz, 1H, ), 7.80 (dd, *J* = 8.4, 1.3 Hz, 2H, 2 x C*H* Ar), 7.77 – 7.70 (m, 3H, 3 x C*H* Ar), 7.59 – 7.47 (m, 3H, 3 x C*H* Ar), 7.47 – 7.24 (m, 10H, 10 x C*H* Ar), 7.19 (t, *J* = 7.8 Hz, 2H, 2 x C*H* Ar), 7.01 (d, *J* = 8.3 Hz, 2H, 2 x C*H* Ar), 6.94 (d, *J* = 8.4 Hz, 2H, 2 x C*H* Ar), 6.07 (d, *J* = 11.3 Hz, 1H, C*H*H spacer), 5.91 (d, *J* = 11.2 Hz, 1H, C*H*H spacer), 5.83 (t, *J* = 9.6 Hz, 1H, H-3), 5.70 (t, *J* = 9.7 Hz, 1H, H-4), 5.53 (dd, *J* = 9.7, 7.9 Hz, 1H, H-2), 5.20 (d, *J* = 2.0 Hz, 2H, 2 x H-18'), 5.08 (d, *J* = 7.9 Hz, 1H, H-1), 4.63 (dd, *J* = 12.3, 3.0 Hz, 1H, H-6a), 4.58 – 4.39 (m, 3H, 2 x H-21', H-6b), 4.19 (ddd, *J* = 9.9, 4.8, 3.1 Hz, 1H, H-5), 2.65 – 2.56 (m, 2H, 2 x H-26'), 1.74 – 1.49 (m, 2H, 2 x H-27'), 1.43 – 1.08 (m, 2H, 2 x H-28'), 0.87 (t, *J* = 7.4 Hz, 3H, 3 x H-29'); **$^{13}$C NMR** (101 MHz, $CDCl_3$) δ 166.29, 166.06, 165.91, 165.24, 165.14, 148.65 (C-25'), 141.29 (C-11'), 140.82 (C-12'), 135.20 (C-17'), 133.71, 133.53 (C-5'), 133.39, 130.97 (C-10'), 130.56 (C-

9'), 130.40 (C-8'), 129.96, 129.94 (C-13', C-14'), 129.91, 129.77, 129.56, 128.80, 128.75, 128.70, 128.61, 128.57, 128.48 (C-23'), 127.94 (C-7'), 126.10 (C-15', C-16'), 125.50 (C-6'), 124.87 (C-20'), 98.90 (C-1), 77.36 (C$H_2$ spacer), 72.91 (C-5), 72.74 (C-3), 71.37 (C-2), 69.28 (C-4), 62.81 (C-6), 53.36 (C-21'), 47.60 (C-18'), 29.98 (C-27'), 27.01 (C-26'), 22.58 (C-28'), 13.90 (C-29'); **HRMS** (m/z): $[M+H]^+$ calcd for $C_{57}H_{51}ClN_6O_{11}$, 1031.3383; found, 1031.3385.

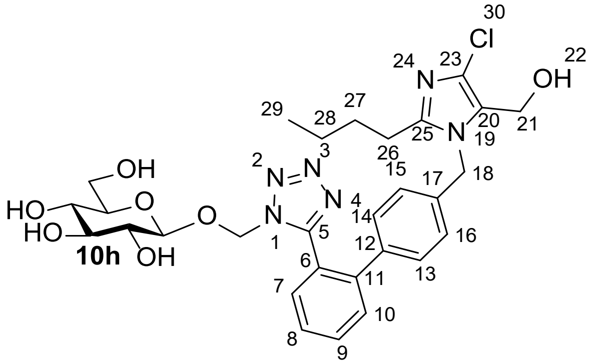

**10h**

**1*H*-(β-D-glucopyranosyl oxymethyl)-Losartan (10h):** *Via* general synthetic procedure 2 starting from **9h** (800 mg, 0.776 mmol). Silicagel column chromatography (12 % MeOH in DCM) of the residue afforded **10h** (248 mg, 51%) as a white amorphous solid. **TLC** (MeOH/DCM, 1/9, v/v) $R_f$ = 0.15; **HPLC**: Rt. 12.966 min; **$^1$H NMR** (500 MHz, MeOD) δ 7.74 (td, *J* = 7.0, 1.3 Hz, 2H, 2 x C*H* Ar), 7.61 (dd, *J* = 8.2, 7.1 Hz, 2H, 2 x C*H* Ar), 7.21 – 7.11 (m, 2H, 2 x C*H* Ar), 7.03 (d, *J* = 8.1 Hz, 2H, 2 x C*H* Ar), 5.65 (d, *J* = 11.7 Hz, 1H, C*H*H spacer), 5.51 (d, *J* = 11.7 Hz, 1H C*H*H spacer), 5.29 (s, 1H, 2 x H-18'), 4.45 (s, 2H, 2 x H-21'), 4.23 (d, *J* = 7.8 Hz, 1H, H-1), 3.71 (dd, *J* = 12.0, 2.4 Hz, 1H, H-6a), 3.58 (dd, *J* = 11.9, 5.4 Hz, 1H, H-6b), 3.27 – 3.17 (m, 2H, H-3, H-4), 3.07 (atdd, *J* = 8.3, 4.3, 1.7 Hz, 2H, H-2, H-5), 2.58 – 2.51 (m, 2H, 2 x H-26'), 1.61 – 1.45 (m, 2H, 2 x H-27'), 1.30 (h, *J* = 7.4 Hz, 2H, 2 x H-28'), 0.87 (t, *J* = 7.4 Hz, 3H, 3 x H-29'); **$^{13}$C NMR** (126 MHz, MeOD) δ 156.62, 150.07, 143.25, 140.03, 137.89, 133.10, 132.66, 131.75, 130.52, 129.27, 127.71, 127.46, 126.91, 123.16, 102.55 (C-1), 78.20, 77.82, 74.43, 74.34, 71.11, 68.14, 62.39, 53.04, 48.16, 30.91, 27.40, 23.25, 14.03. **HRMS** (m/z): $[M+Na]^+$ calcd for $C_{31}H_{31}ClN_6O_7$, 637.2153; found, 637.2181.

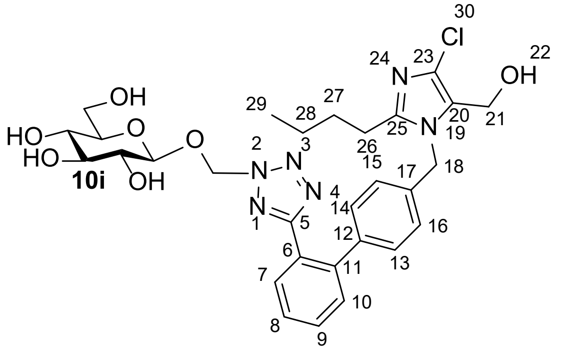

**10i**

**2*H*-(β-D-glucopyranosyloxymethyl)-Losartan (9i):** *Via* general synthetic procedure 2 starting from **9i** (35 mg, 0.034 mmol). Silicagel column chromatography (12% MeOH in DCM) of the residue afforded **10i** (17 mg, 81%) as a white amorphous solid. **TLC** (MeOH/DCM, 1/9, v/v) $R_f$ = 0.19; **HPLC**: Rt. 13.683 min; **$^1$H NMR** (400 MHz, MeOD) δ 7.82 (dd, *J* = 7.6, 1.4 Hz, 1H), 7.60 (td, *J* = 7.6, 1.5 Hz, 1H), 7.52 (td, *J* = 7.6, 1.4 Hz, 1H), 7.46 (dd, *J* = 7.6, 1.3 Hz, 1H), 7.23 – 7.12 (m, 2H), 7.10 – 7.01 (m, 2H), 6.19 (d, *J* = 11.3 Hz, 1H), 6.01 (d, *J* = 11.2 Hz, 1H), 5.32 (s, 2H), 4.50 (s, 2H), 4.43 (d, *J* = 7.7 Hz, 1H), 3.82 (dd, *J* = 12.0, 2.3 Hz, 1H), 3.65 (dd, *J* = 11.9, 5.6 Hz, 1H), 3.38 – 3.17 (m, 19H), 2.65 – 2.54 (m, 2H), 1.59 – 1.44 (m, 2H), 1.43 – 1.18 (m, 4H), 0.87 (t, *J* = 7.3 Hz, 4H); **$^{13}$C NMR** (101 MHz, MeOD) δ 166.96, 142.94, 136.87, 132.02, 131.74, 131.42, 130.88, 129.21, 128.72, 127.39, 127.14, 126.98, 125.75, 102.43 (C-1), 78.59, 78.38, 77.79, 74.50, 71.29, 52.44, 30.96, 28.95, 23.27, 14.05; **HRMS** (m/z): $[M+Na]^+$ calcd for $C_{31}H_{31}ClN_6O_7$, 637.2153; found, 637.2174.

## References


1. E. Krieger, K. Joo, J. Lee, J. Lee, S. Raman, J. Thompson, M. Tyka, D. Baker and K. Karplus, 2009, **77**, 114-122.
2. E. Krieger and G. Vriend, 2015, **36**, 996-1007.
3. U. Mückstein, I. L. Hofacker and P. F. J. B. Stadler, 2002, **18**, S153-S160.
4. J. Qiu and R. Elber, 2006, **62**, 881-891.
5. D. T. Jones, *Journal of Molecular Biology*, 1999, **292**, 195-202.
6. S. F. Altschul, T. L. Madden, A. A. Schäffer, J. Zhang, Z. Zhang, W. Miller and D. J. Lipman, *Nucleic Acids Research*, 1997, **25**, 3389-3402.
7. C. Wang, F. Ye, C. Chang, X. Liu, J. Wang, J. Wang, X.-F. Yan, Q. Fu, J. Zhou, S. Chen, Y.-G. Gao and L.-H. Zhang, *Proceedings of the National Academy of Sciences*, 2019, **116**, 22331.
8. Y. Hayashi, N. Okino, Y. Kakuta, T. Shikanai, M. Tani, H. Narimatsu and M. Ito, *Journal of Biological Chemistry*, 2007, **282**, 30889-30900.
9. Maxim V. Shapovalov and Roland L. Dunbrack, *Structure*, 2011, **19**, 844-858.

10. E. Krieger, R. L. Dunbrack, R. W. W. Hooft and B. Krieger, in *Computational Drug Discovery and Design*, ed. R. Baron, Springer New York, New York, NY, 2012, DOI: 10.1007/978-1-61779-465-0_25, pp. 405-421.
11. J. A. Maier, C. Martinez, K. Kasavajhala, L. Wickstrom, K. E. Hauser and C. Simmerling, *Journal of Chemical Theory and Computation*, 2015, **11**, 3696-3713.
12. J. Wang, R. M. Wolf, J. W. Caldwell, P. A. Kollman and D. A. Case, 2004, **25**, 1157-1174.
13. A. Jakalian, D. B. Jack and C. I. Bayly, *Journal of Computational Chemistry*, 2002, **23**, 1623-1641.
14. E. F. Pettersen, T. D. Goddard, C. C. Huang, G. S. Couch, D. M. Greenblatt, E. C. Meng and T. E. Ferrin, 2004, **25**, 1605-1612.
15. C. C. G. ULC, *Journal*, 2021.
16. D. Crich and F. Yang, *Angewandte Chemie International Edition*, 2009, **48**, 8896-8899.
17. L.-L. Gundersena and T. Benneche, *Acta Chemica Scandinavica*, 1991, **45**, 975-977.
18. S. M. Andersen, M. Heuckendorff and H. H. Jensen, *Organic Letters*, 2015, **17**, 944-947.
19. Z. Song, L. Meng, Y. Xiao, X. Zhao, J. Fang, J. Zeng and Q. Wan, *Green Chemistry*, 2019, **21**, 1122-1127.
20. D. Semeria, M. Philippe, J.-M. Delaumeny, A.-M. Sepulchre and S. D. Gero, *Synthesis*, 1983, **1983**, 710-713.
21. L. J. van den Bos, T. J. Boltje, T. Provoost, J. Mazurek, H. S. Overkleeft and G. A. van der Marel, *Tetrahedron Letters*, 2007, **48**, 2697-2700.
22. T. Ueda, M. Hayashi, Y. Ikeuchi, T. Nakajima, E. Numagami and S. Kobayashi, *Organic Process Research & Development*, 2014, **18**, 1728-1739.
23. C.-W. T. Chang, Y. Hui, B. Elchert, J. Wang, J. Li and R. Rai, *Organic Letters*, 2002, **4**, 4603-4606.
24. M. Mori, Y. Ito and T. Ogawa, *Carbohydrate Research*, 1990, **195**, 199-224.
25. H. Yu and X. Chen, *Organic Letters*, 2006, **8**, 2393-2396.
26. S.-F. Lu, Q. O'Yang, Z.-W. Guo, B. Yu and Y.-Z. Hui, *The Journal of Organic Chemistry*, 1997, **62**, 8400-8405.
27. Q.-L. He, I. Minn, Q. Wang, P. Xu, S. A. Head, E. Datan, B. Yu, M. G. Pomper and J. O. Liu, 2016, **55**, 12035-12039.

## $^{1}H$- and $^{13}C$-NMR spectra

500 MHz, $CDCl_3$

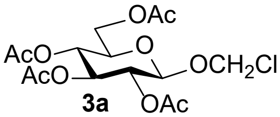


**3a**

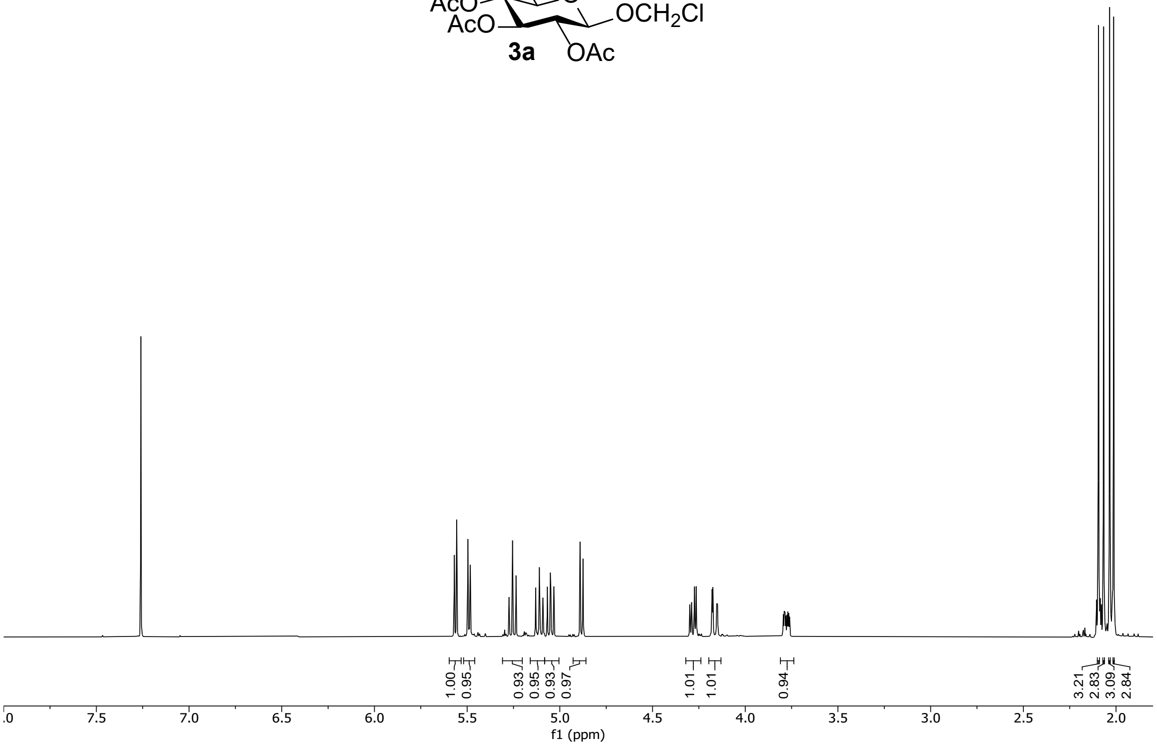


125 MHz, $CDCl_3$

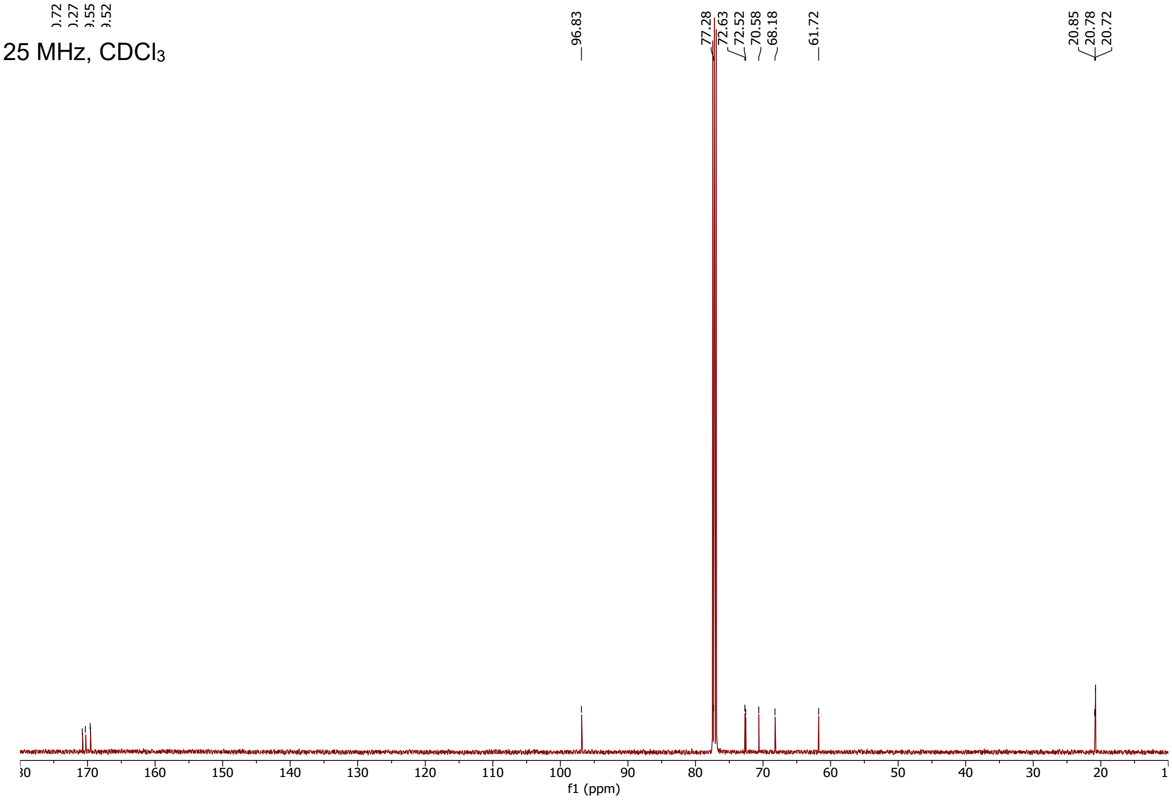

400 MHz, MeOD

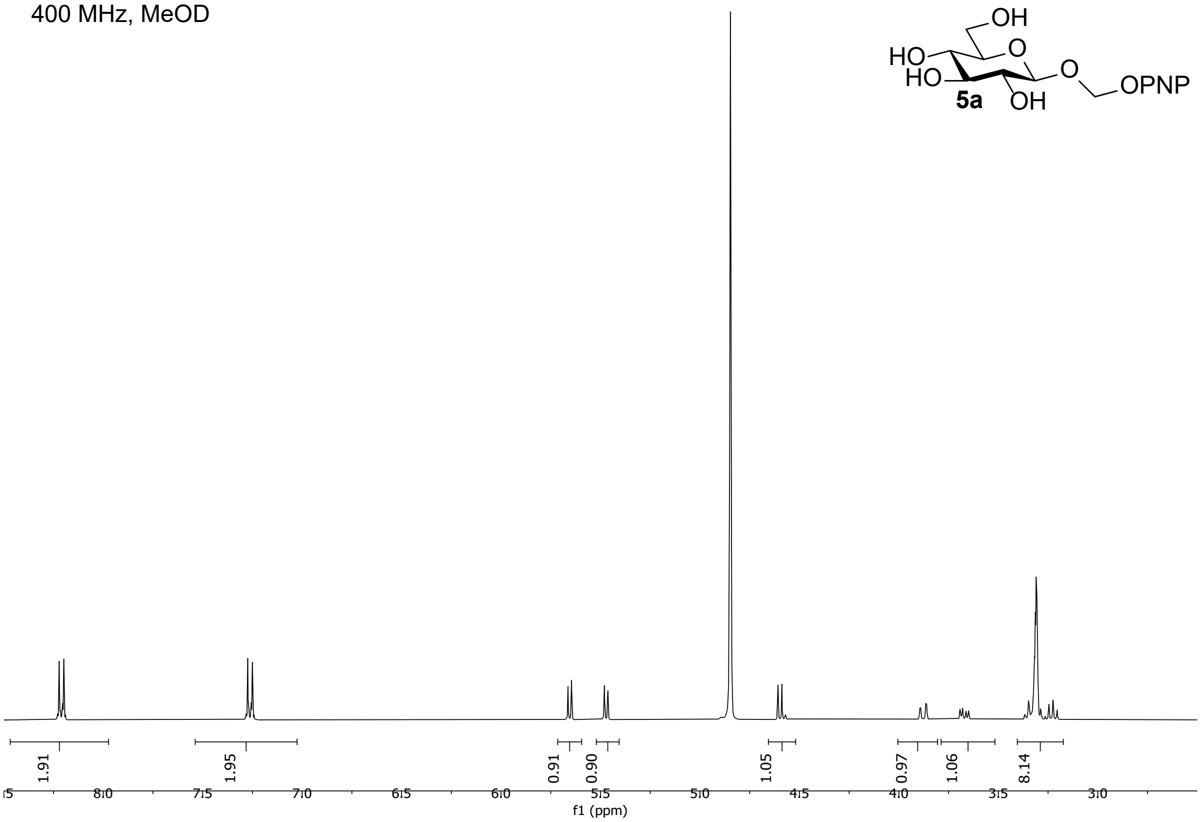


5a

101 MHz, MeOD

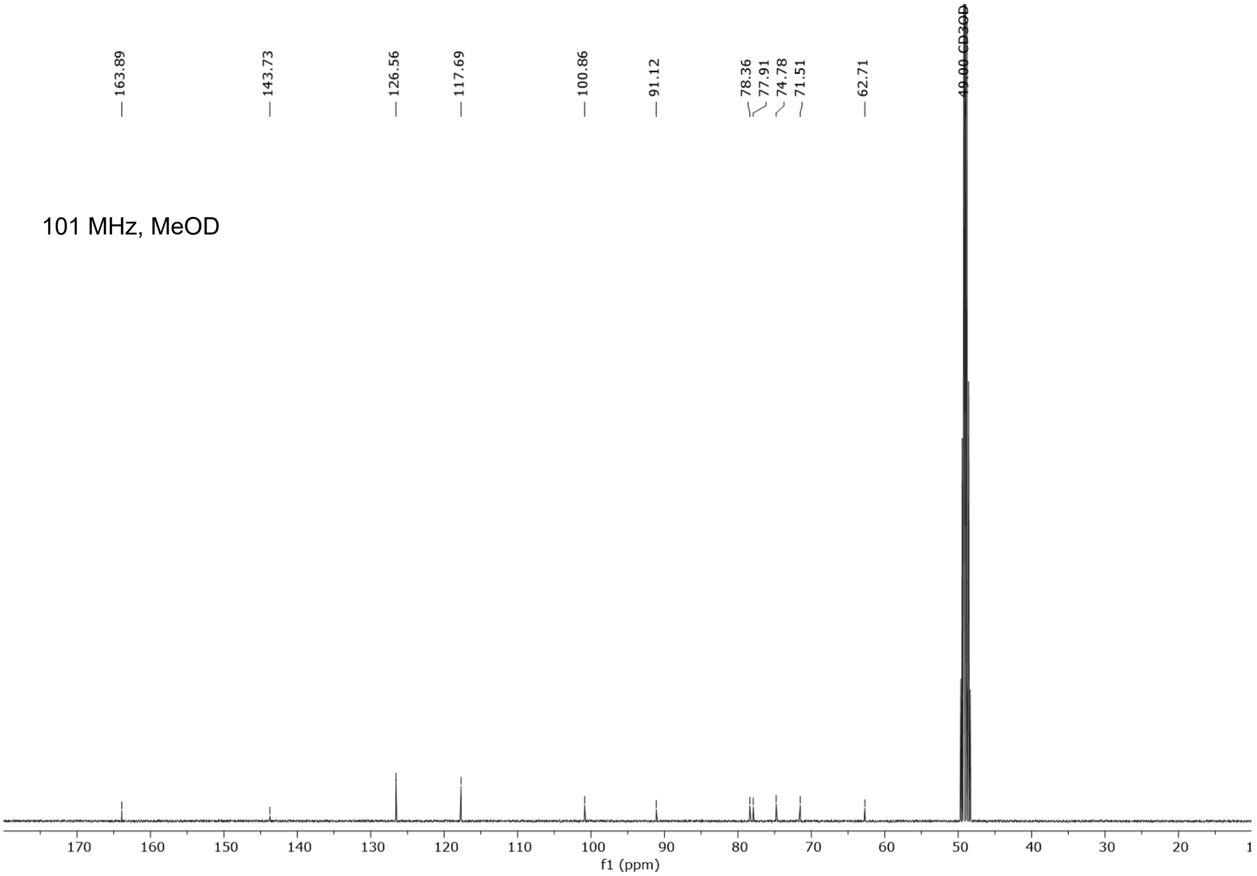

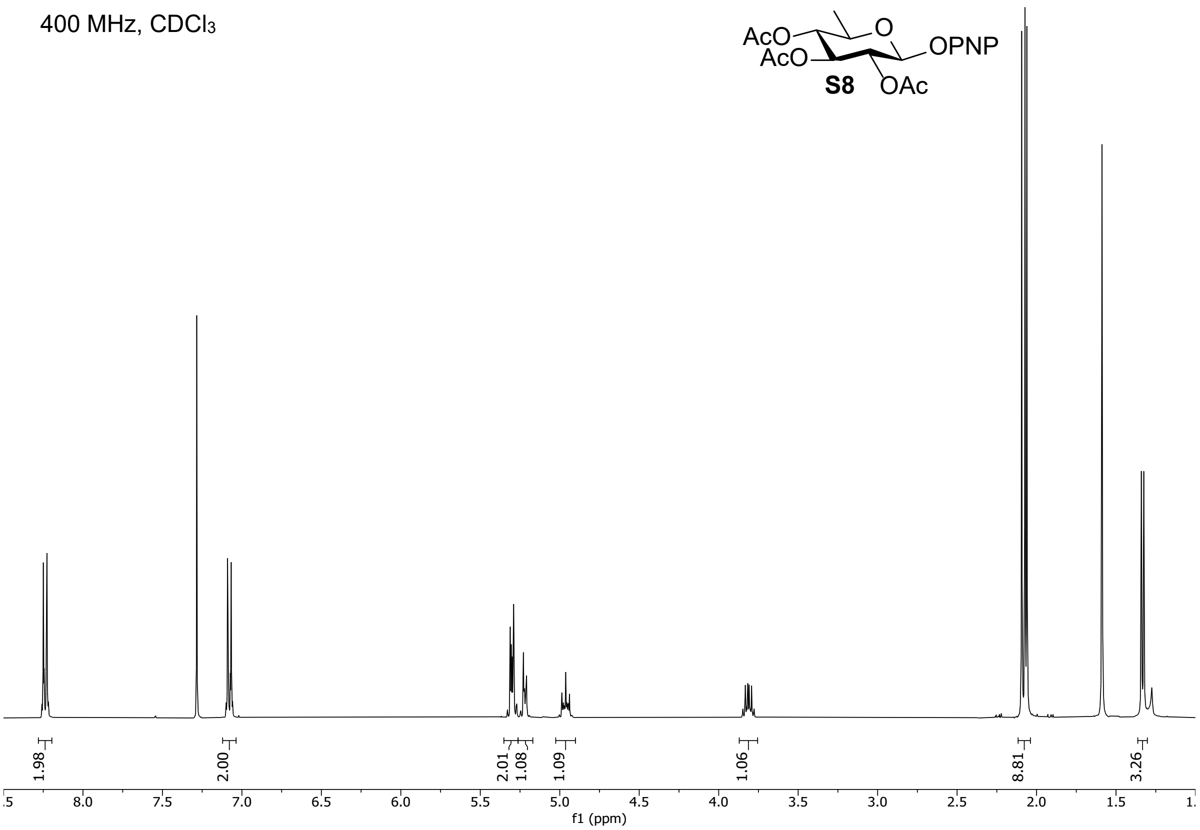
400 MHz, CDCl3
AcO
AcO
O
OPNP
S8
OAc
1.98
2.00
2.01
1.08
1.09
1.06
8.81
3.26
f1 (ppm)

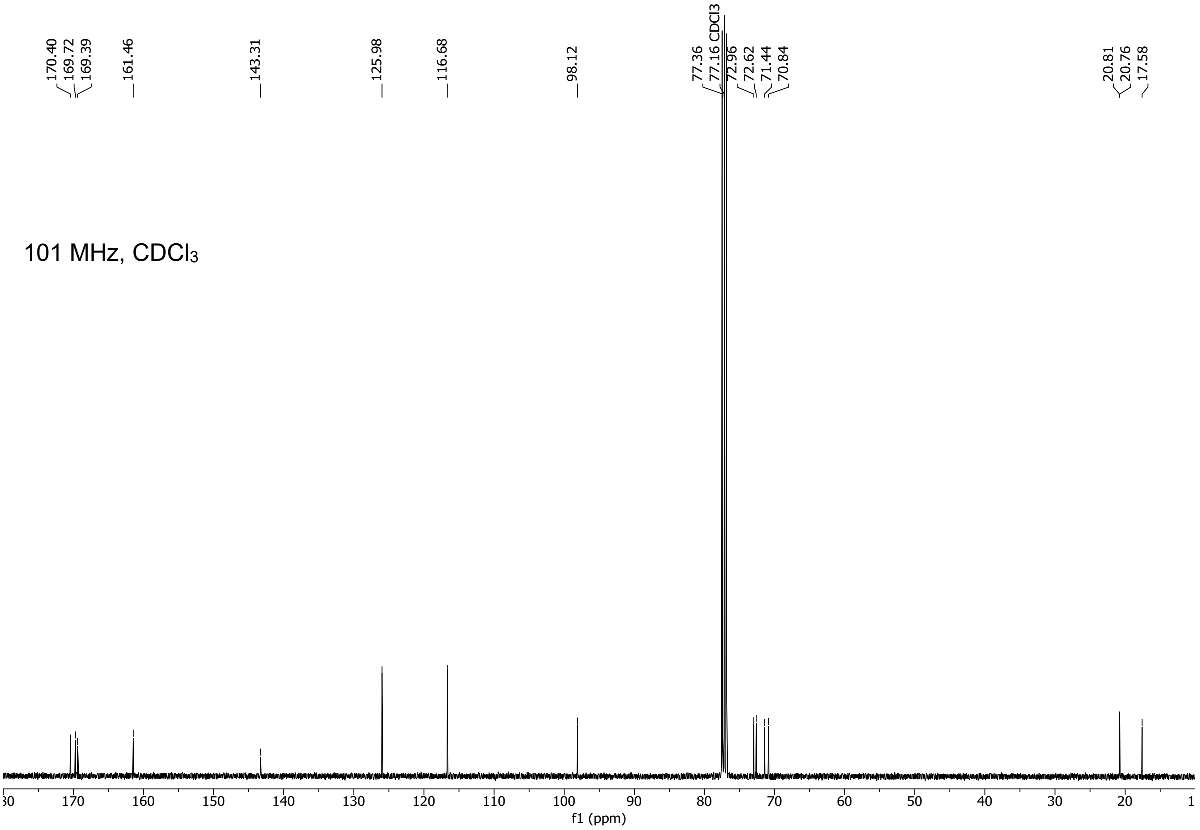
101 MHz, CDCl3
170.40
169.72
169.39
161.46
143.31
125.98
116.68
98.12
77.36
77.16 CDCl3
72.96
72.62
71.44
70.84
20.81
20.76
17.58
f1 (ppm)

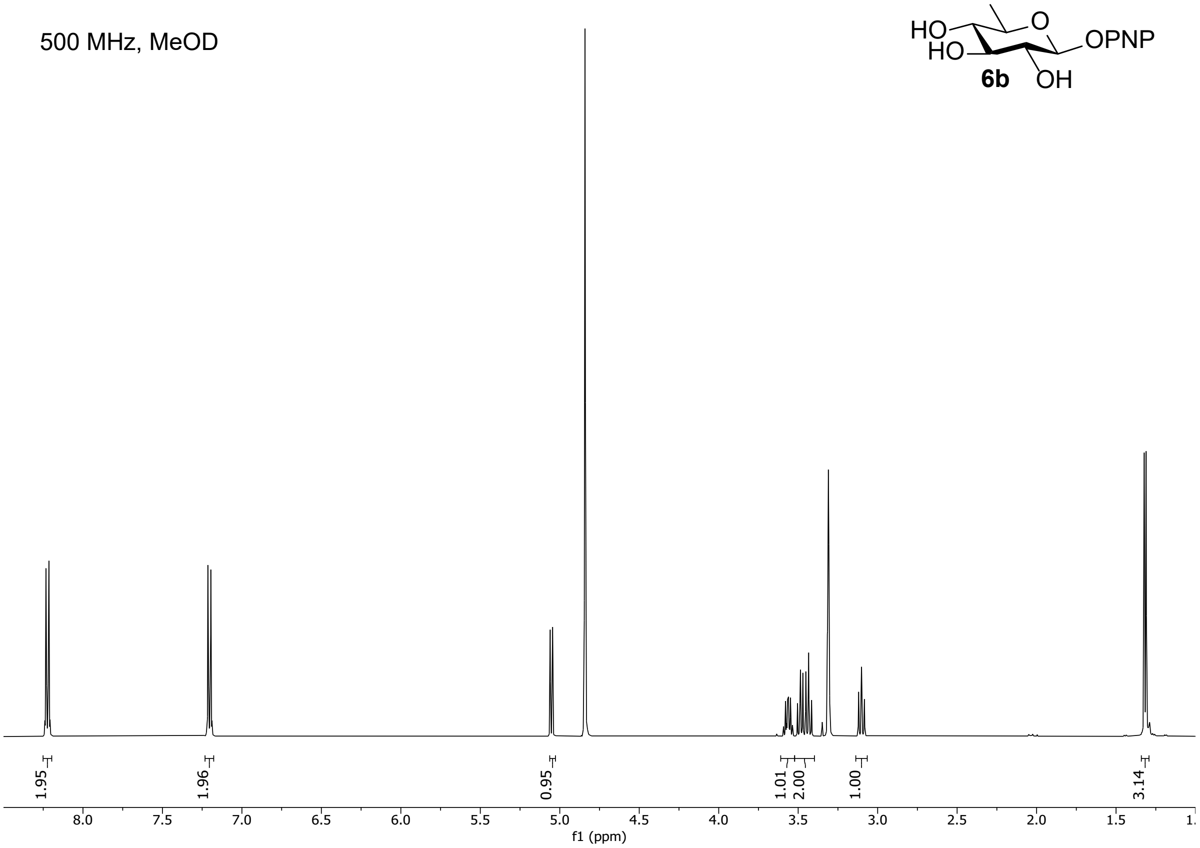
HO
HO
O
OPNP
6b
OH
500 MHz, MeOD
1.95
1.96
0.95
1.01
2.00
1.00
3.14
8.0 7.5 7.0 6.5 6.0 5.5 5.0 4.5 4.0 3.5 3.0 2.5 2.0 1.5
f1 (ppm)

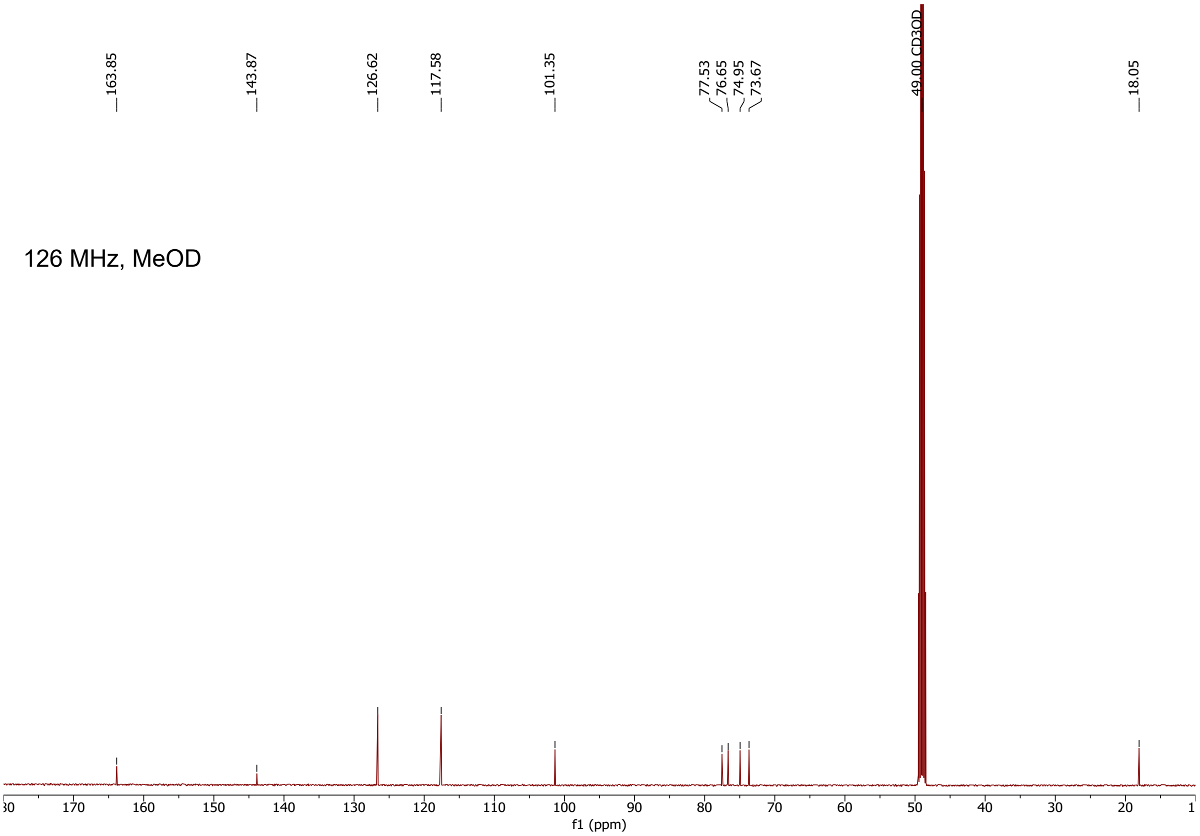
163.85
143.87
126.62
117.58
101.35
77.53
76.65
74.95
73.67
49.00 CD3OD
18.05
126 MHz, MeOD
170 160 150 140 130 120 110 100 90 80 70 60 50 40 30 20
f1 (ppm)

400 MHz, CDCl$_3$

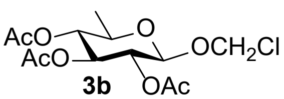


**3b**

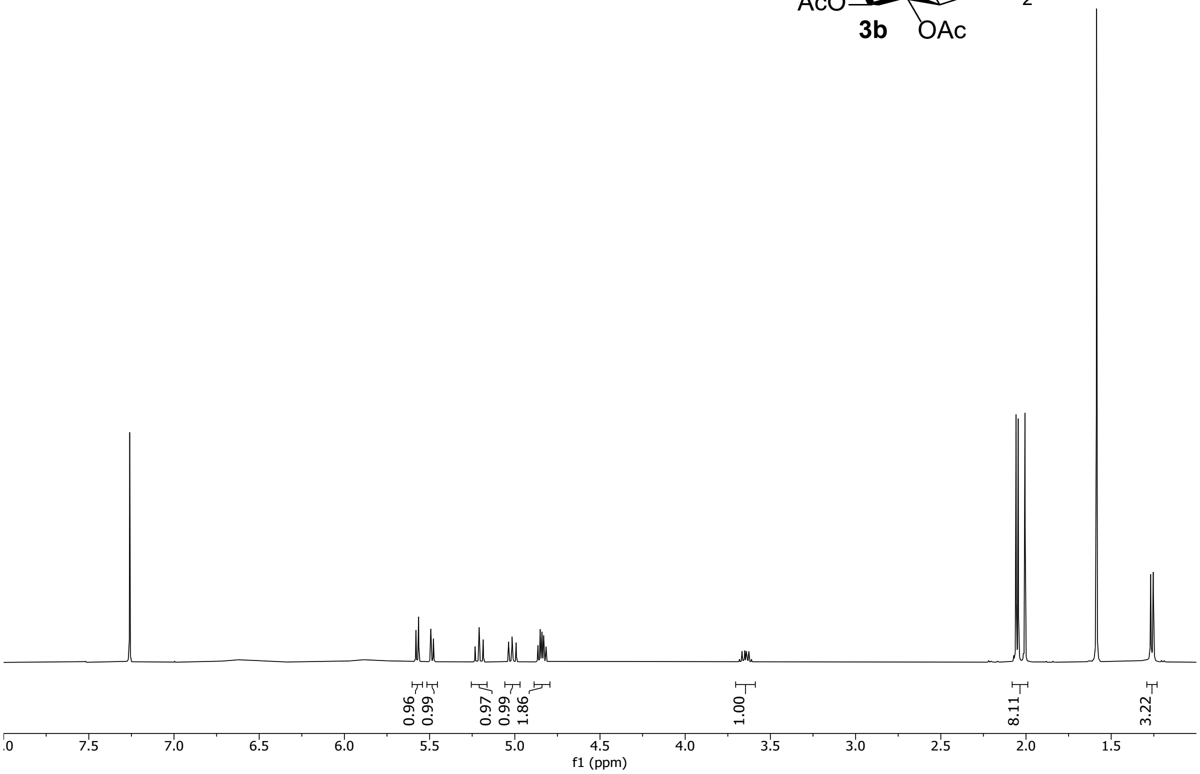


101 MHz, CDCl$_3$

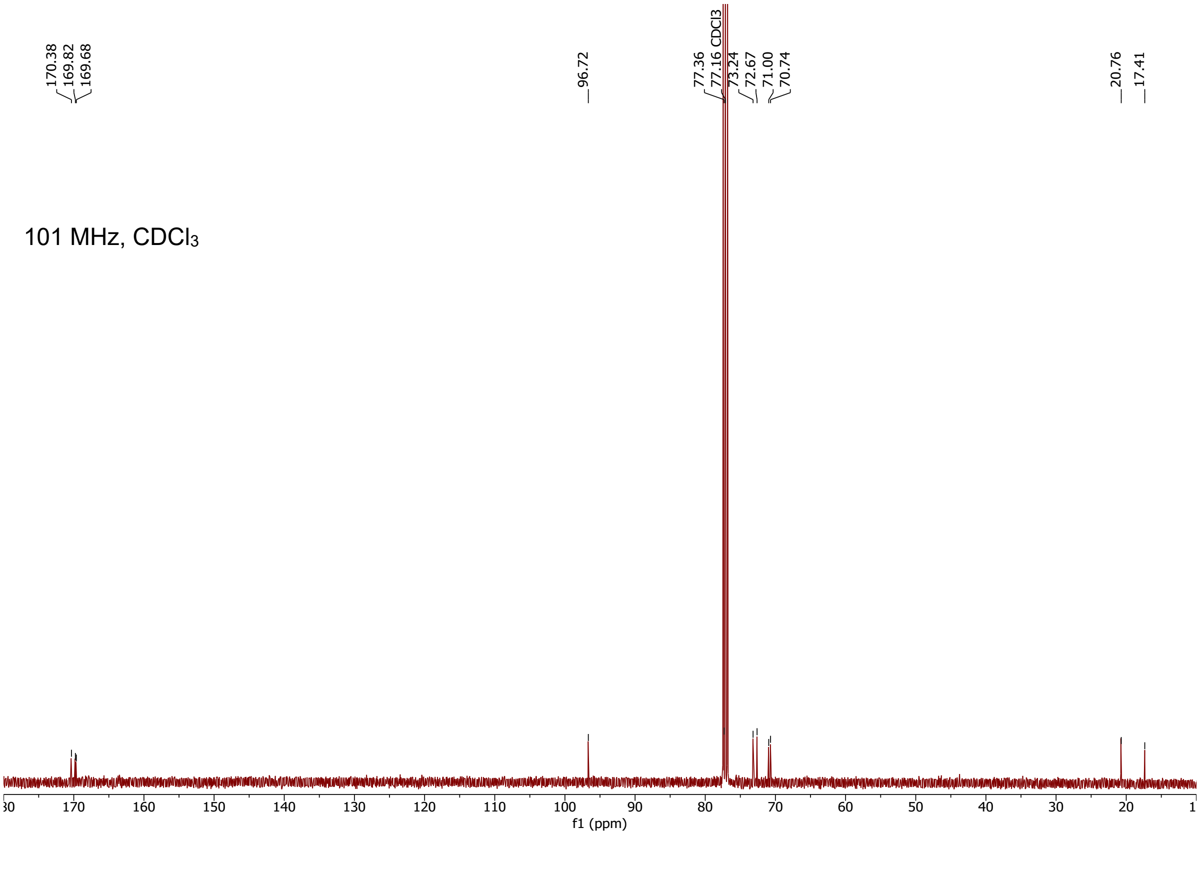

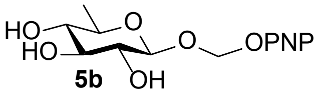
HO
HO
O
O
OPNP
5b
OH

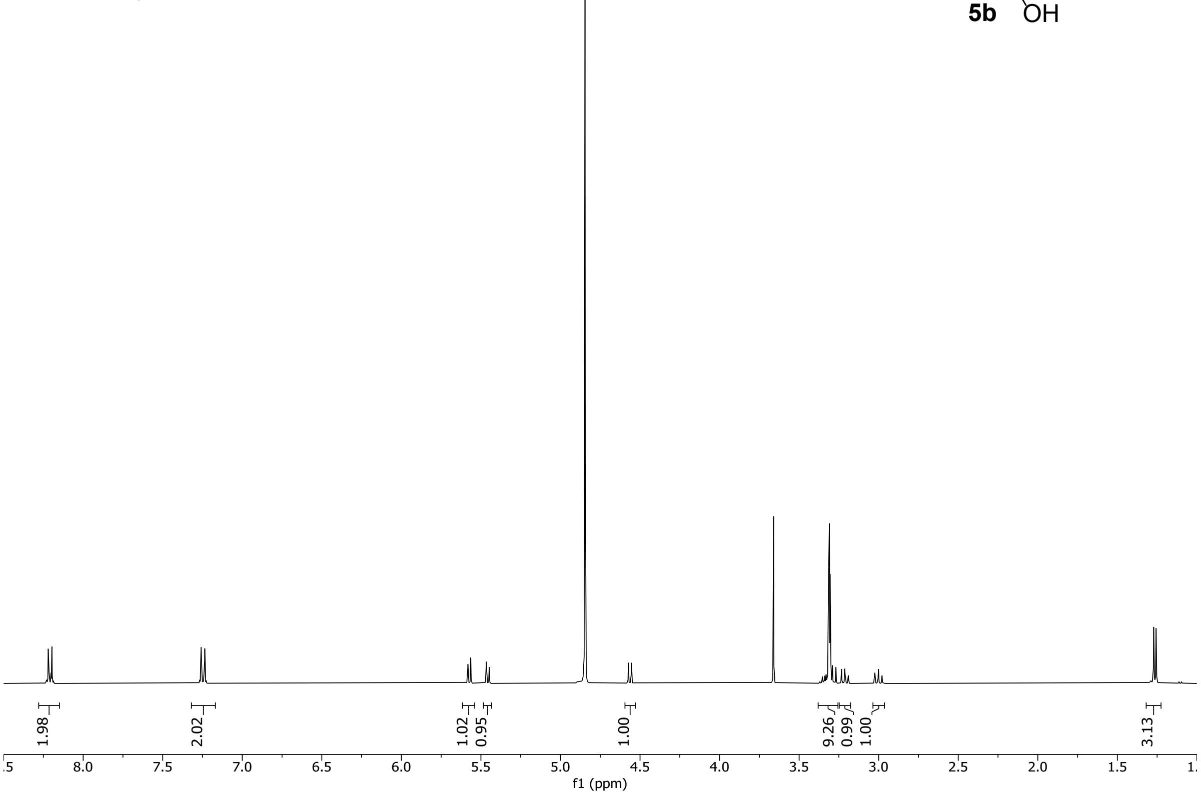
400 MHz, MeOD
1.98
2.02
1.02
0.95
1.00
9.26
0.99
1.00
3.13
8.0
7.5
7.0
6.5
6.0
5.5
5.0
4.5
4.0
3.5
3.0
2.5
2.0
1.5
f1 (ppm)

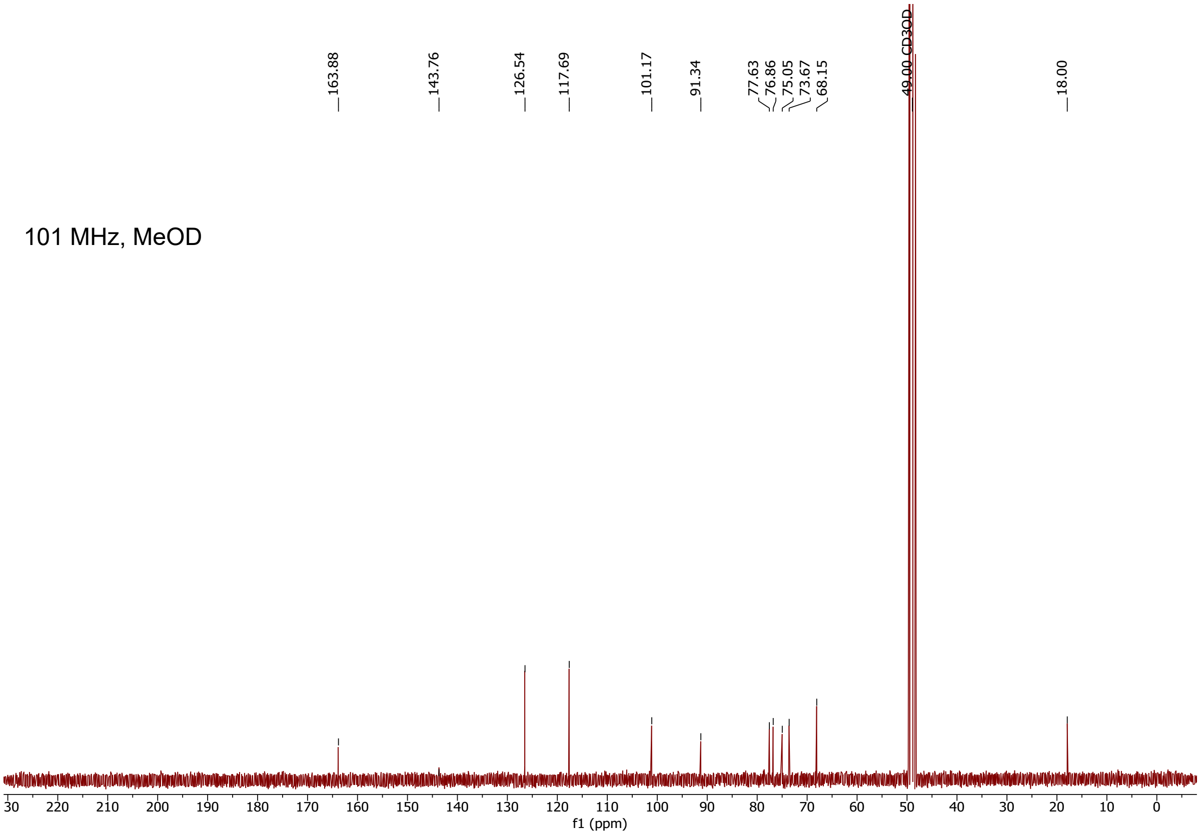
163.88
143.76
126.54
117.69
101.17
91.34
77.63
76.86
75.05
73.67
68.15
49.00 CD3OD
18.00
101 MHz, MeOD
220
210
200
190
180
170
160
150
140
130
120
110
100
90
80
70
60
50
40
30
20
10
0
f1 (ppm)

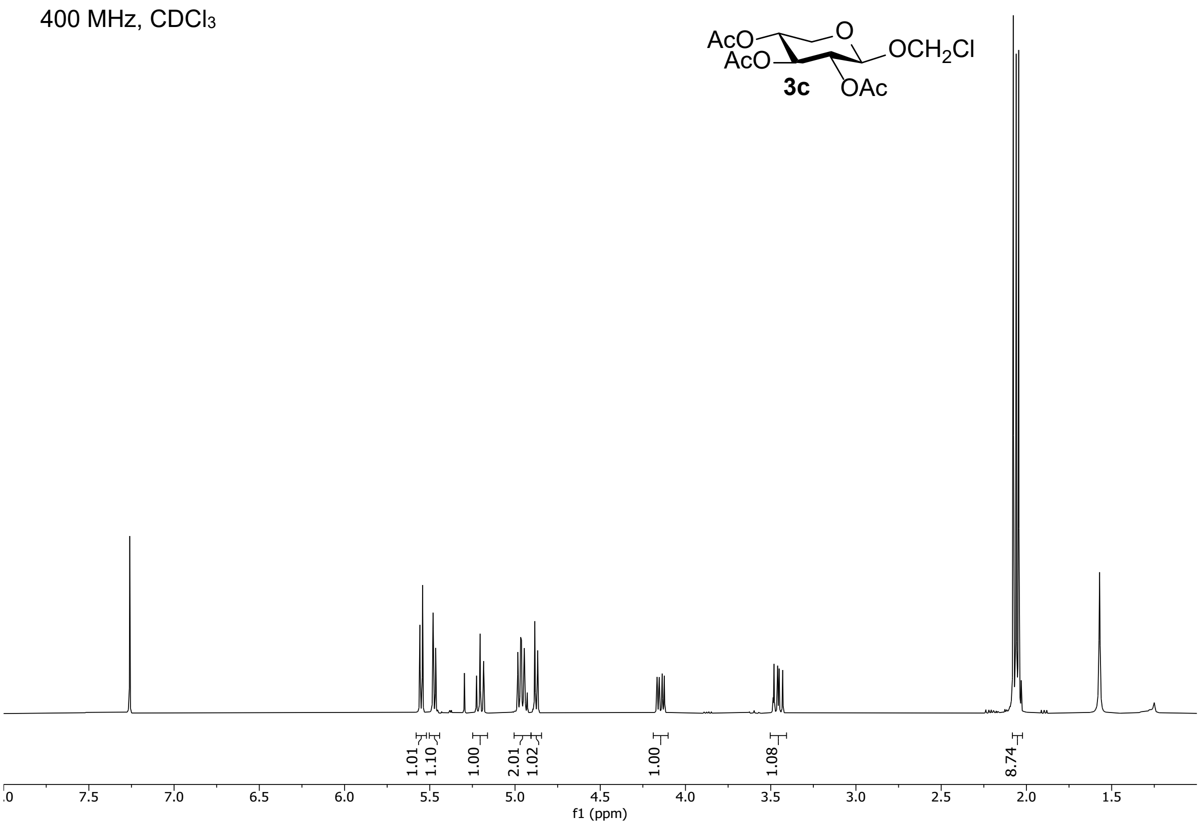
400 MHz, CDCl3
AcO
AcO
O
OCH2Cl
OAc
3c
1.01
1.10
1.00
2.01
1.02
1.00
1.08
8.74
7.5
7.0
6.5
6.0
5.5
5.0
4.5
4.0
3.5
3.0
2.5
2.0
1.5
f1 (ppm)

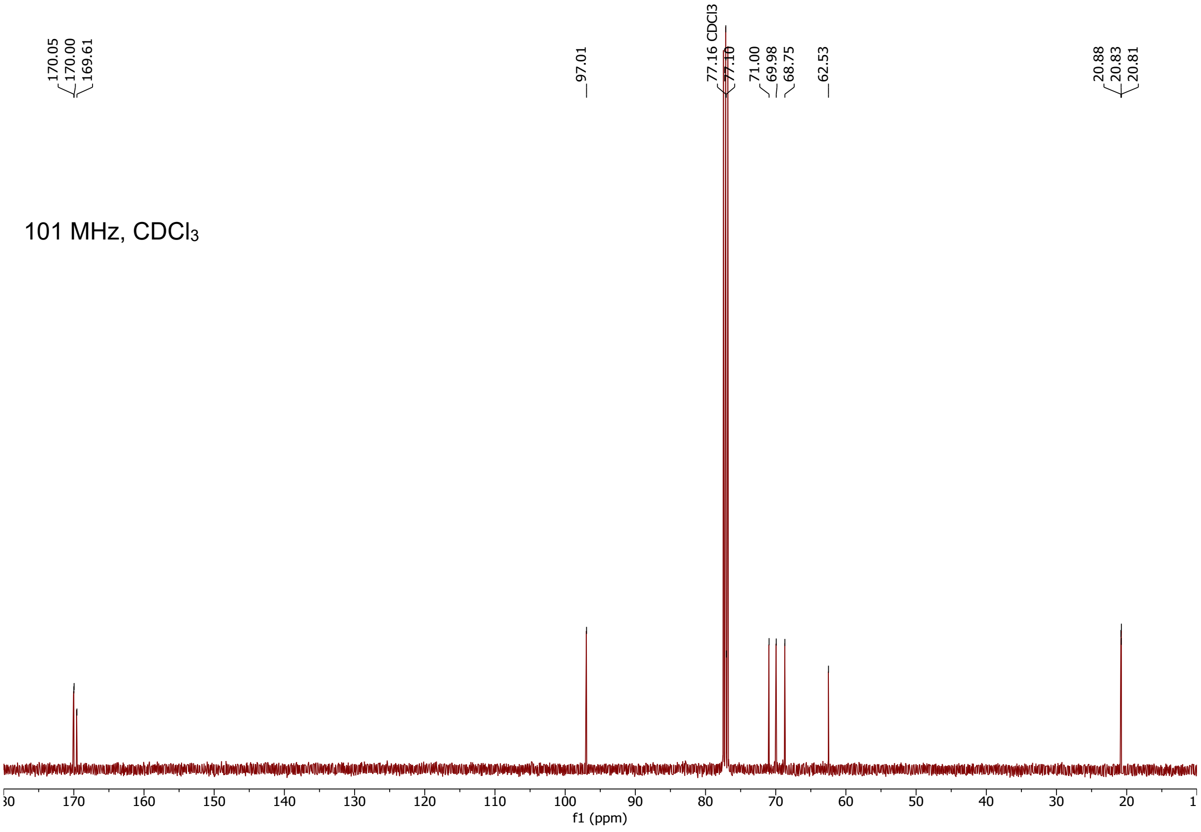
170.05
170.00
169.61
97.01
77.16 CDCl3
77.10
71.00
69.98
68.75
62.53
20.88
20.83
20.81
101 MHz, CDCl3
170
160
150
140
130
120
110
100
90
80
70
60
50
40
30
20
f1 (ppm)

500 MHz, MeOD

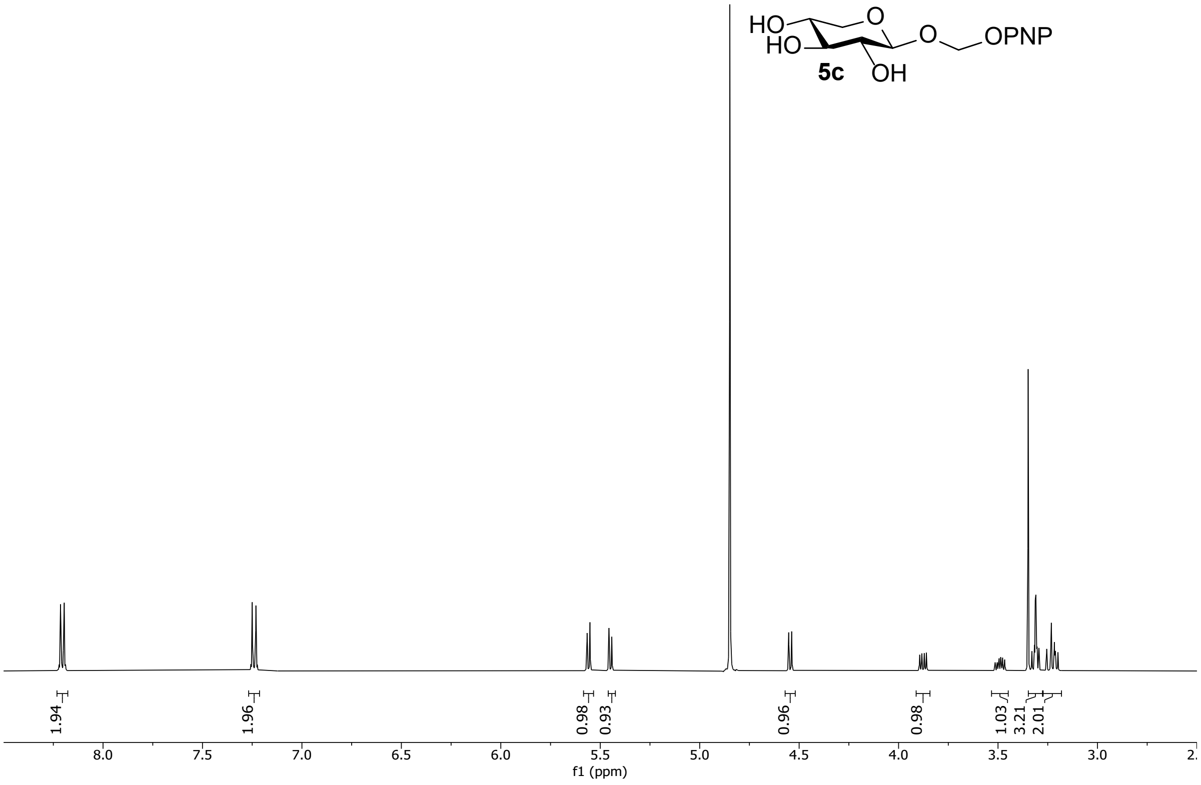


126 MHz, MeOD

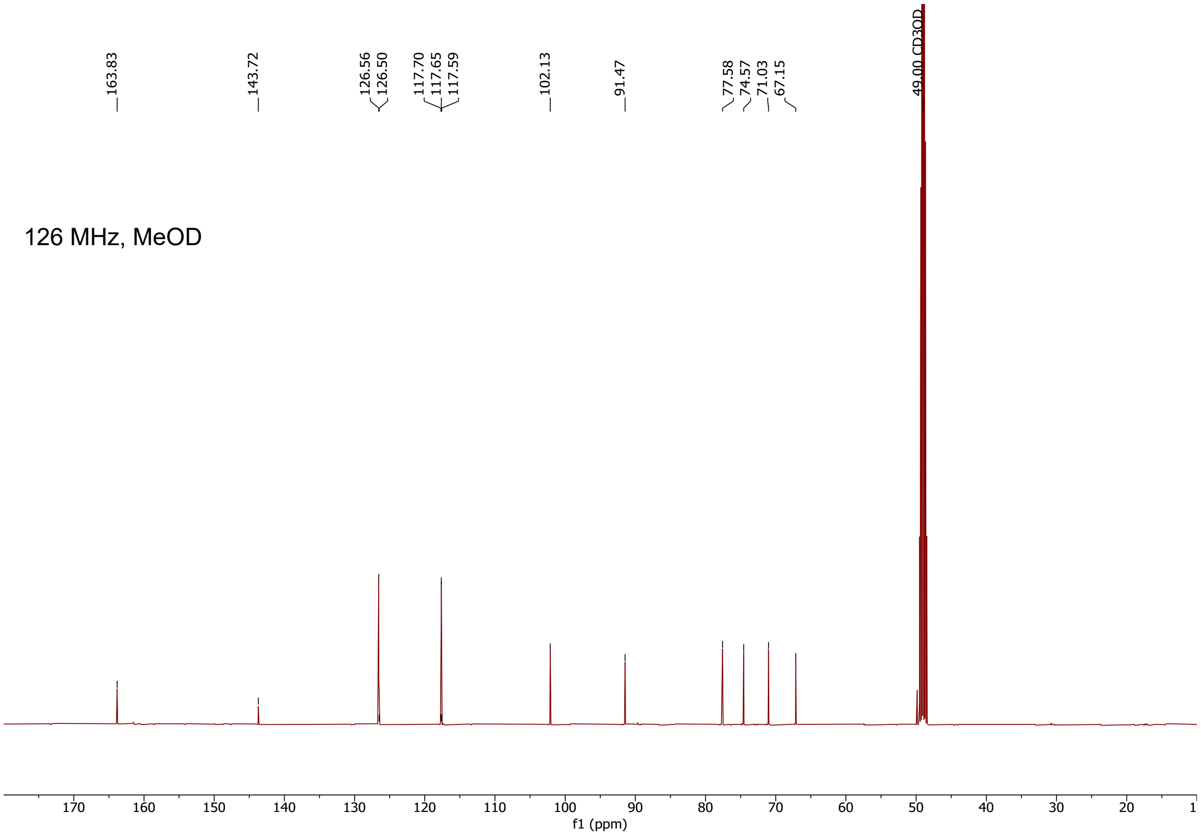

400 MHz, $CDCl_3$

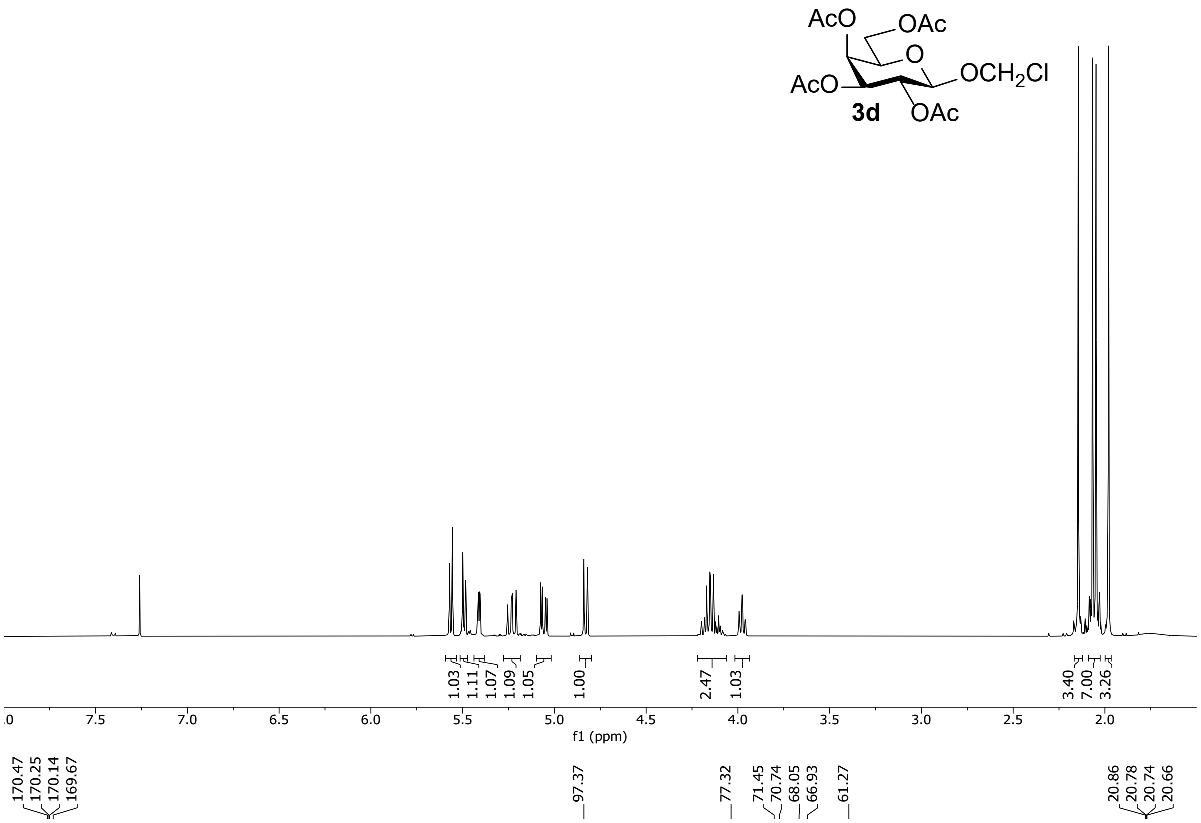


101 MHz, $CDCl_3$

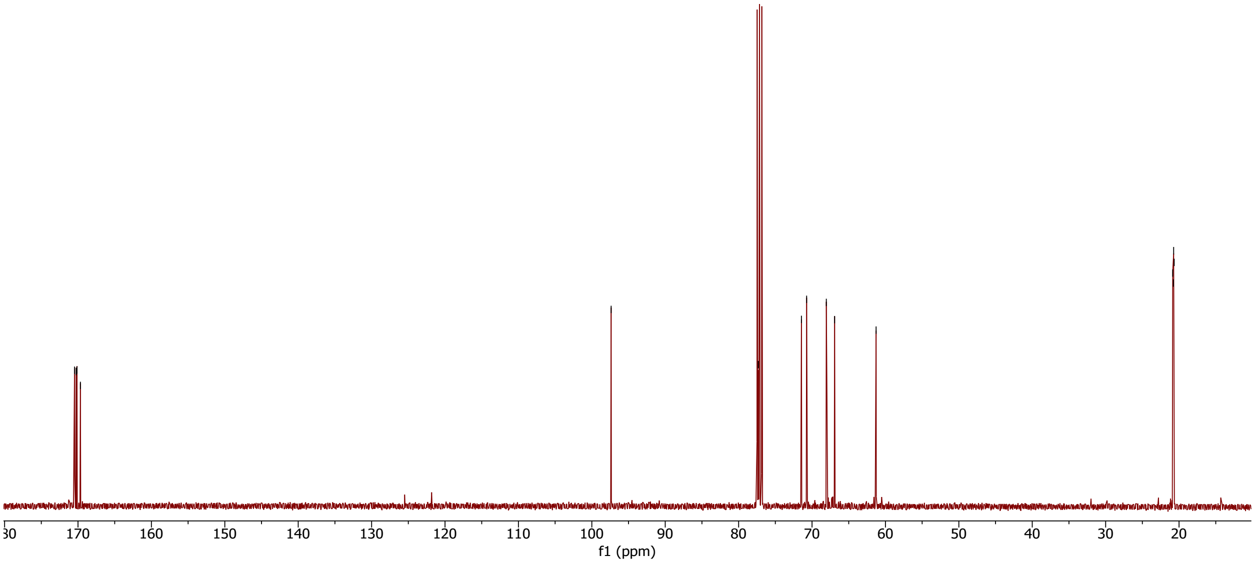

500 MHz, MeOD

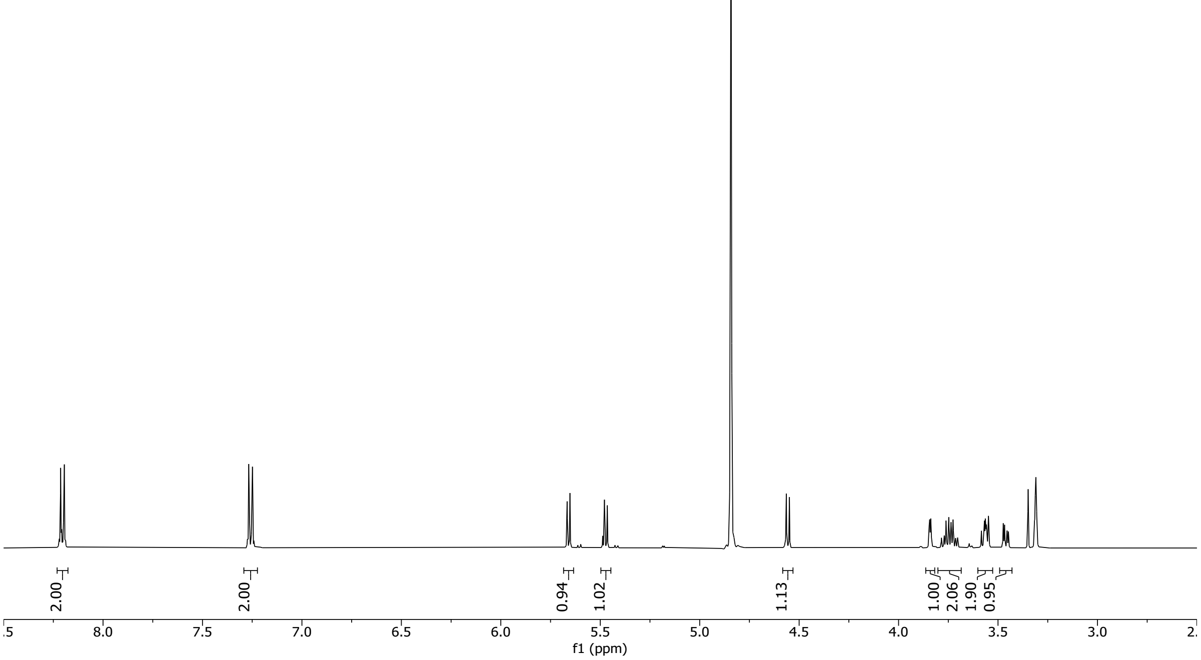


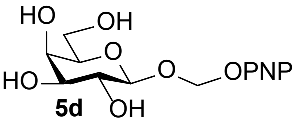


**5d**

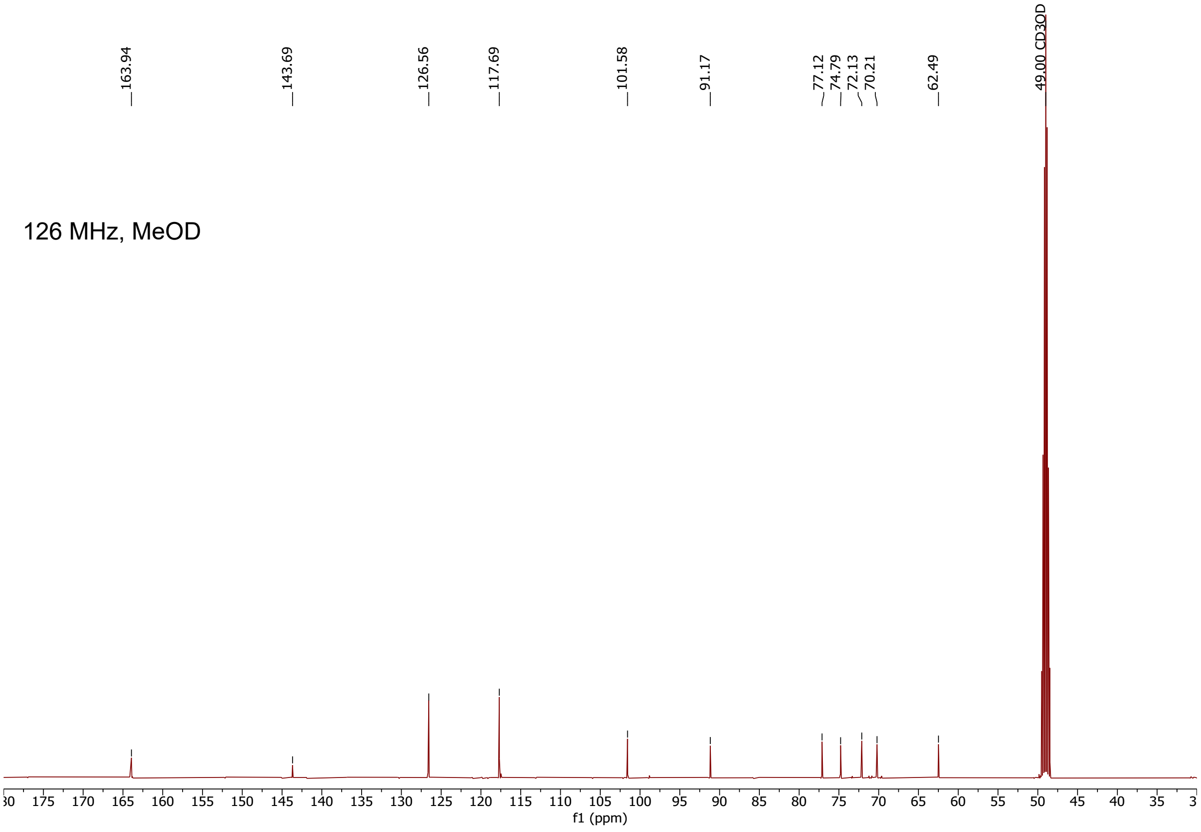

400 MHz, $CDCl_3$

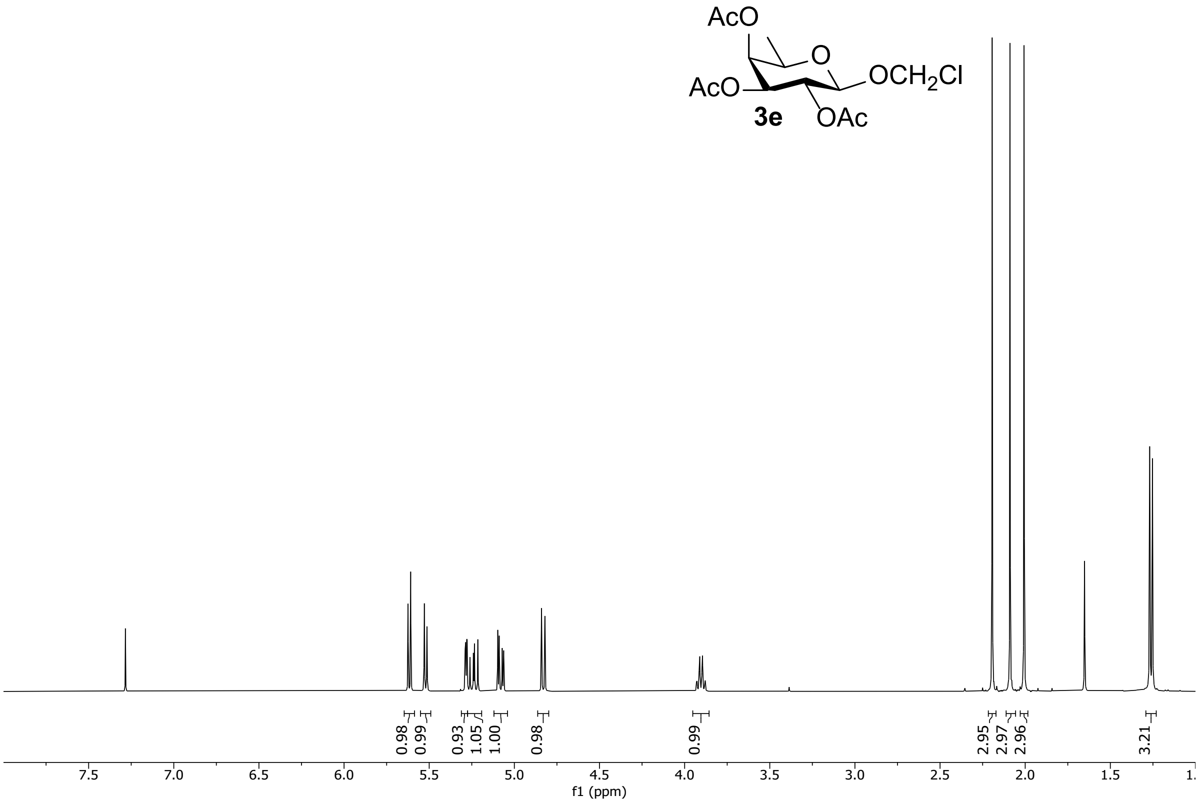


101 MHz, $CDCl_3$

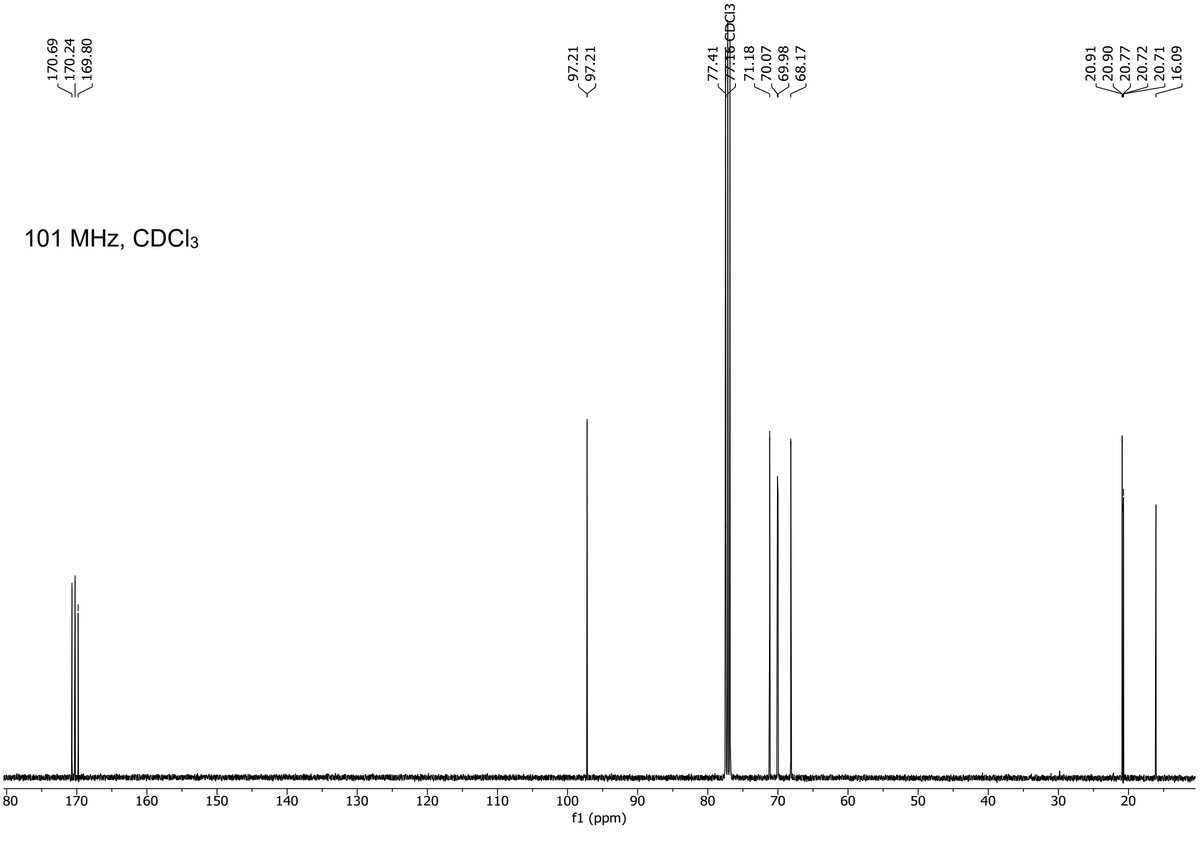

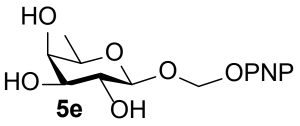
HO
O
O
OPNP
HO
5e
OH

400 MHz, MeOD

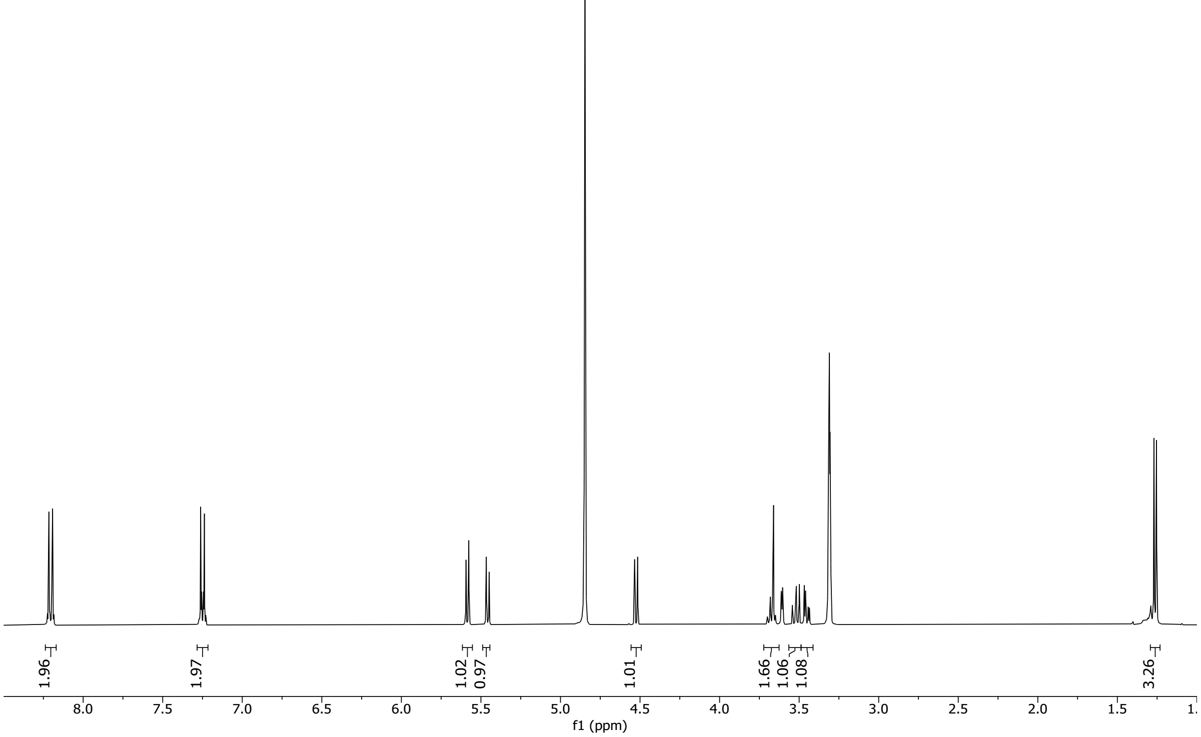
1.96
1.97
1.02
0.97
1.01
1.66
1.06
1.08
3.26
f1 (ppm)

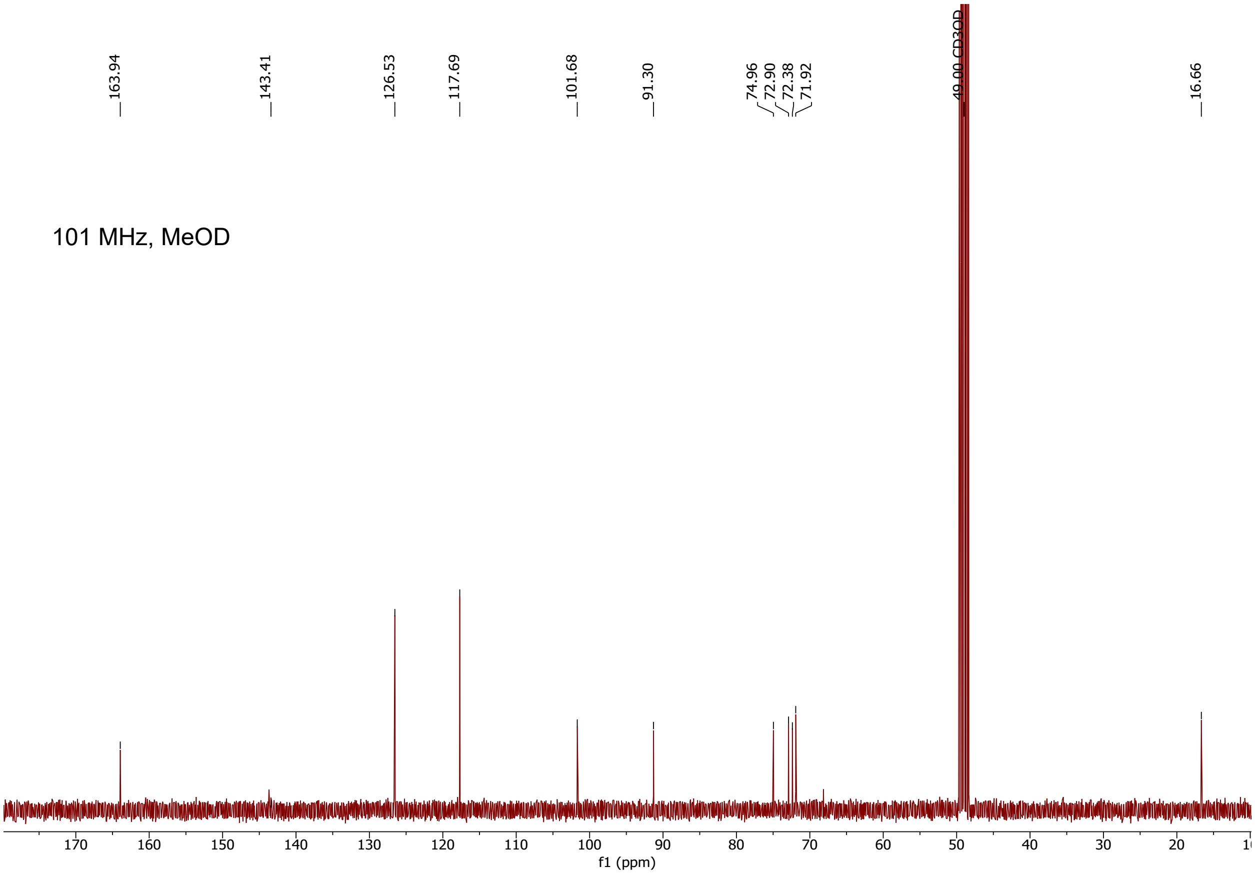
163.94
143.41
126.53
117.69
101.68
91.30
74.96
72.90
72.38
71.92
49.00 CD3OD
16.66
101 MHz, MeOD
f1 (ppm)

500 MHz, $CDCl_3$

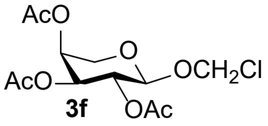


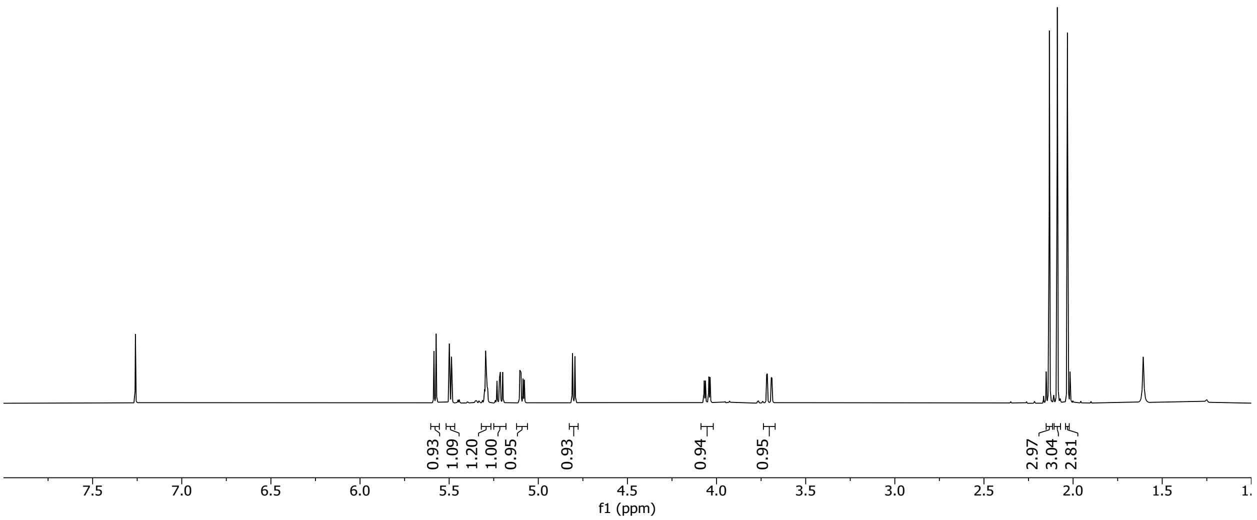


126 MHz, $CDCl_3$

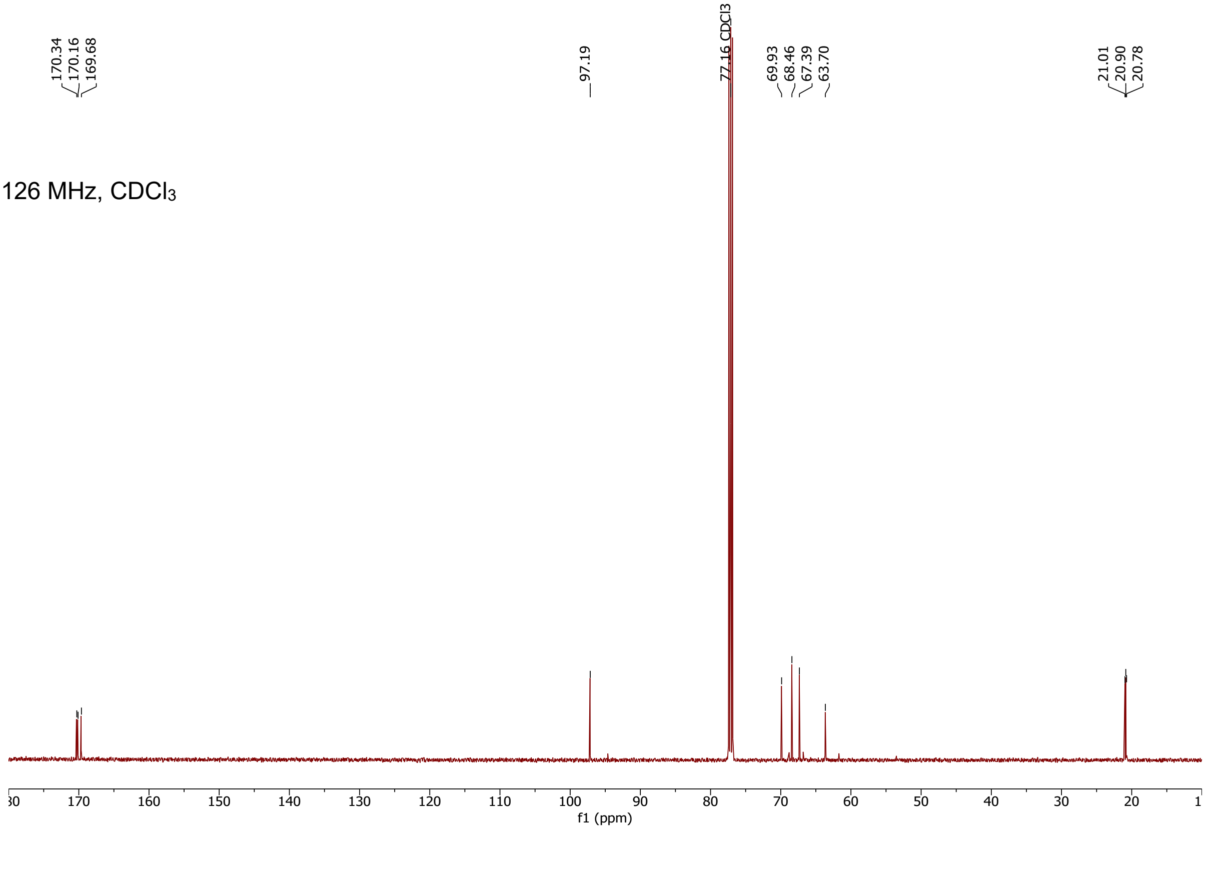

500 MHz, MeOD

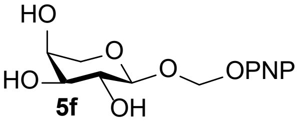


**5f**

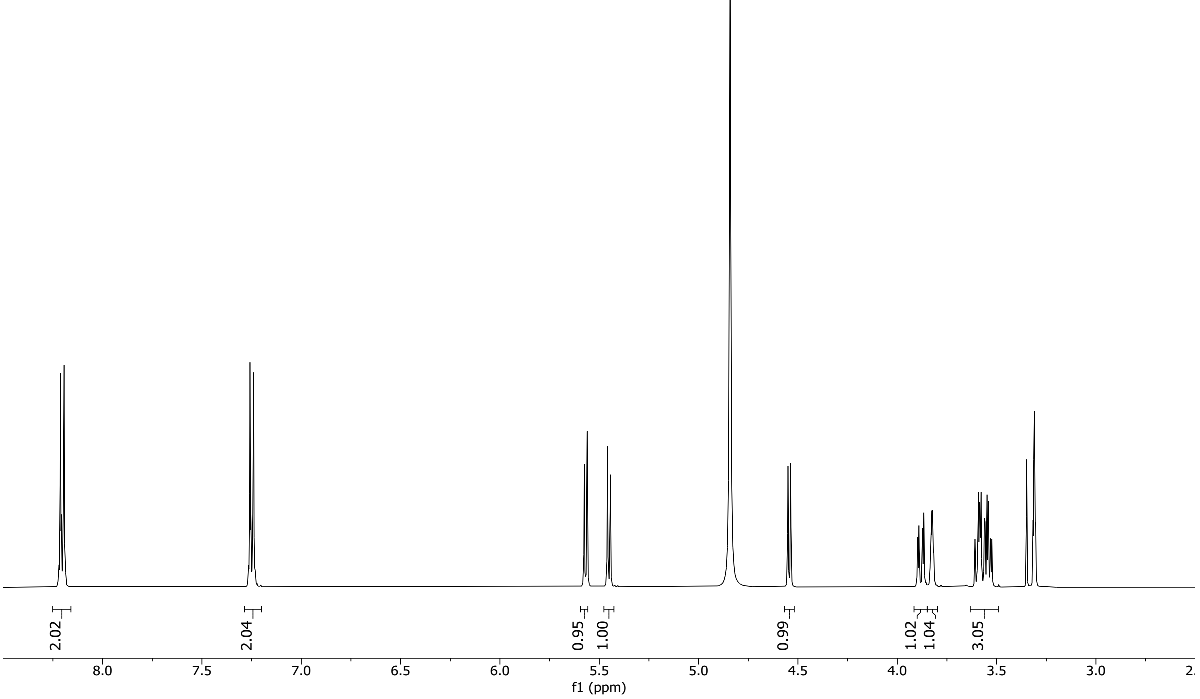


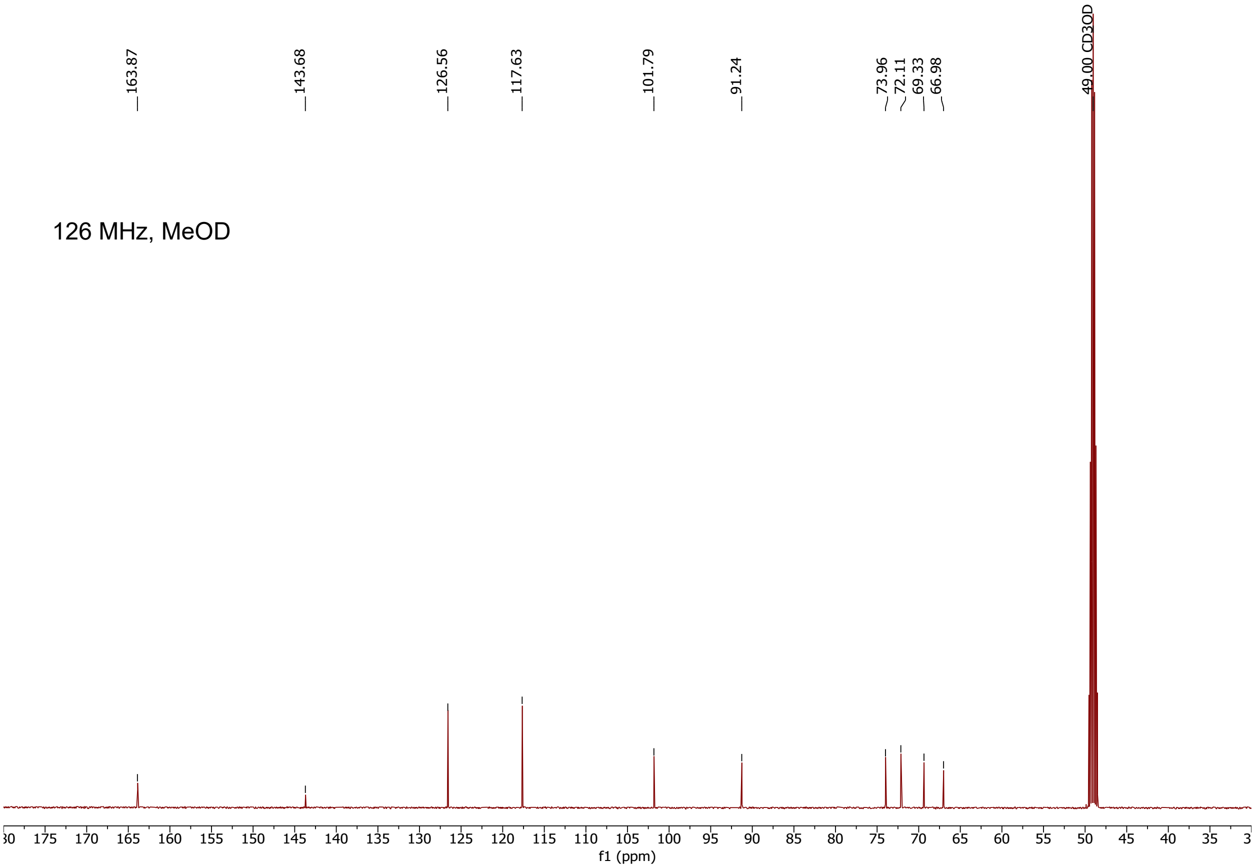

400 MHz, $CDCl_3$

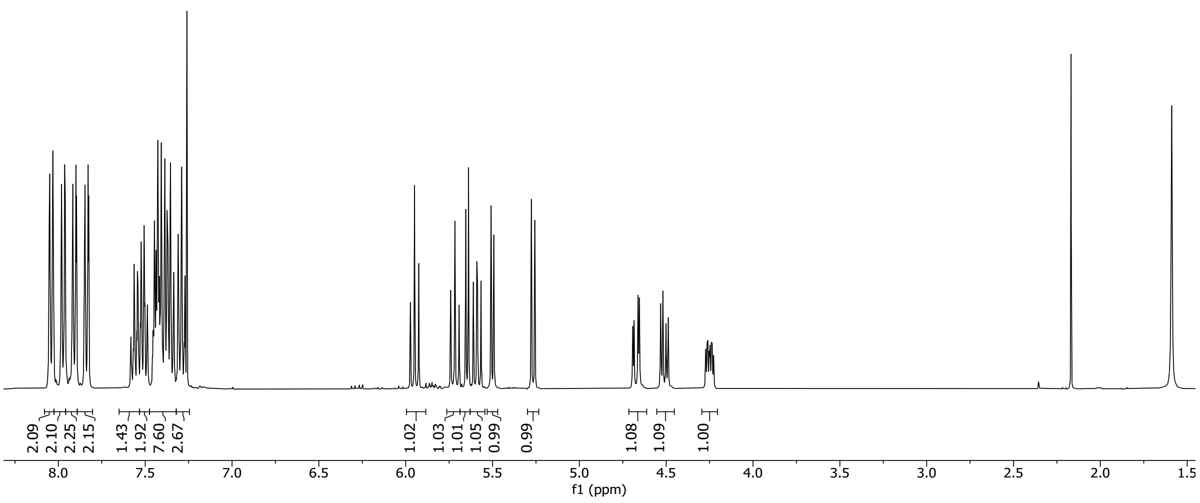

101 MHz, $CDCl_3$

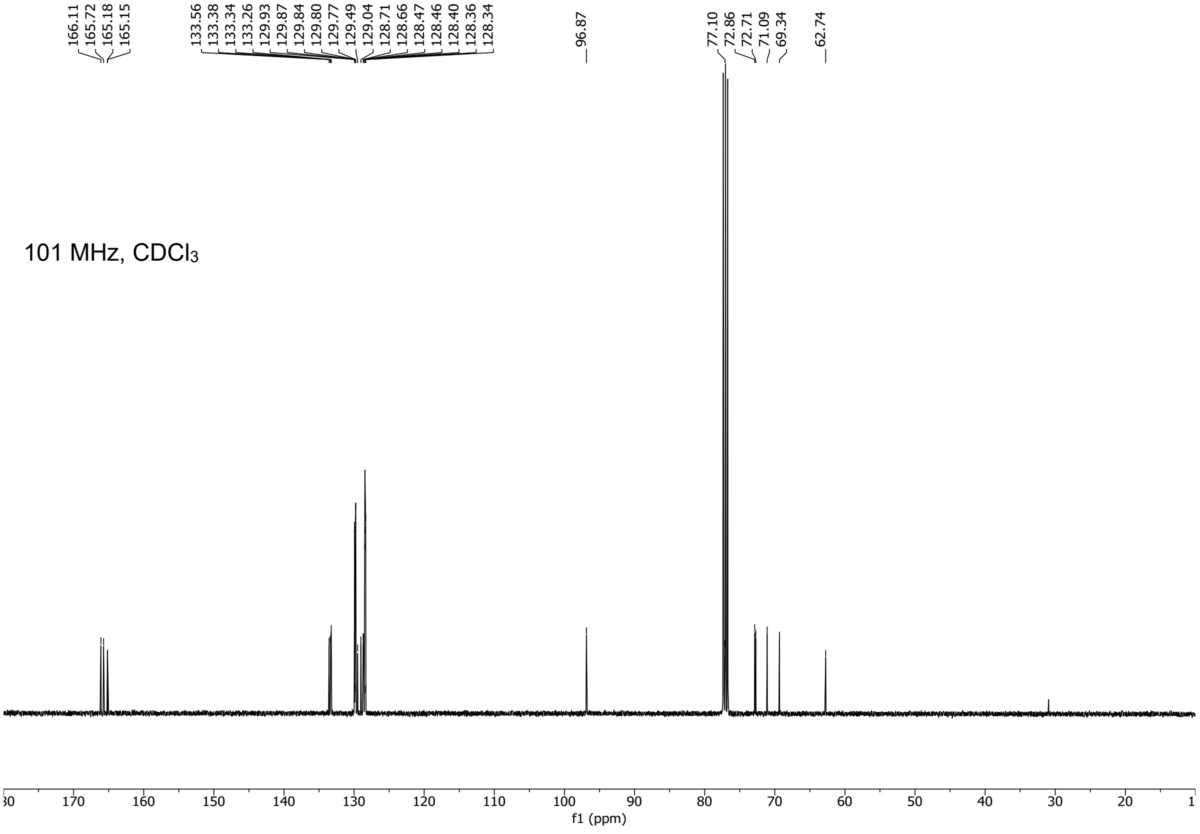

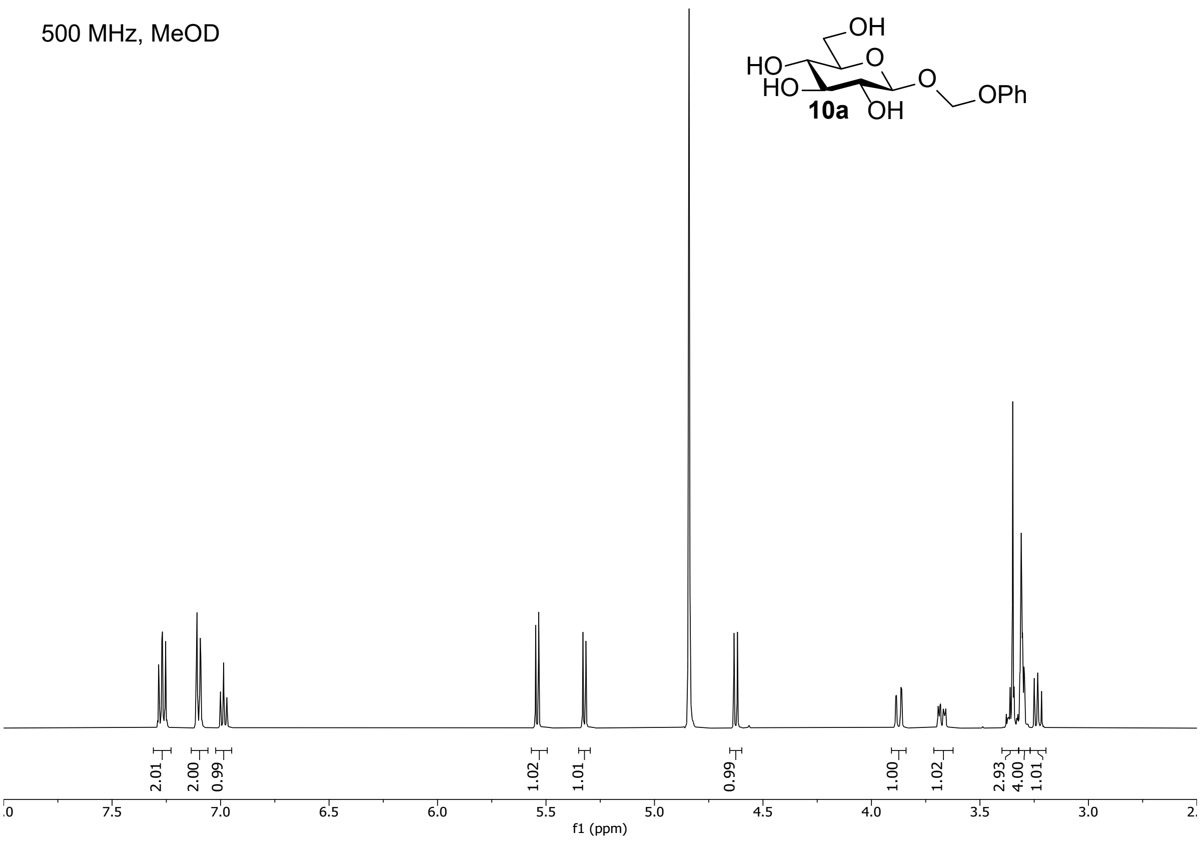
500 MHz, MeOD
HO
HO
OH
O
O
OPh
10a
OH
2.01
2.00
0.99
1.02
1.01
0.99
1.00
1.02
2.93
4.00
1.01
f1 (ppm)

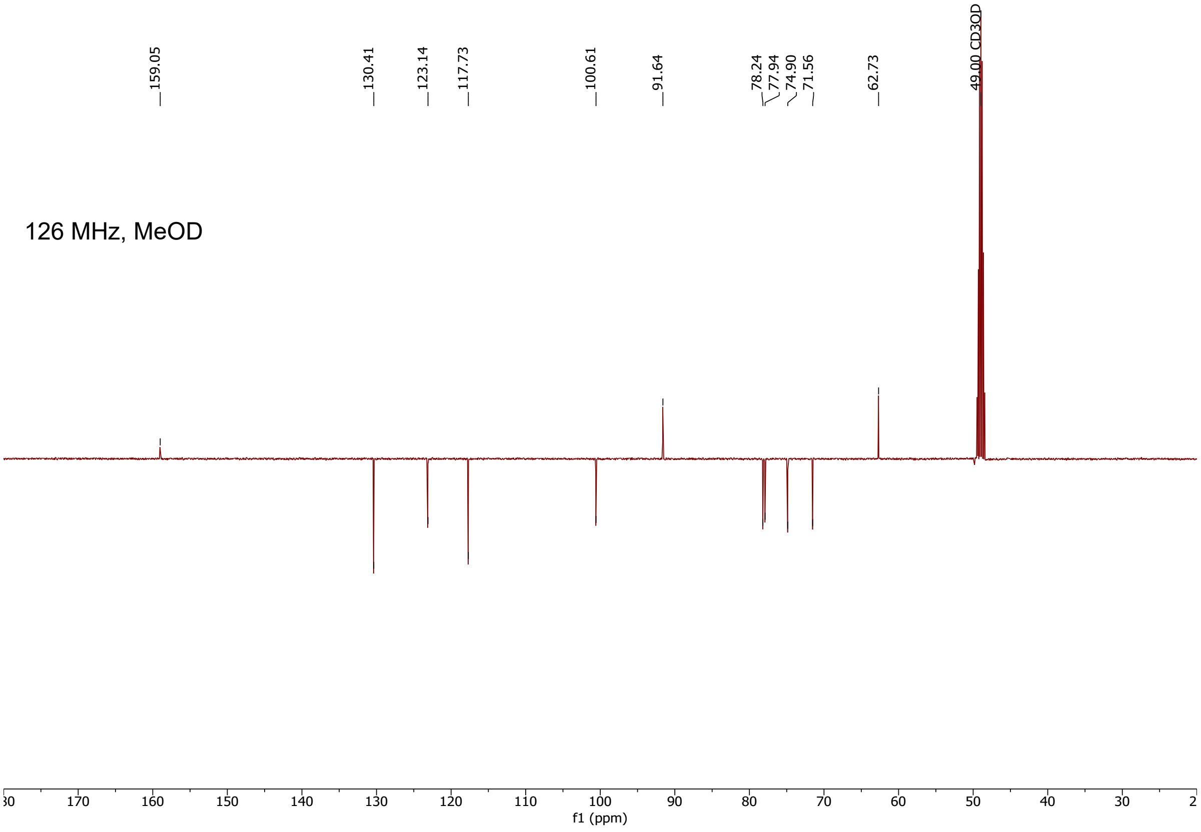
159.05
130.41
123.14
117.73
100.61
91.64
78.24
77.94
74.90
71.56
62.73
49.00 CD3OD
126 MHz, MeOD
f1 (ppm)

500 MHz, MeOD

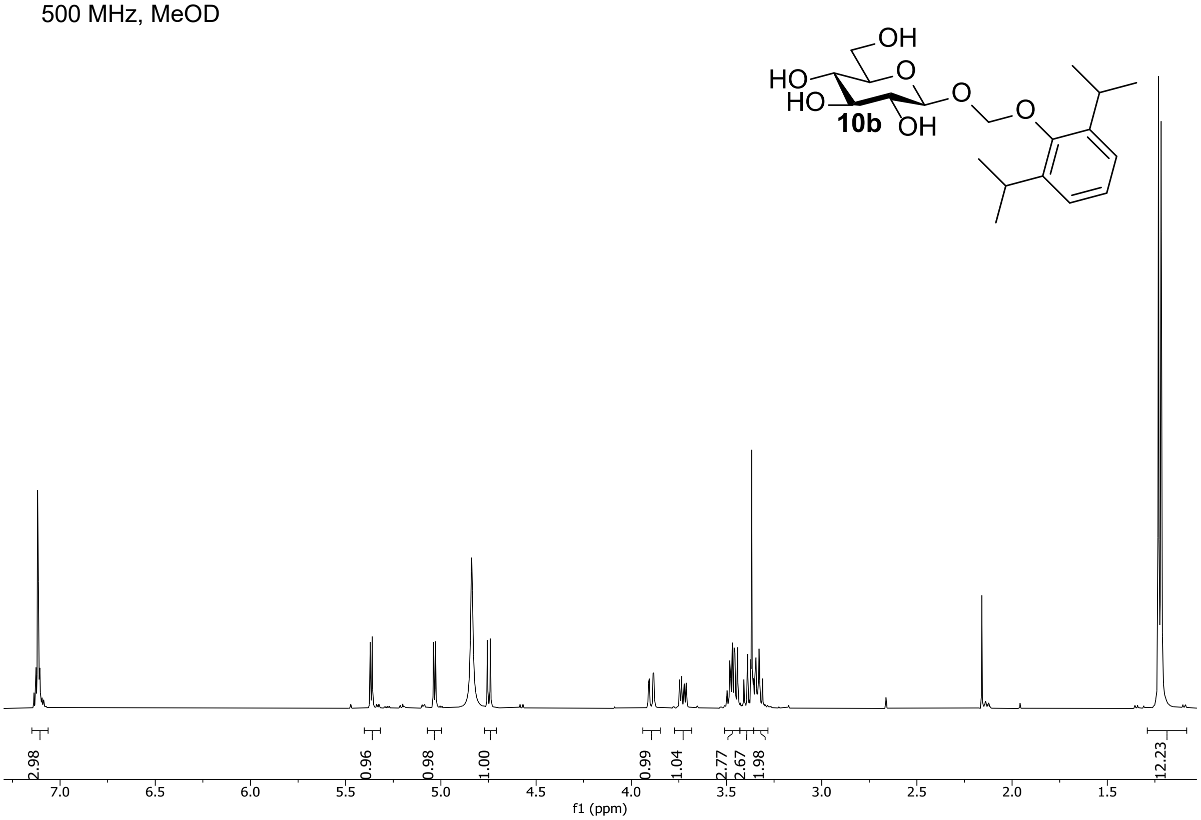


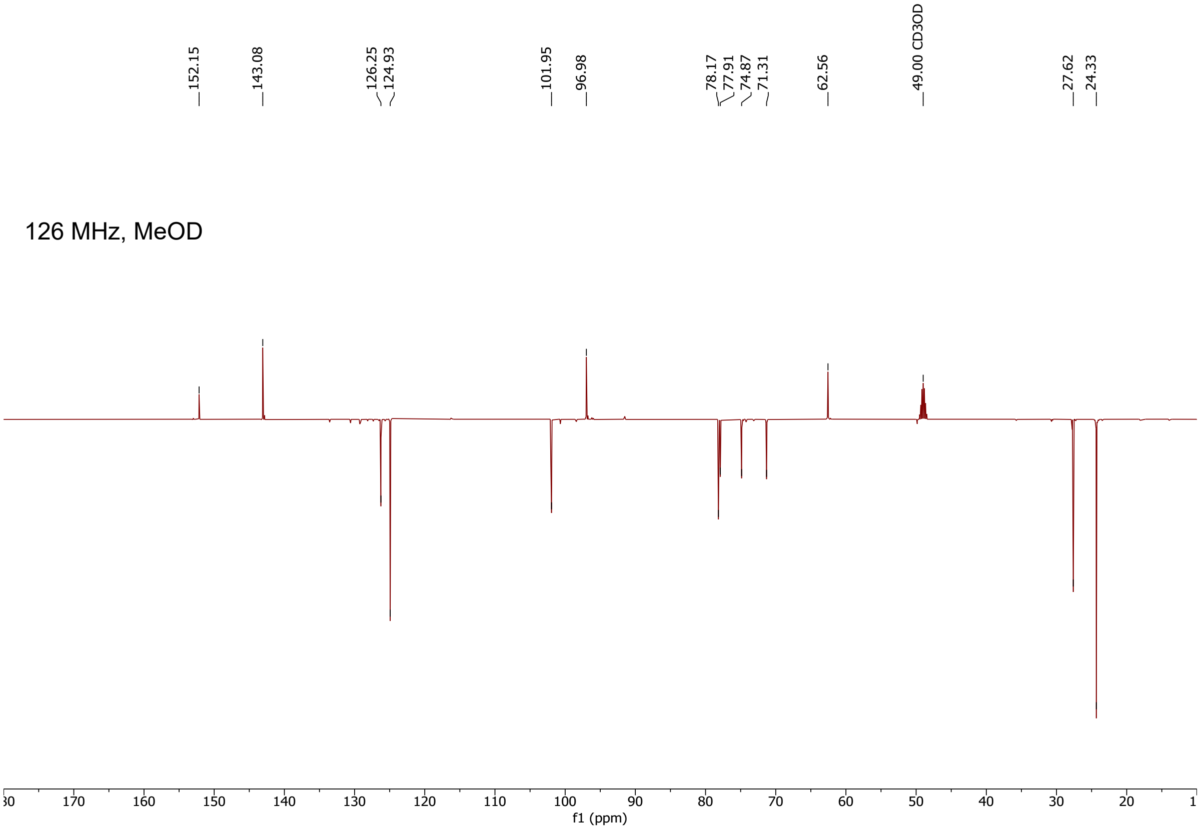


126 MHz, MeOD

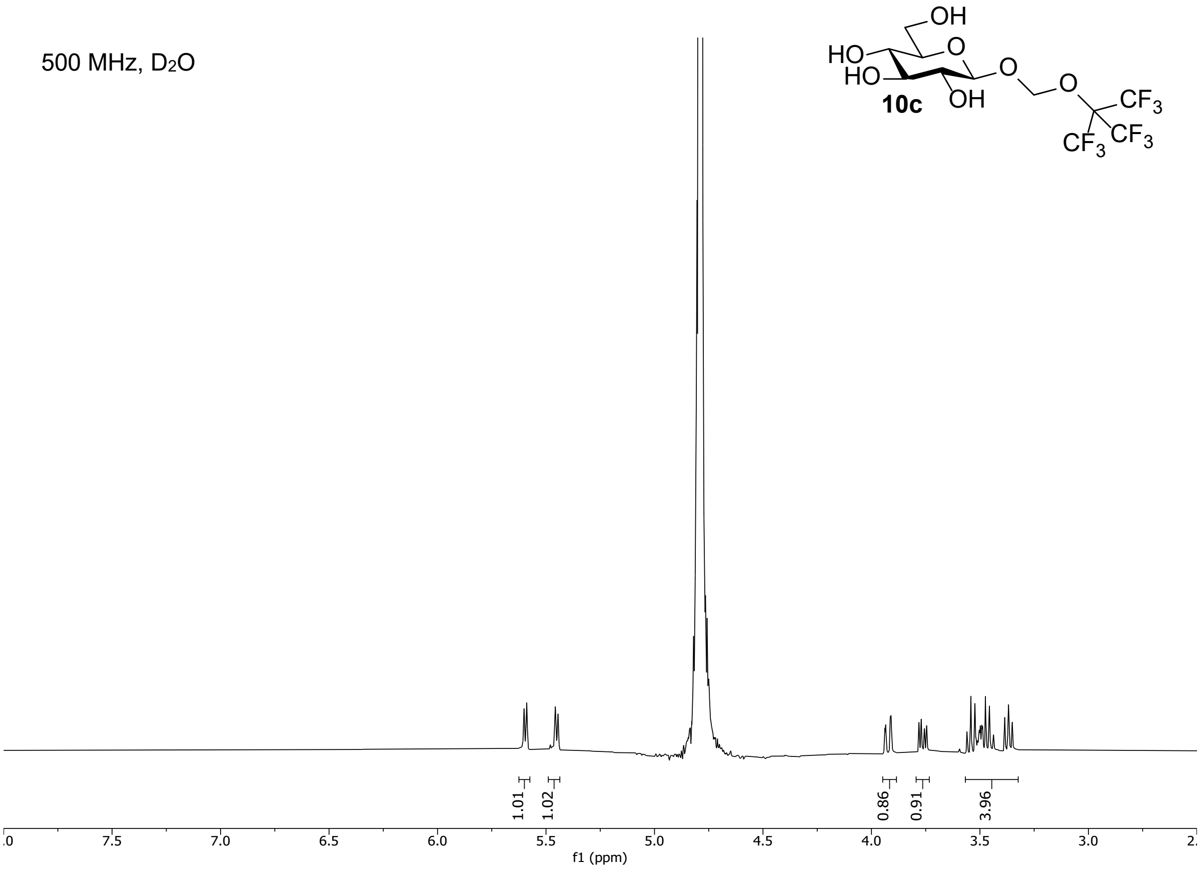
500 MHz, D2O
OH
HO
O
HO
OH
O
O
CF3
CF3
CF3
10c
1.01
1.02
0.86
0.91
3.96
f1 (ppm)

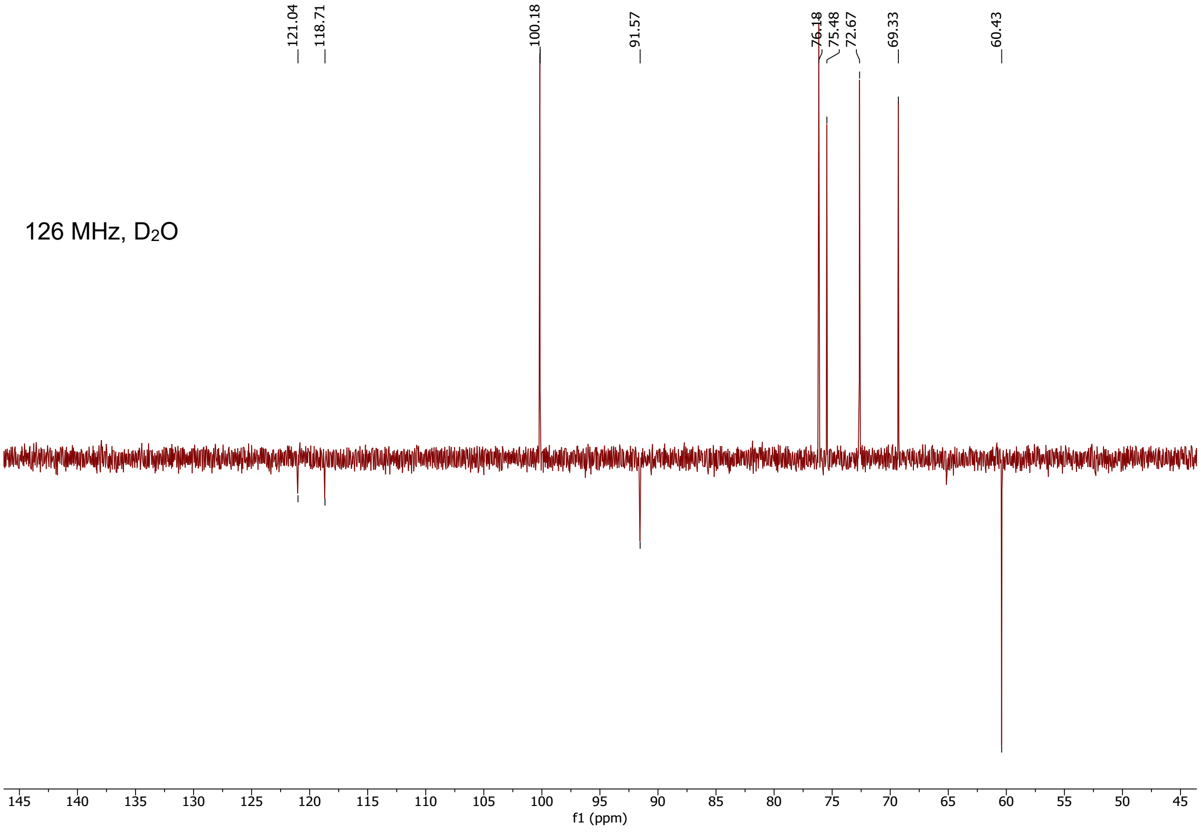
121.04
118.71
100.18
91.57
76.18
75.48
72.67
69.33
60.43
126 MHz, D2O
f1 (ppm)

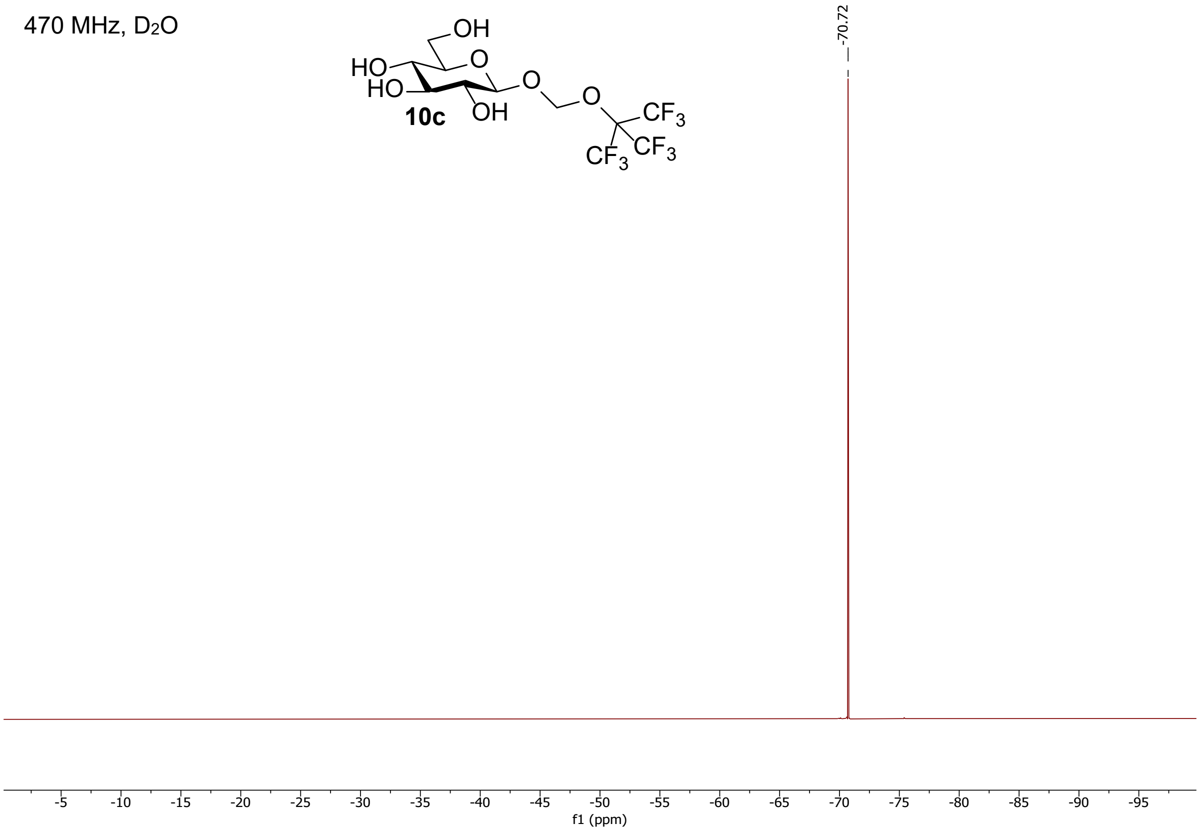
470 MHz, D2O
HO
HO
OH
O
10c
OH
O
O
CF3
CF3
CF3
-70.72
-5 -10 -15 -20 -25 -30 -35 -40 -45 -50 -55 -60 -65 -70 -75 -80 -85 -90 -95
f1 (ppm)

400 MHz, $D_2O$

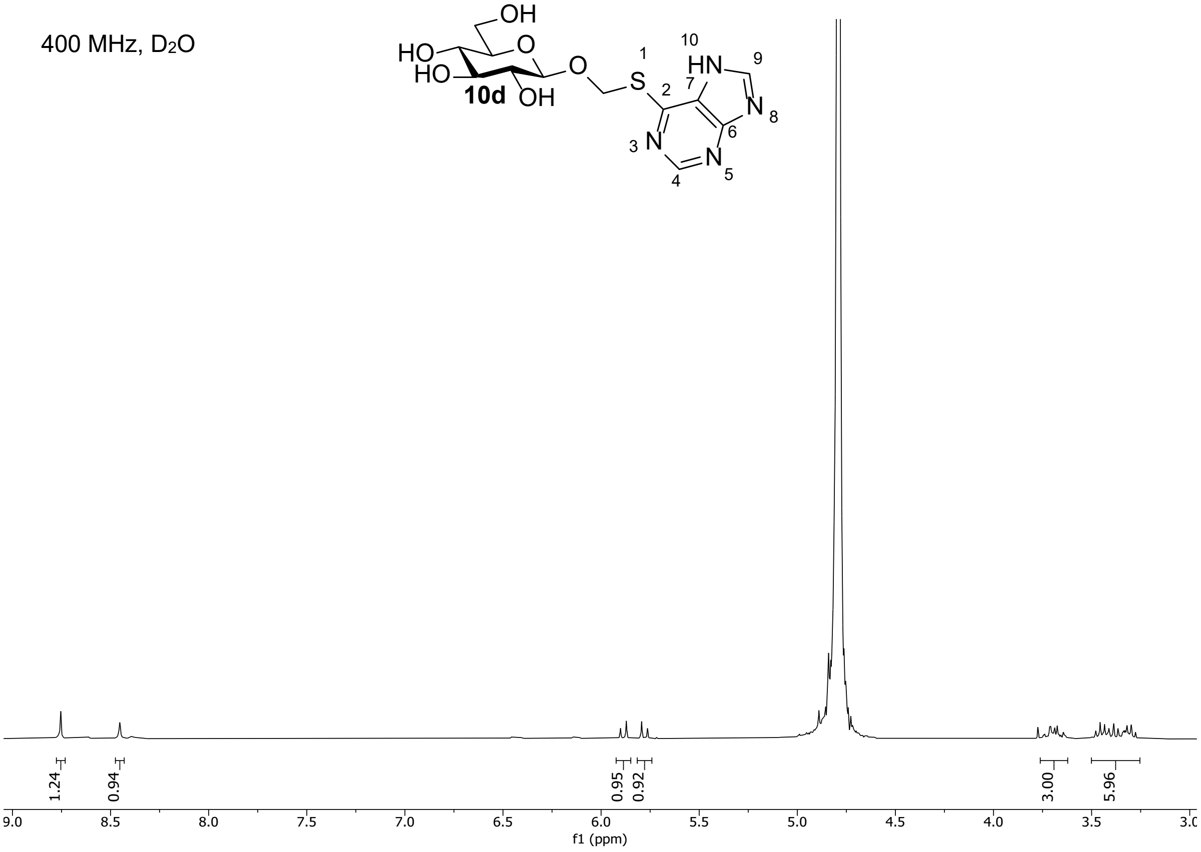


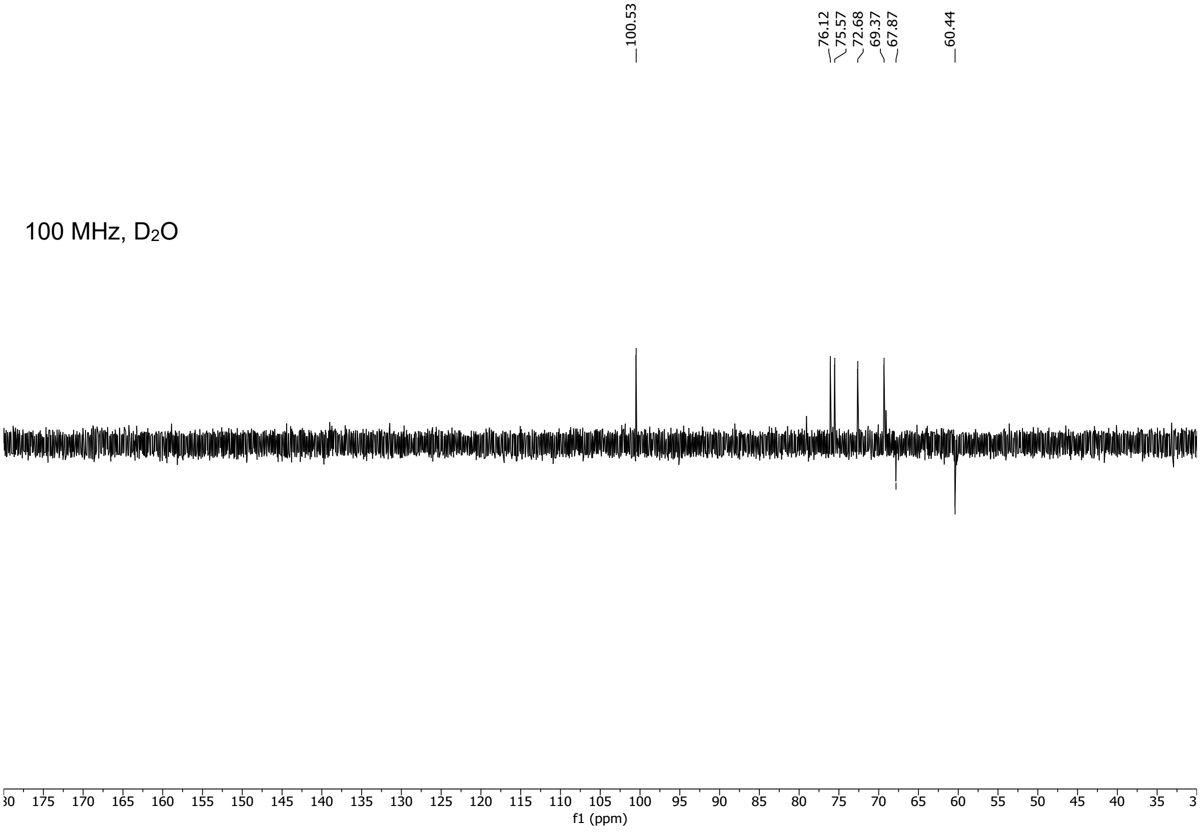


100 MHz, $D_2O$

400 MHz, $D_2O$

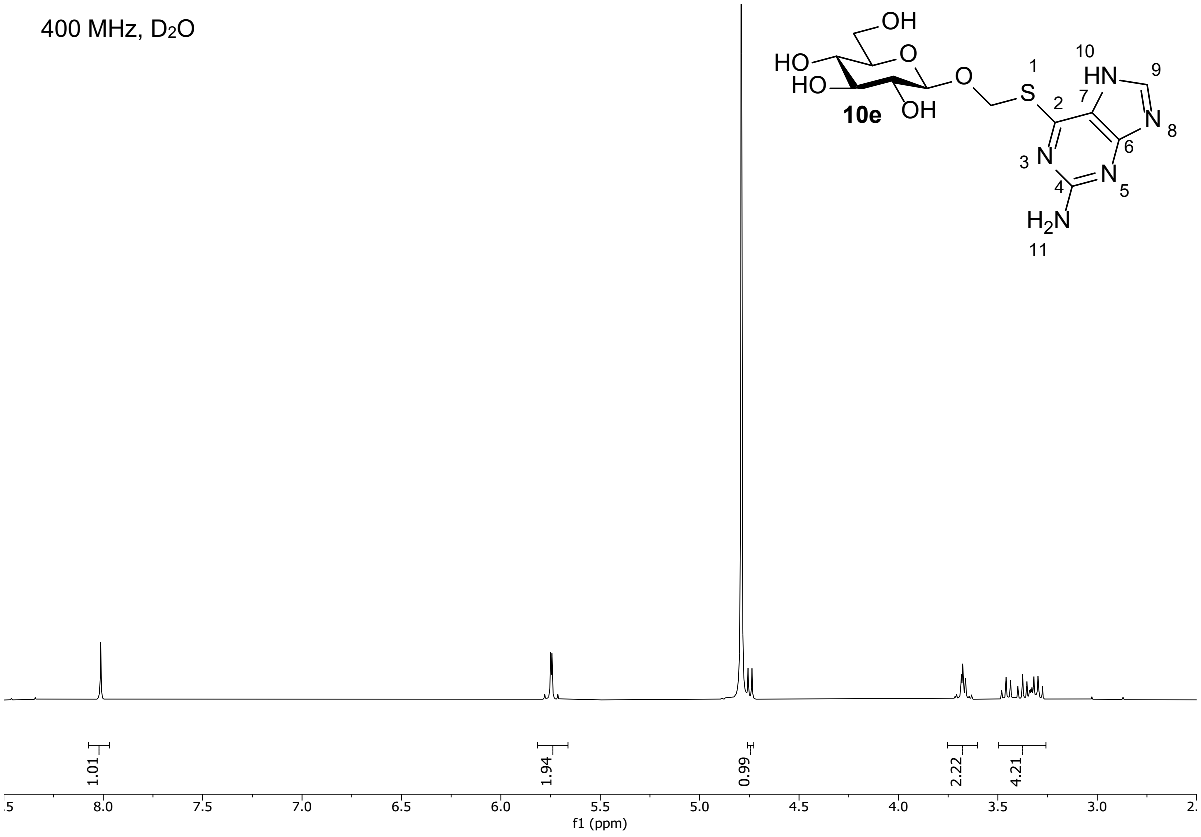


100 MHz, $D_2O$

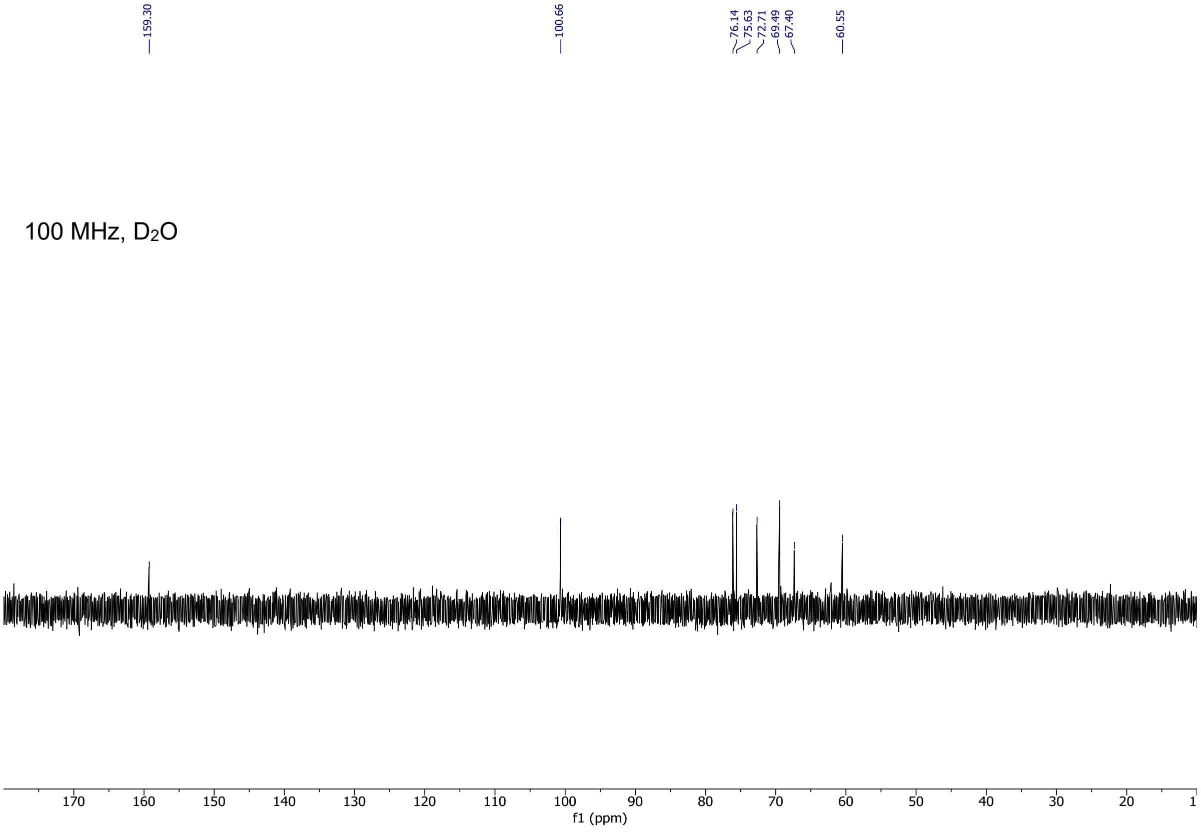

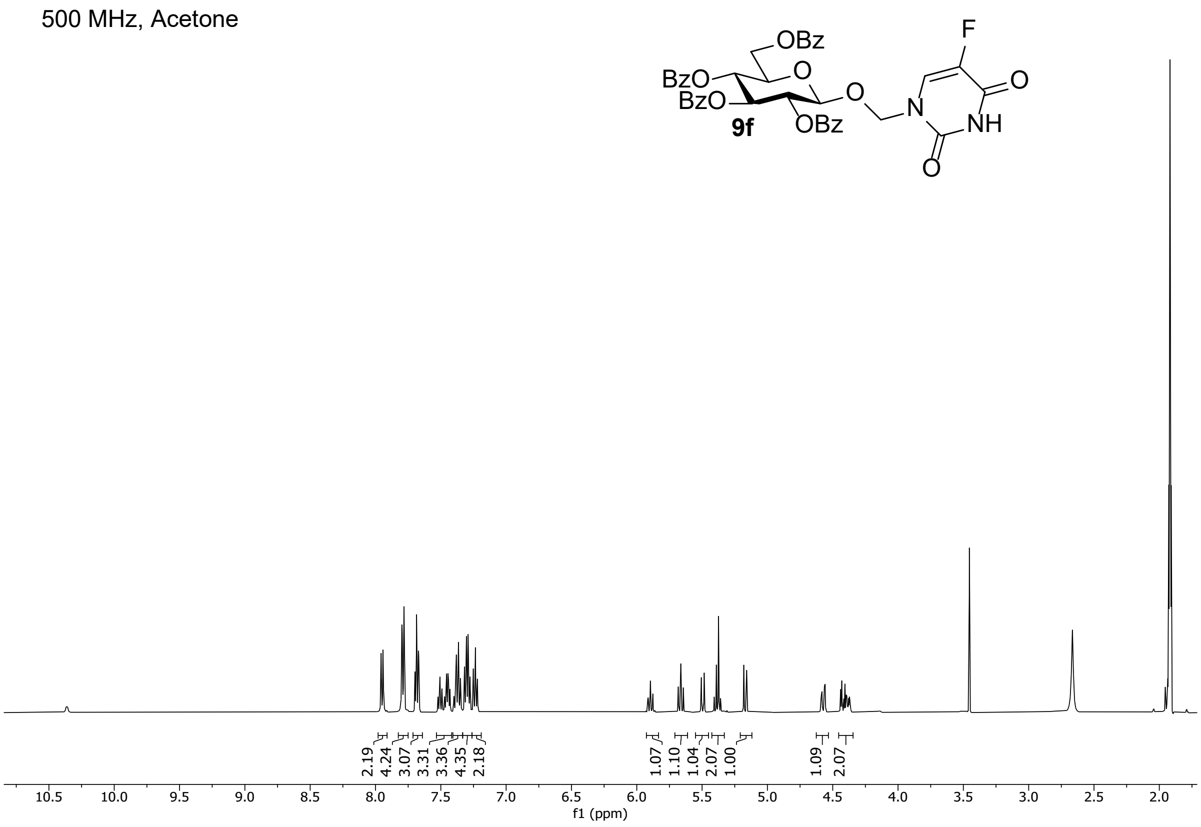
500 MHz, Acetone
OBz
BzO
BzO
O
O
N
F
O
NH
O
9f
OBz
2.19
4.24
3.07
3.31
3.36
4.35
2.18
1.07
1.10
1.04
2.07
1.00
1.09
2.07
f1 (ppm)

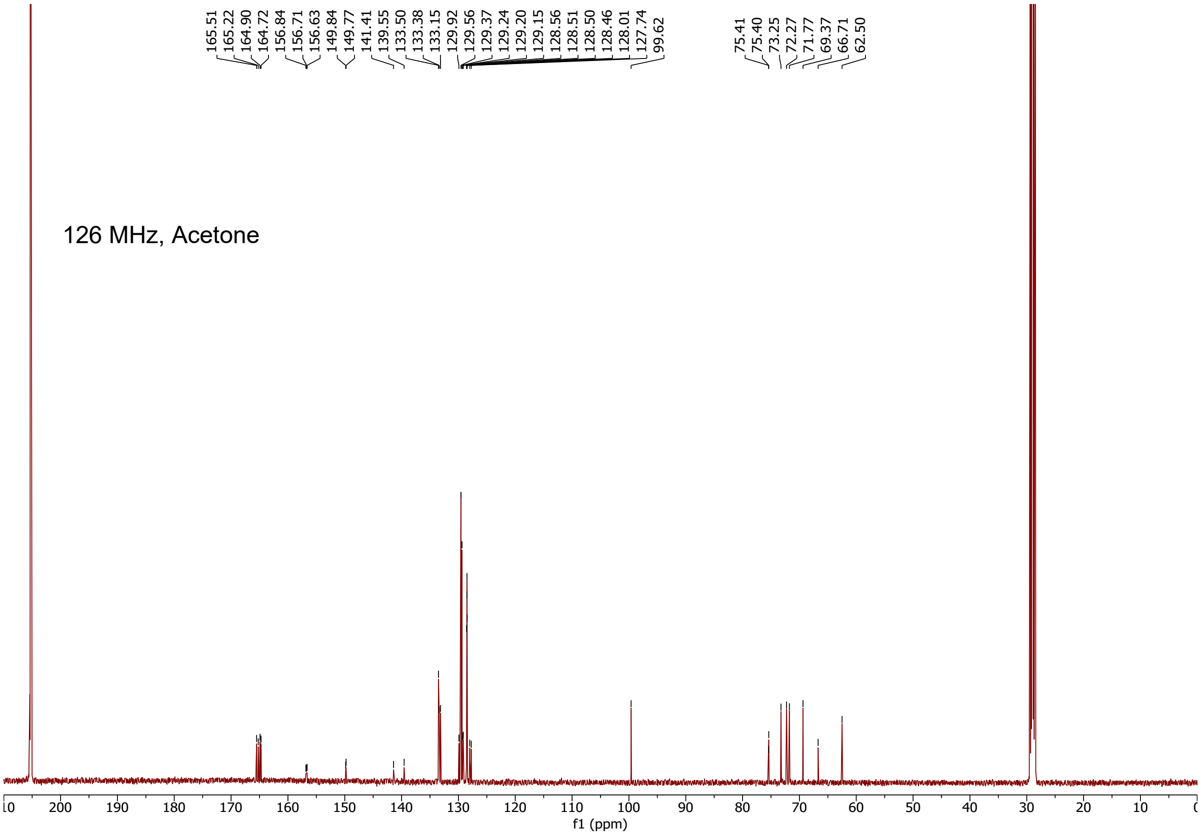
165.51
165.22
164.90
164.72
156.84
156.71
156.63
149.84
149.77
141.41
139.55
133.50
133.38
133.15
129.92
129.56
129.37
129.24
129.20
129.15
128.56
128.51
128.50
128.46
128.01
127.74
99.62
75.41
75.40
73.25
72.27
71.77
69.37
66.71
62.50
126 MHz, Acetone
f1 (ppm)

500 MHz, $CDCl_3$

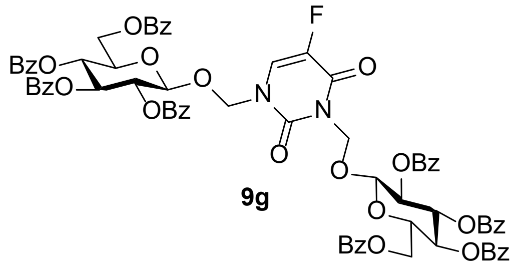


**9g**

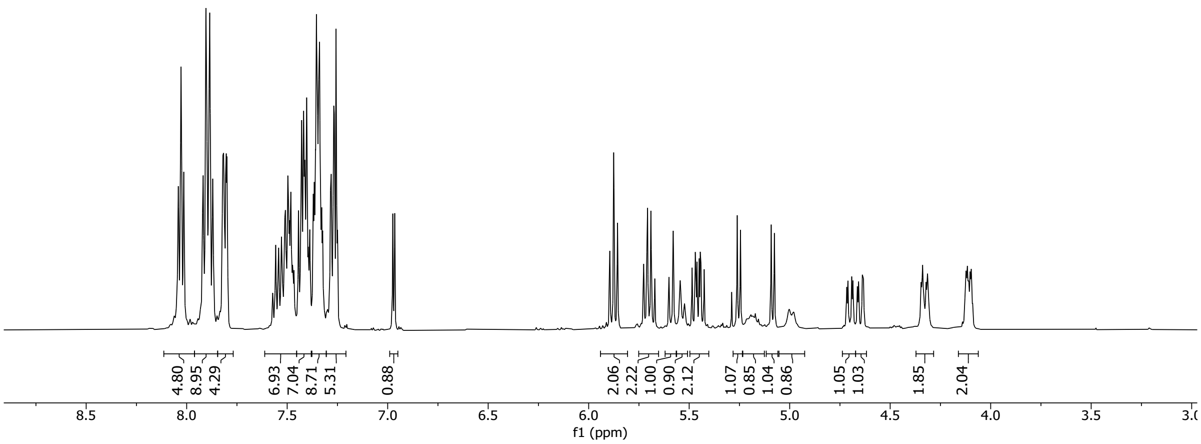


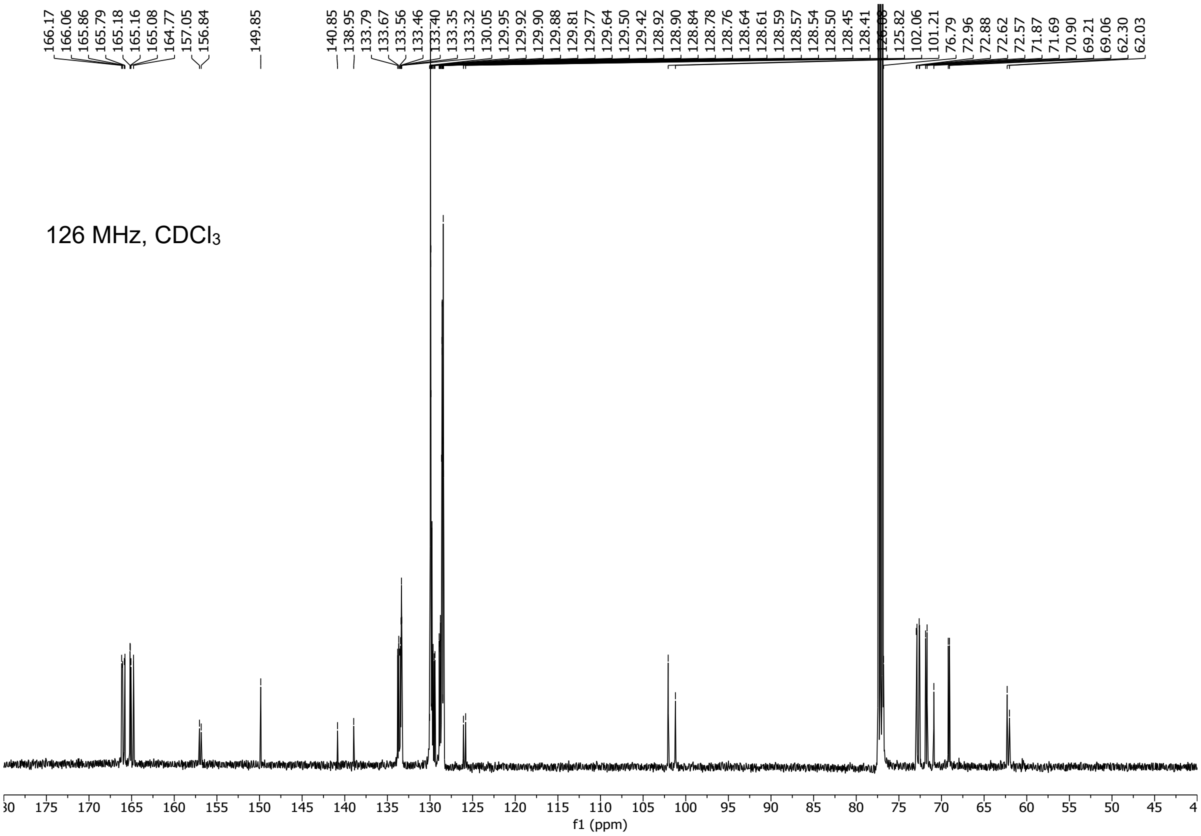


126 MHz, $CDCl_3$

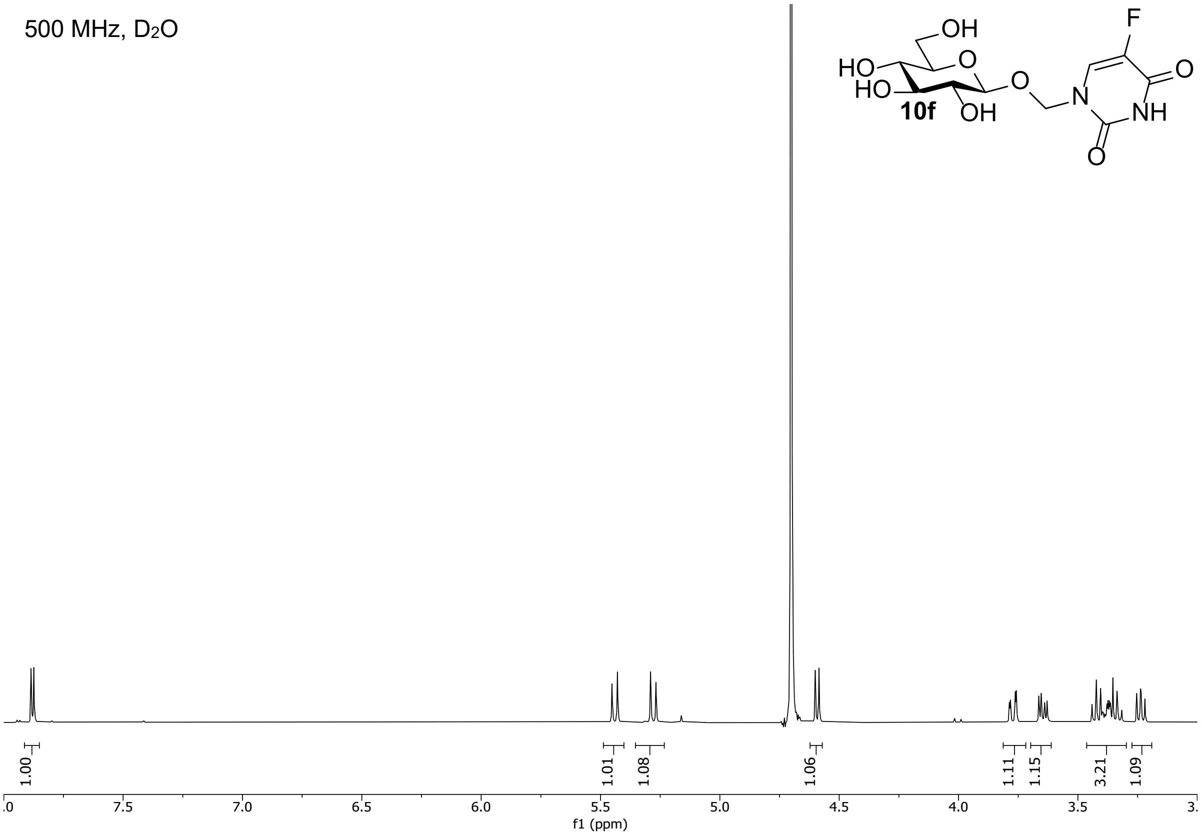
500 MHz, D2O
HO
HO
OH
O
OH
O
N
F
O
NH
O
10f
1.00
1.01
1.08
1.06
1.11
1.15
3.21
1.09
f1 (ppm)

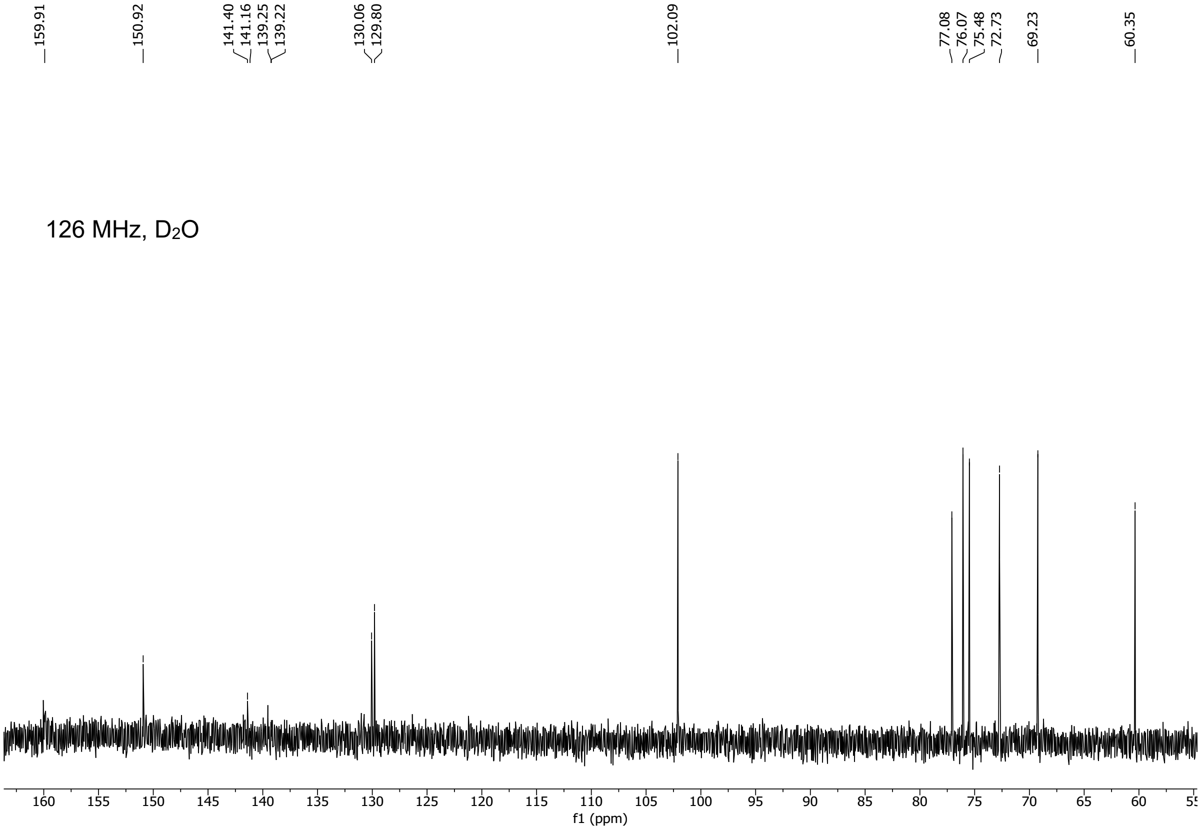
159.91
150.92
141.40
141.16
139.25
139.22
130.06
129.80
102.09
77.08
76.07
75.48
72.73
69.23
60.35
126 MHz, D2O
f1 (ppm)

500 MHz, D2O

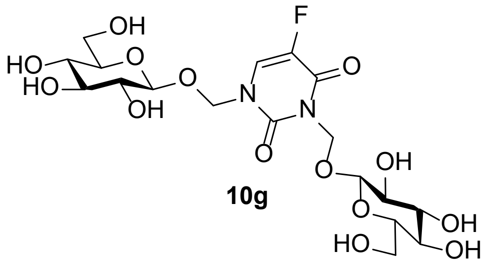

**10g**

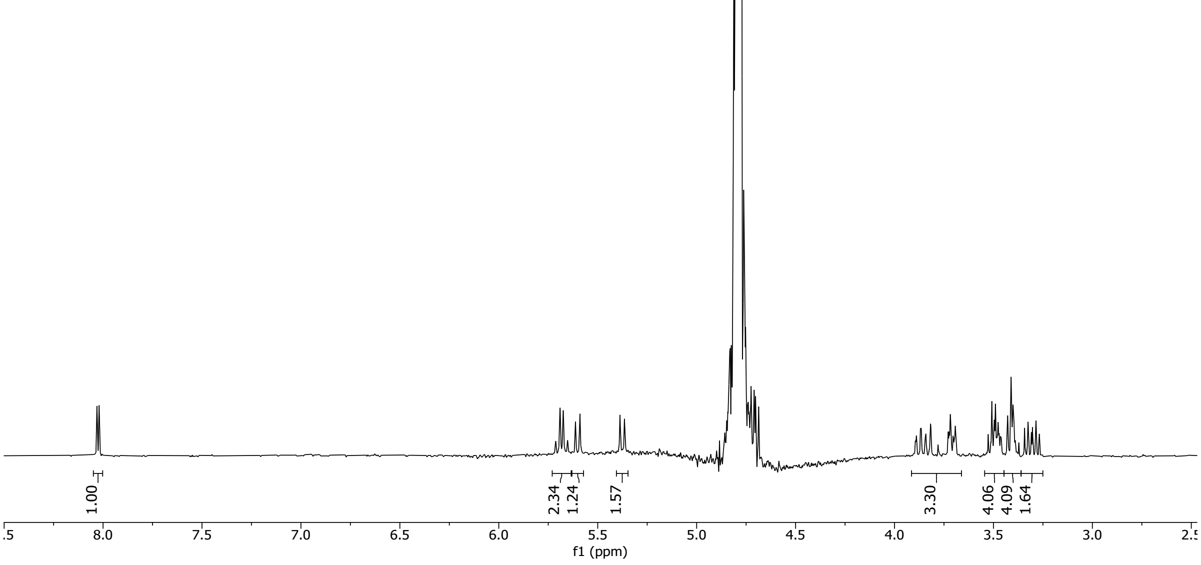


126 MHz, D2O

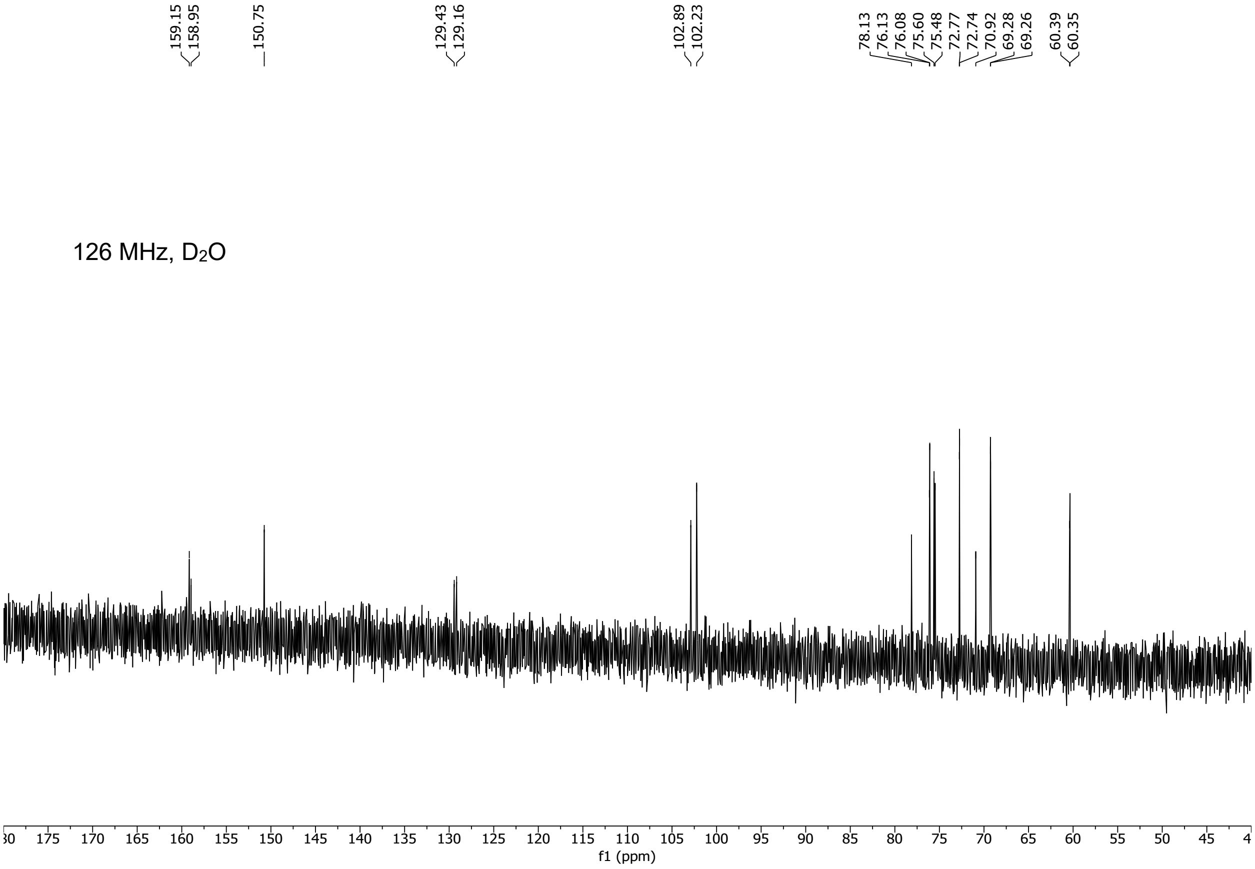

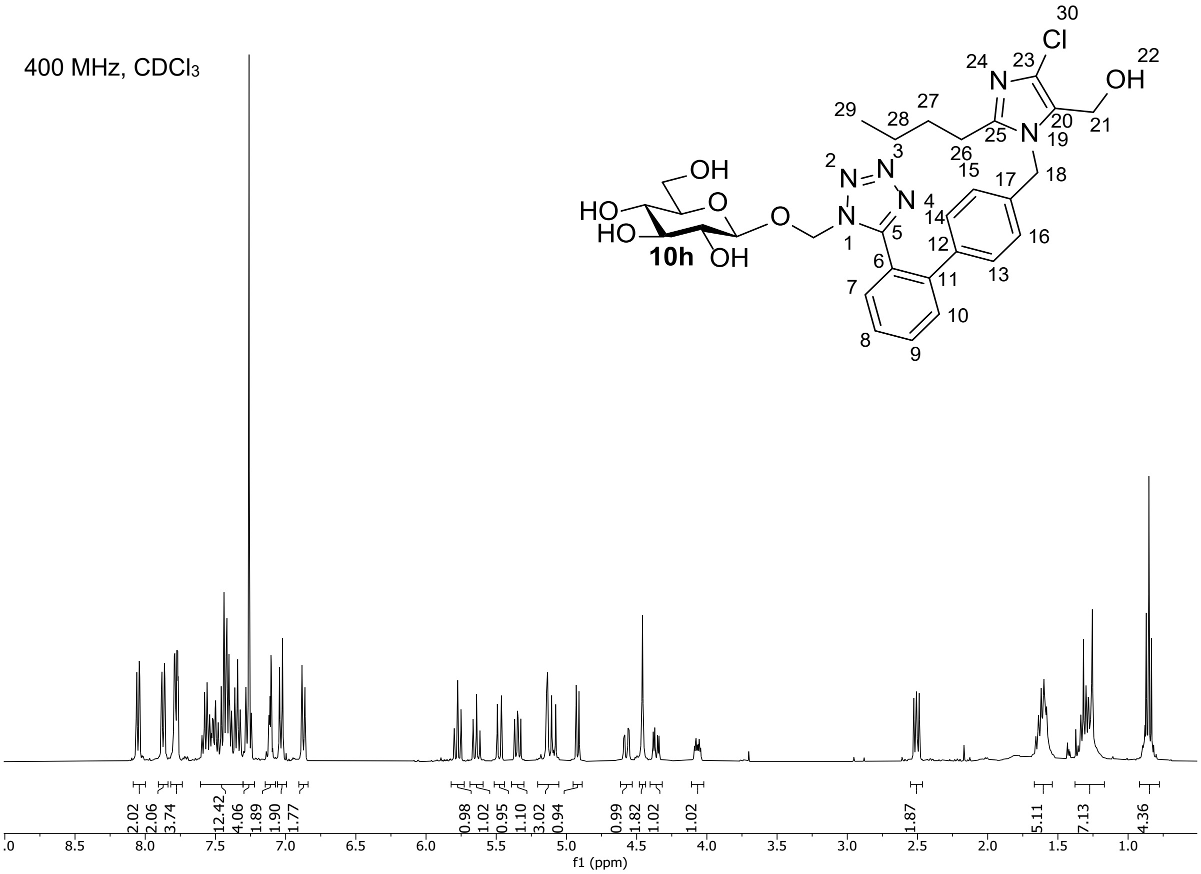
400 MHz, CDCl3
10h
HO
HO
OH
OH
O
N
Cl
OH
2.02 2.06 3.74 12.42 4.06 1.89 1.90 1.77 0.98 1.02 0.95 1.10 3.02 0.94 0.99 1.82 1.02 1.02 1.87 5.11 7.13 4.36
f1 (ppm)

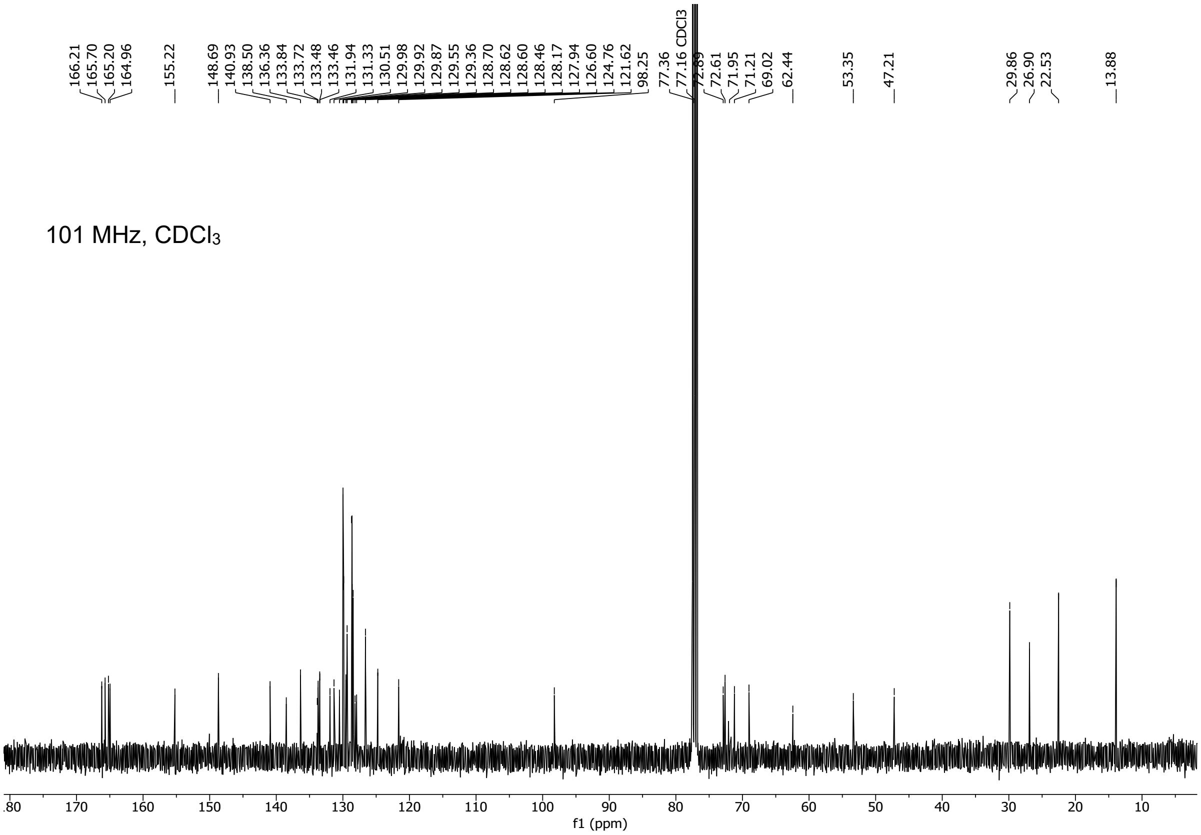
166.21 165.70 165.20 164.96 155.22 148.69 140.93 138.50 136.36 133.84 133.72 133.48 133.46 131.94 131.33 130.51 129.98 129.92 129.87 129.55 129.36 128.70 128.62 128.60 128.46 128.17 127.94 126.60 124.76 121.62 98.25 77.36 77.16 CDCl3 72.61 71.95 71.21 69.02 62.44 53.35 47.21 29.86 26.90 22.53 13.88
101 MHz, CDCl3
f1 (ppm)

500 MHz, MeOD

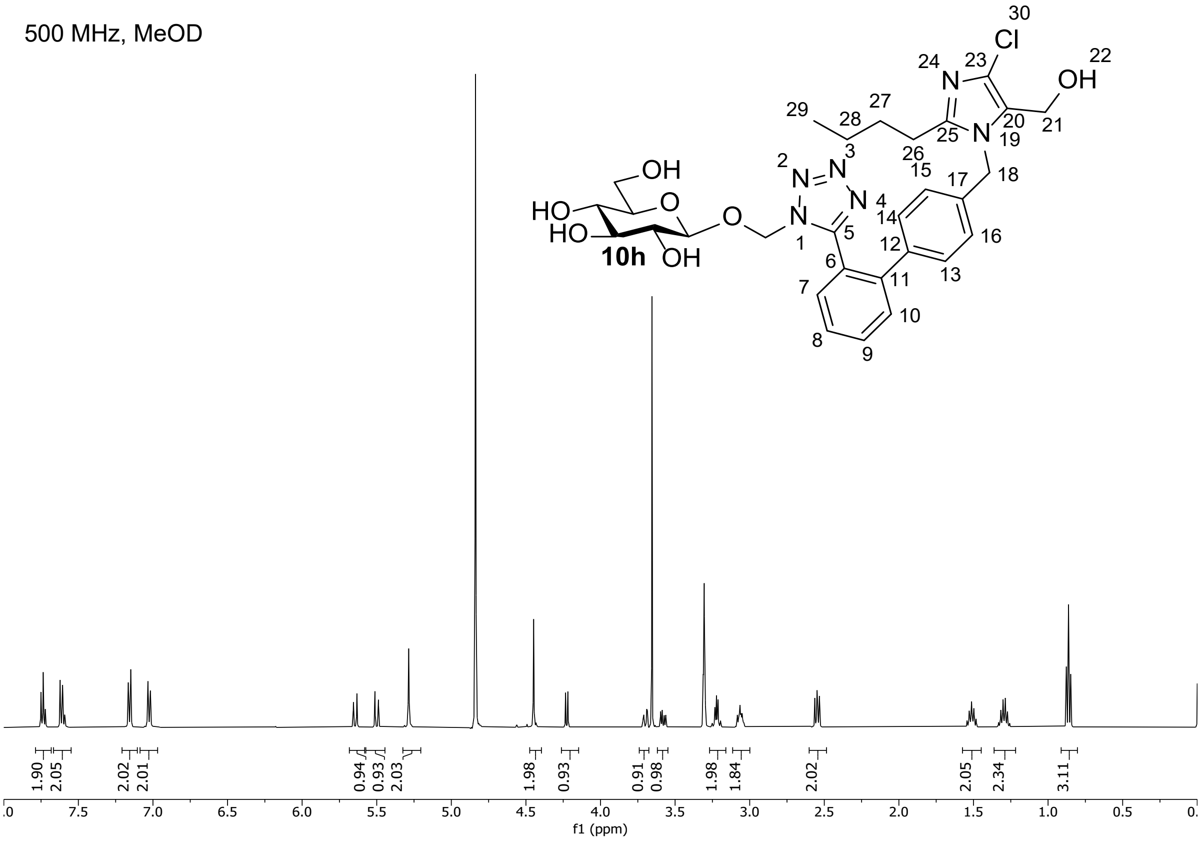

126 MHz, MeOD

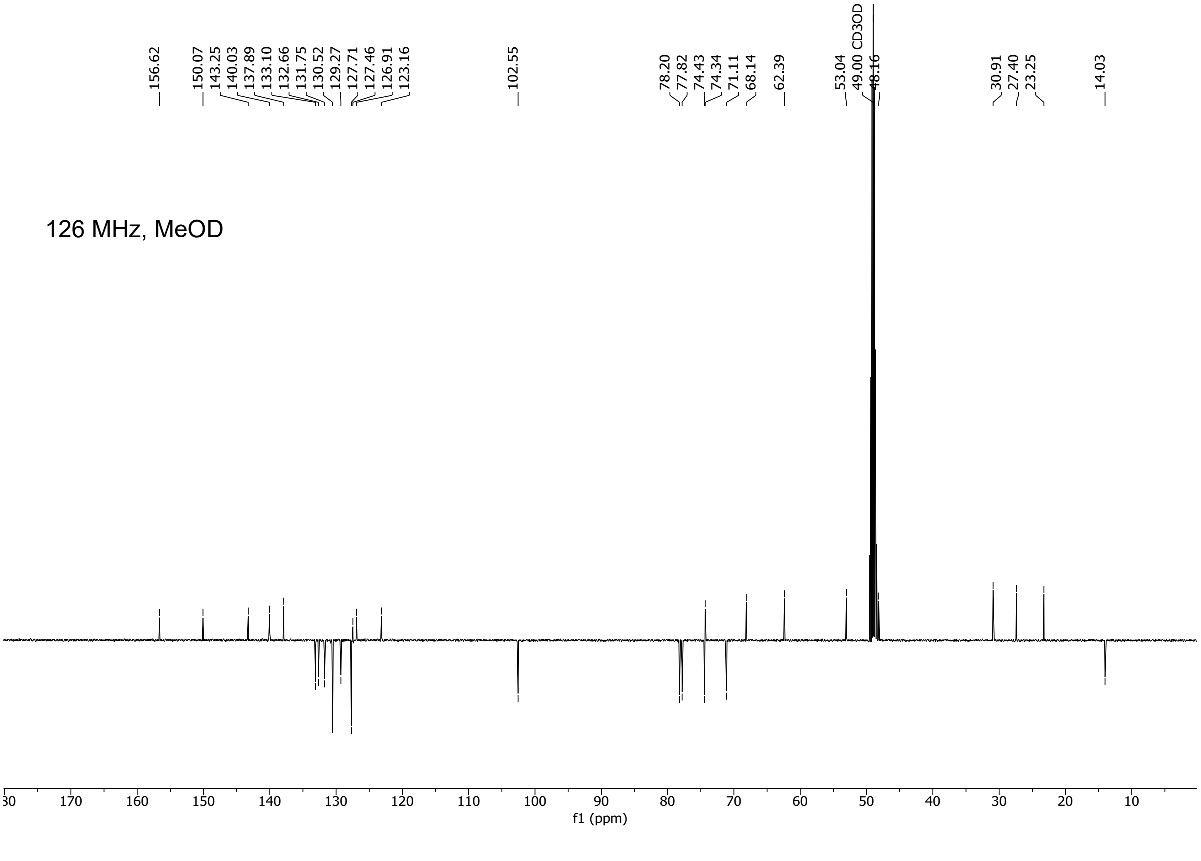

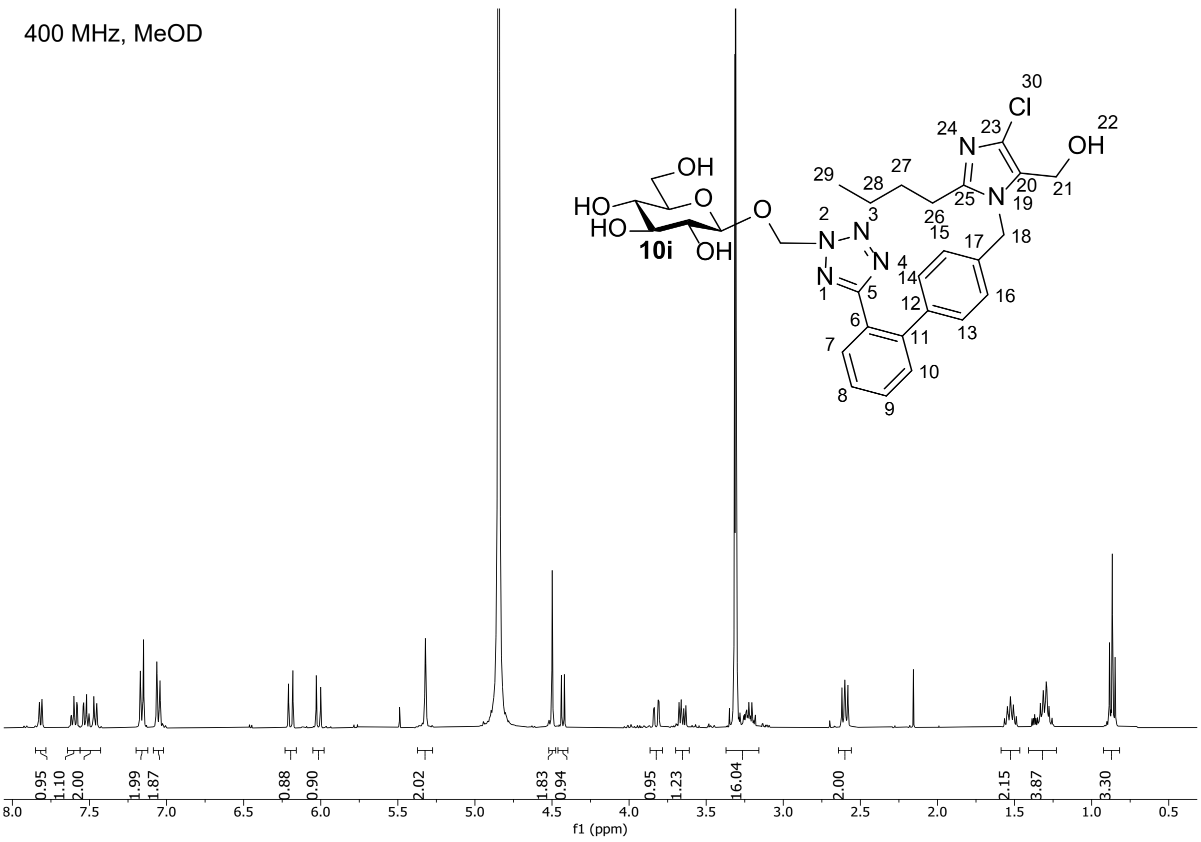
400 MHz, MeOD
10i
0.95
1.10
2.00
1.99
1.87
0.88
0.90
2.02
1.83
0.94
0.95
1.23
16.04
2.00
2.15
3.87
3.30
f1 (ppm)

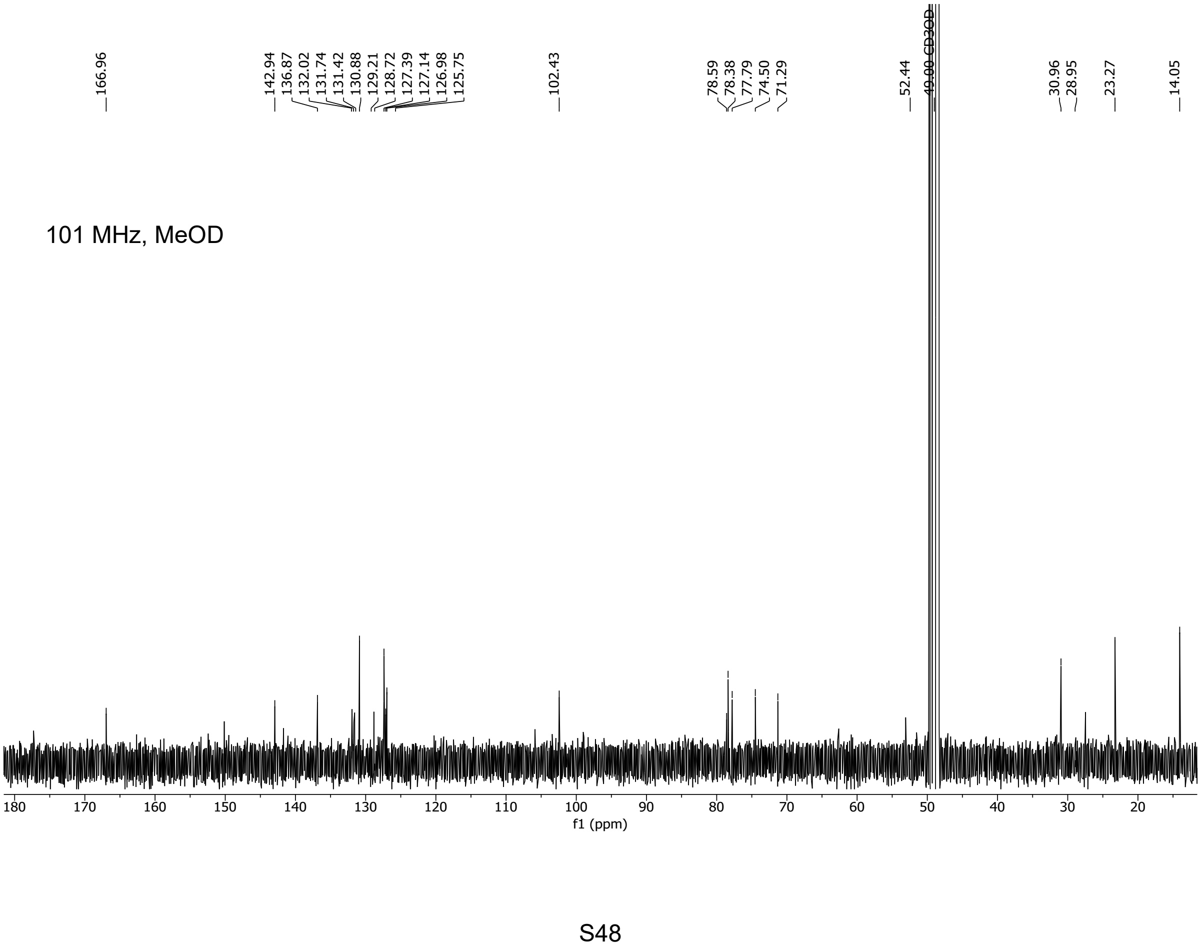
101 MHz, MeOD
166.96
142.94
136.87
132.02
131.74
131.42
130.88
129.21
128.72
127.39
127.14
126.98
125.75
102.43
78.59
78.38
77.79
74.50
71.29
52.44
49.00 CD3OD
30.96
28.95
23.27
14.05
f1 (ppm)

**HPLC traces**

**<Chromatogram>**

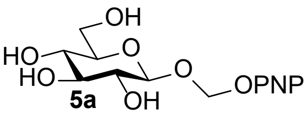


5a (Glc)

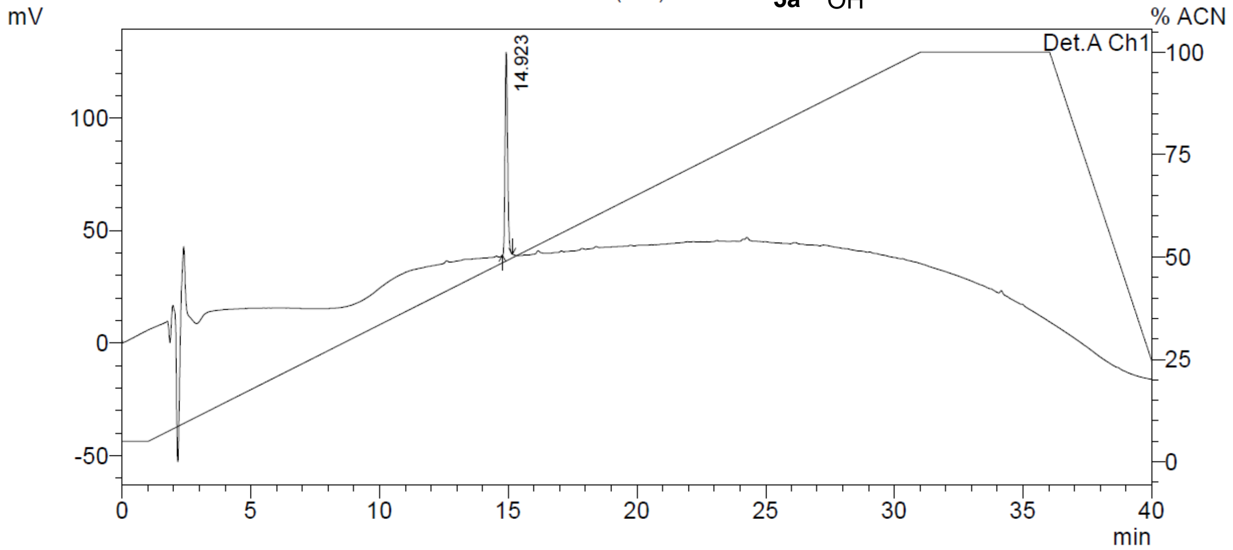


Detector A Ch1 214nm

| Peak# | Ret. Time | Area % | Area |
|---|---|---|---|
| 1 | 14.923 | 100.000 | 624497 |

**<Chromatogram>**

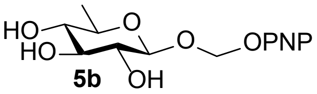


5b (Qui)

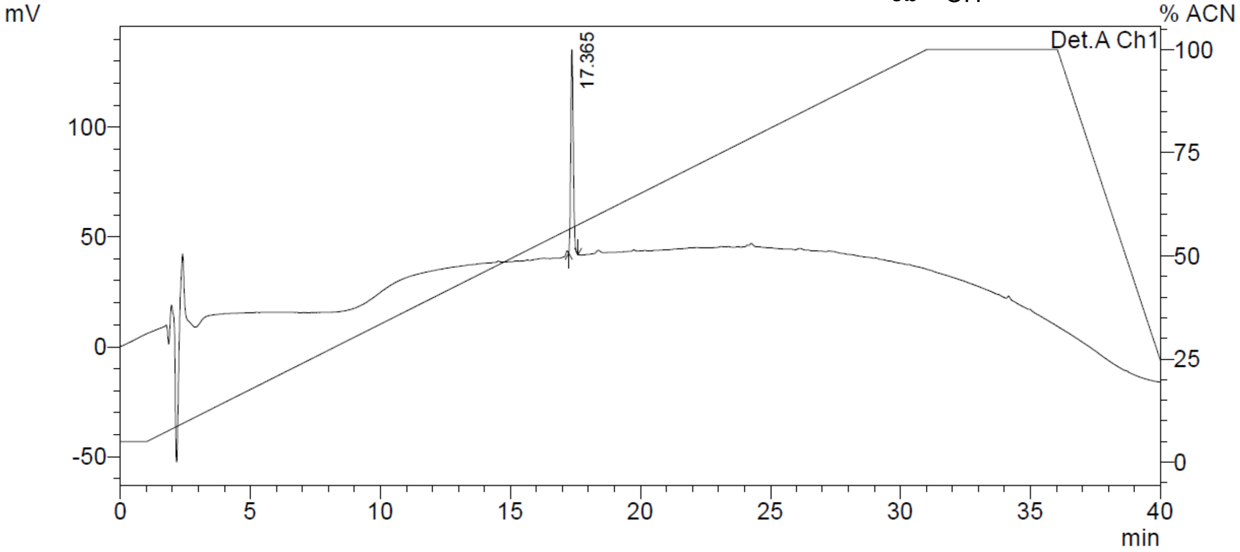


Detector A Ch1 214nm

| Peak# | Ret. Time | Area % | Area |
|---|---|---|---|
| 1 | 17.365 | 100.000 | 631208 |

## <Chromatogram>

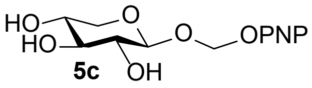


5c (Xyl)

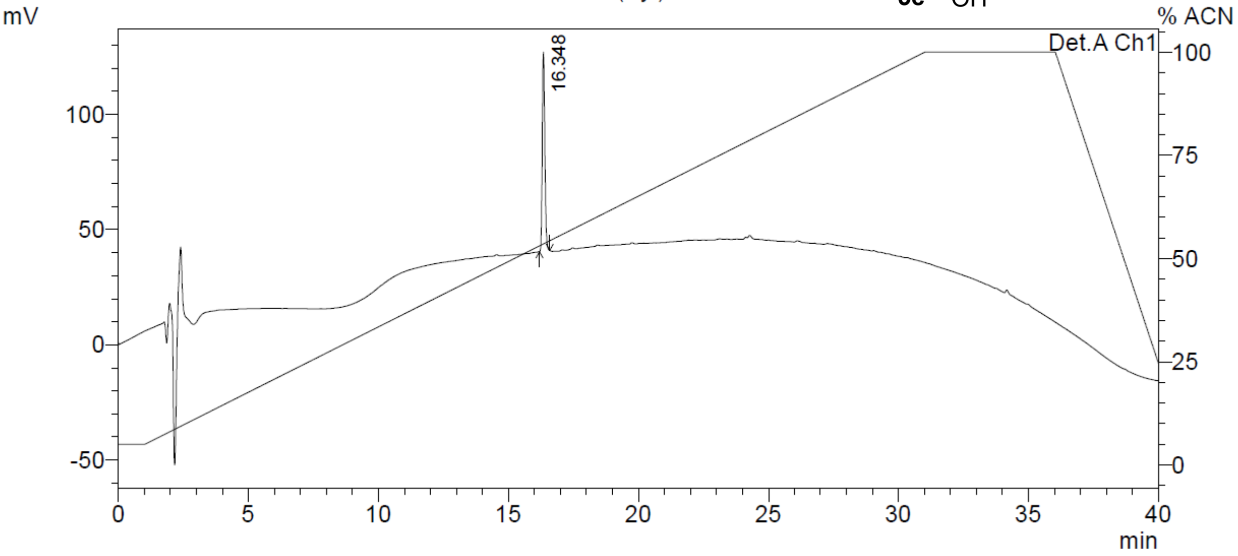


Detector A Ch1 214nm

| Peak# | Ret. Time | Area % | Area |
|---|---|---|---|
| 1 | 16.348 | 100.000 | 596685 |

## <Chromatogram>

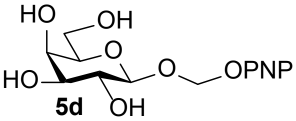


5d (Gal)

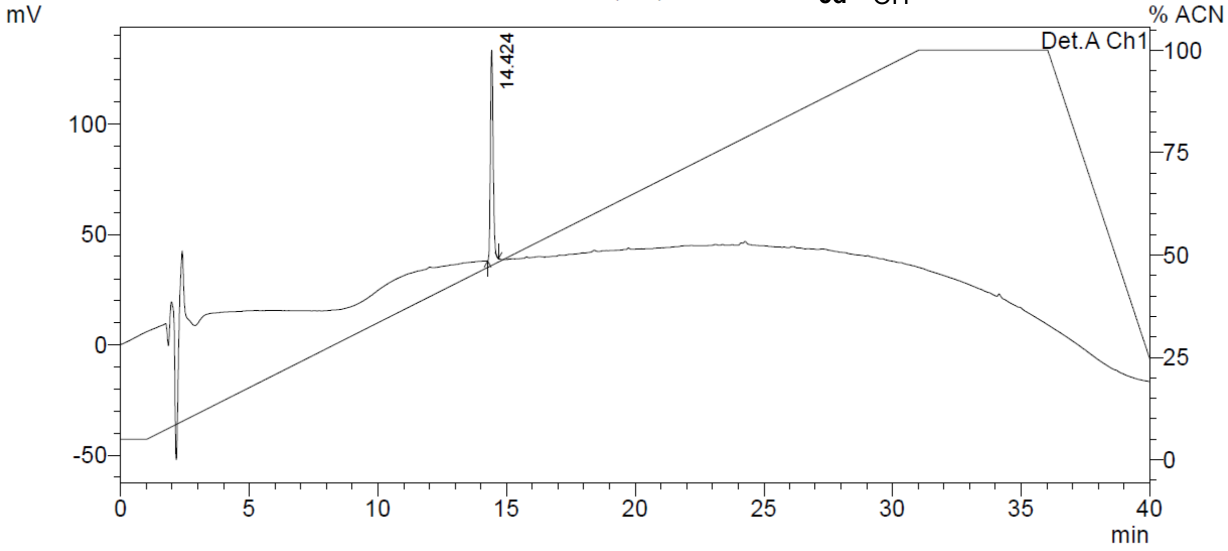


Detector A Ch1 214nm

| Peak# | Ret. Time | Area % | Area |
|---|---|---|---|
| 1 | 14.424 | 100.000 | 687959 |

**<Chromatogram>**

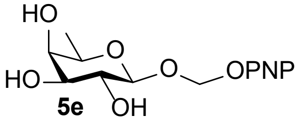


5e (Fuc)

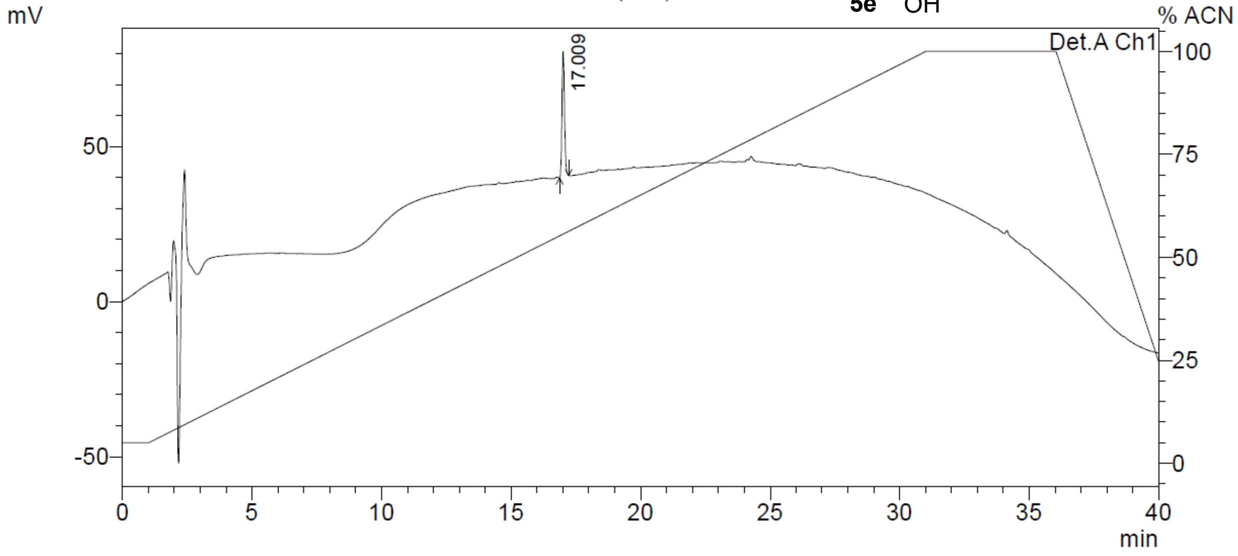


Detector A Ch1 214nm

| Peak# | Ret. Time | Area % | Area |
|---|---|---|---|
| 1 | 17.009 | 100.000 | 277149 |

**<Chromatogram>**

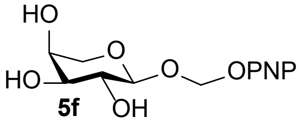


5f (Ara)

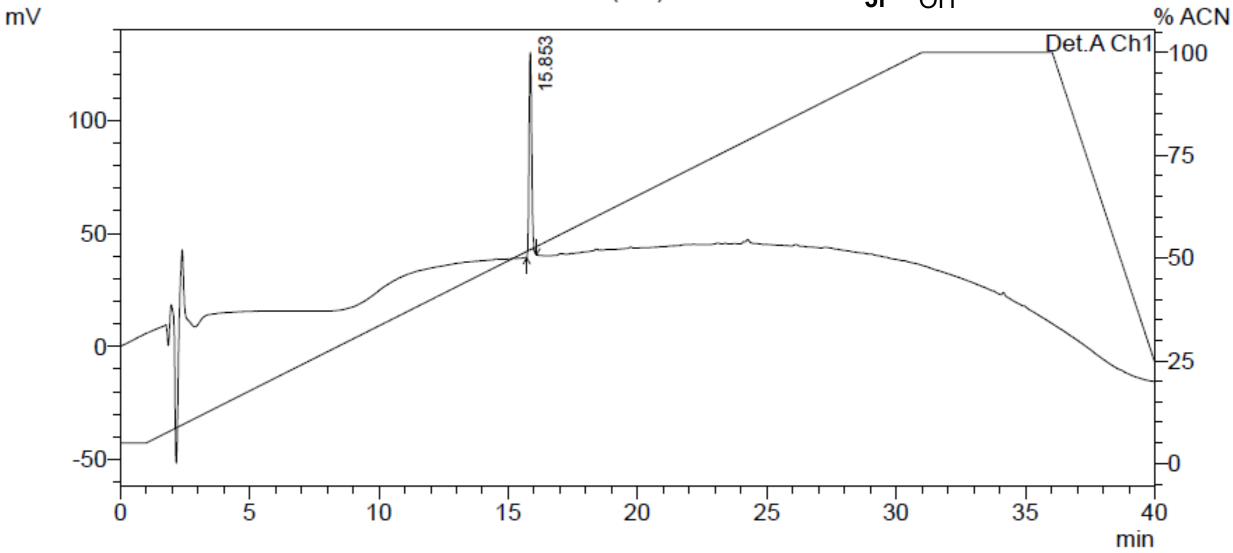


Detector A Ch1 214nm

| Peak# | Ret. Time | Area % | Area |
|---|---|---|---|
| 1 | 15.853 | 100.000 | 632098 |

**<Chromatogram>**

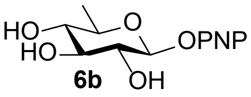

6b (Qui)

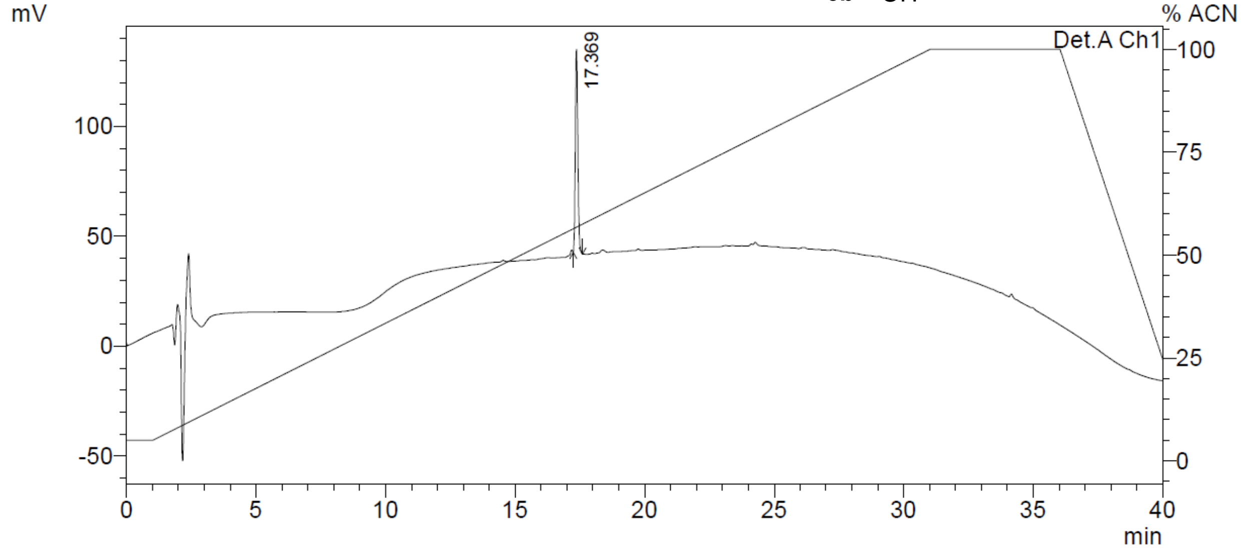


Detector A Ch1 214nm

| Peak# | Ret. Time | Area % | Area |
|---|---|---|---|
| 1 | 17.369 | 100.000 | 628315 |

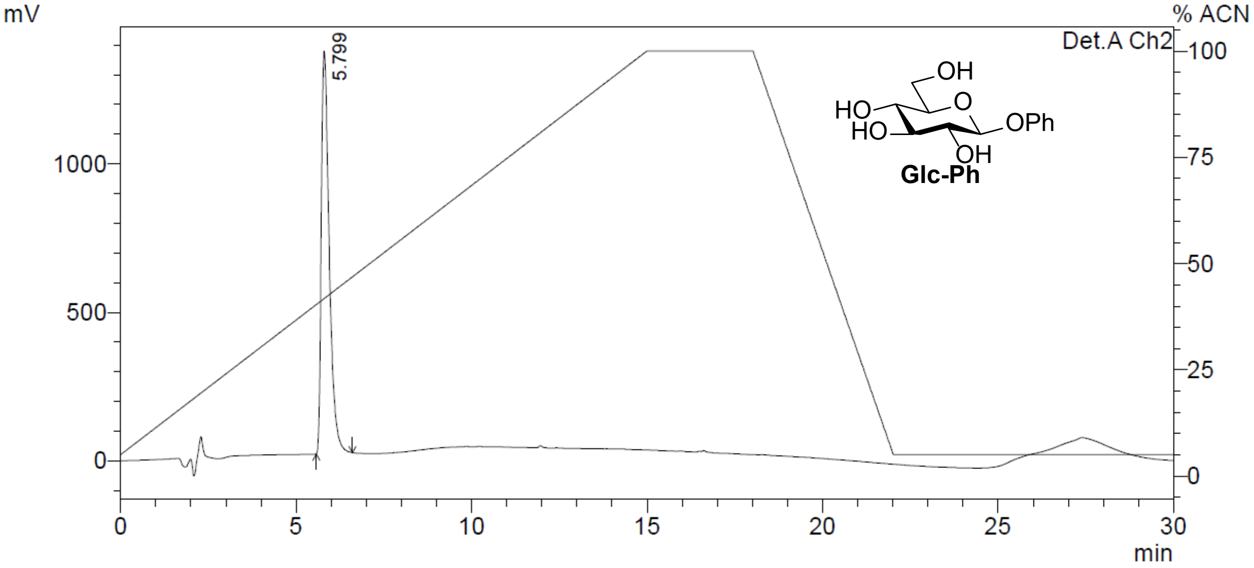


Detector A Ch2 215nm

| Peak# | Ret. Time | Area % | Area |
|---|---|---|---|
| 1 | 5.799 | 100.000 | 21297597 |

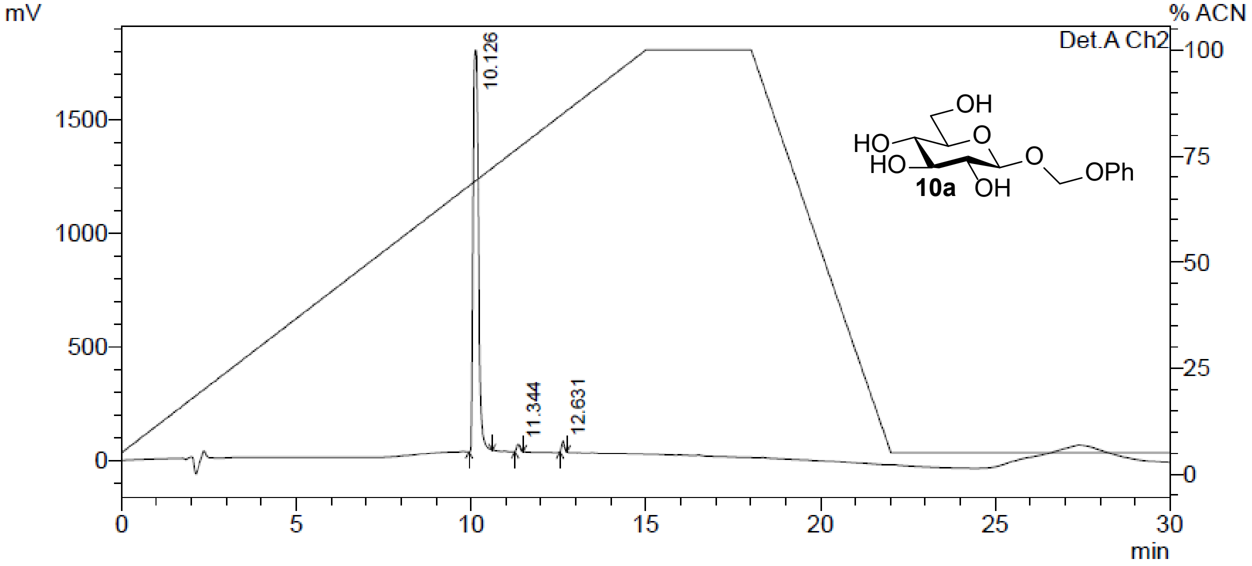


Detector A Ch2 215nm

| Peak# | Ret. Time | Area % | Area |
|---|---|---|---|
| 1 | 10.126 | 97.598 | 19025908 |
| 2 | 11.344 | 0.996 | 194092 |
| 3 | 12.631 | 1.406 | 274150 |

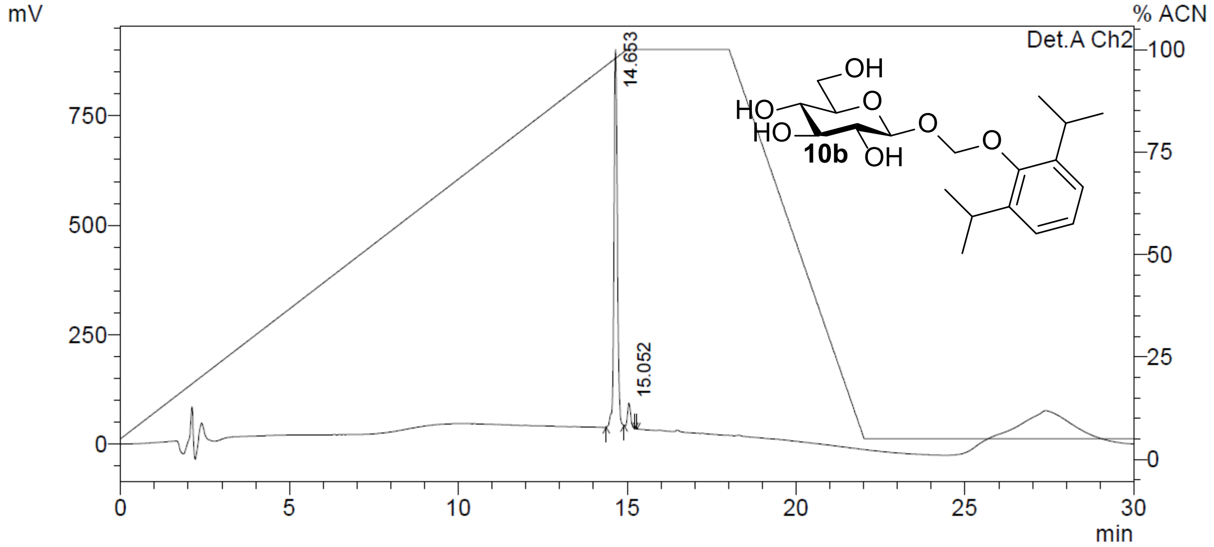


Detector A Ch2 215nm

| Peak# | Ret. Time | Area % | Area |
|---|---|---|---|
| 1 | 14.653 | 94.815 | 6099792 |
| 2 | 15.052 | 5.185 | 333555 |

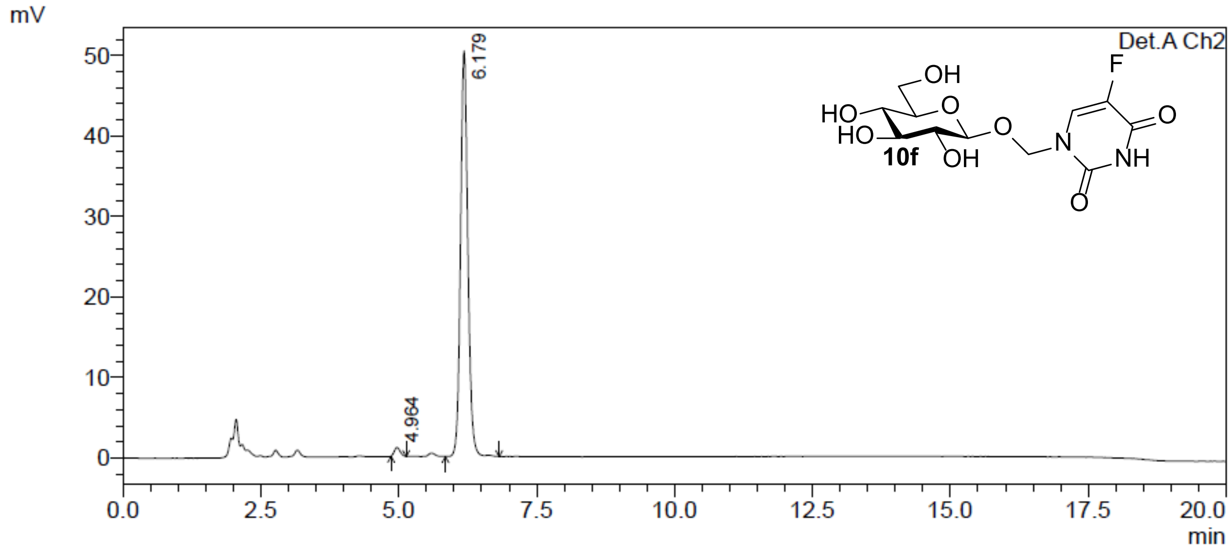


Detector A Ch2 214nm

| Peak# | Ret. Time | Area % | Area |
|---|---|---|---|
| 1 | 4.964 | 1.676 | 7970 |
| 2 | 6.179 | 98.324 | 467634 |

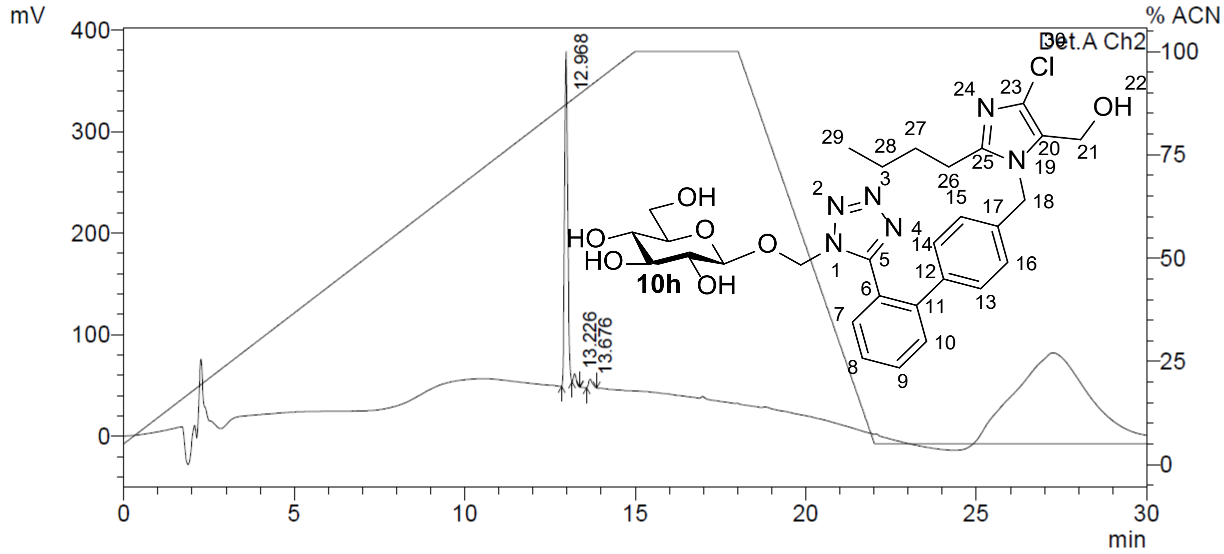


Detector A Ch2 215nm

| Peak# | Ret. Time | Area % | Area |
|---|---|---|---|
| 1 | 12.968 | 94.882 | 2197932 |
| 2 | 13.226 | 2.305 | 53403 |
| 3 | 13.676 | 2.812 | 65143 |

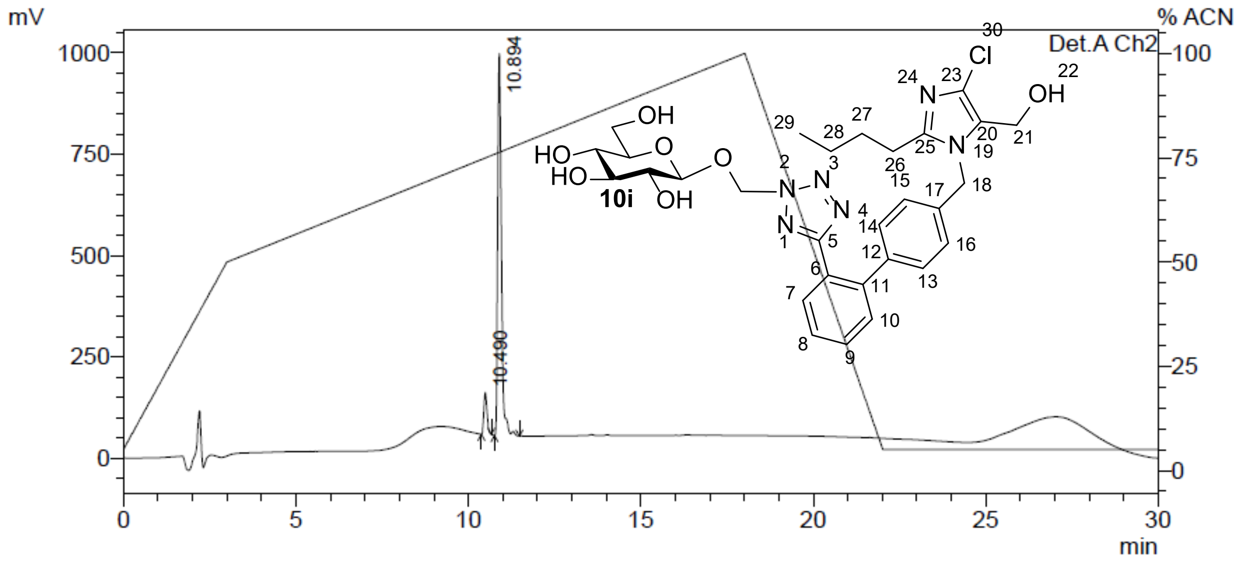


Detector A Ch2 215nm

| Peak# | Ret. Time | Area % | Area |
|---|---|---|---|
| 1 | 10.490 | 8.461 | 657206 |
| 2 | 10.894 | 91.539 | 7110468 |
